# Proteomic Analysis of the *Ehrlichia chaffeensis* Phagosome in Cultured DH82 Cells

**DOI:** 10.1371/journal.pone.0088461

**Published:** 2014-02-18

**Authors:** Yan Cheng, Yan Liu, Bin Wu, Jian-zhi Zhang, Jiang Gu, Ya-ling Liao, Fu-kun Wang, Xu-hu Mao, Xue-jie Yu

**Affiliations:** 1 Department of Clinical Microbiology and Immunology, College of Medical Laboratory, Third Military Medical University, Chongqing, China; 2 Department of Pathology, University of Texas Medical Branch, Galveston, Texas, United States of America; 3 Bethune International Peace Hospital, Shijiazhuang, China; 4 Anhui Province Center for Disease Control and Prevention, Hefei, China; 5 Jiangsu Province Center for Disease Control and Prevention, Nanjing, China; 6 School of Health Professions, University of Texas Medical Branch, Galveston, Texas, United States of America; 7 School of Public Health, Shandong University, Jinan, China; Kansas State University, United States of America

## Abstract

*Ehrlichia chaffeensis* is an obligately intracellular bacterium that resides and multiplies within cytoplasmic vacuoles of phagocytes. The *Ehrlichia*-containing vacuole (ECV) does not fuse with lysosomes, an essential condition for *Ehrlichia* to survive inside phagocytes, but the mechanism of inhibiting the fusion of the phagosome with lysosomes is not clear. Understanding the ECV molecular composition may decipher the mechanism by which *Ehrlichia* inhibits phagosome-lysosome fusion. In this study, we obtained highly purified ECVs from *E. chaffeensis*-infected DH82 cells by sucrose density gradient centrifugation and analyzed their composition by mass spectrometry-based proteomics. The ECV composition was compared with that of phagolysosomes containing latex beads. Lysosomal proteins such as cathepsin D, cathepsin S, and lysosomal acid phosphatase were not detected in *E. chaffeensis* phagosome preparations. Some small GTPases, involved in membrane dynamics and phagocytic trafficking, were detected in ECVs. A notable finding was that Rab7, a late endosomal marker, was consistently detected in *E. chaffeensis* phagosomes by mass spectrometry. Confocal microscopy confirmed that *E. chaffeensis* phagosomes contained Rab7 and were acidified at approximately pH 5.2, suggesting that the *E. chaffeensis* vacuole was an acidified late endosomal compartment. Our results also demonstrated by mass spectrometry and immunofluorescence analysis that *Ehrlichia* morulae were not associated with the autophagic pathway. *Ehrlichia chaffeensis* did not inhibit phagosomes containing latex beads from fusing with lysosomes in infected cells. We concluded that the *E. chaffeensis* vacuole was a late endosome and *E. chaffeensis* might inhibit phagosome-lysosome fusion by modifying its vacuolar membrane composition, rather than by regulating the expression of host genes involved in trafficking.

## Introduction

*Ehrlichia chaffeensis* is an obligately intracellular, Gram-negative bacterium with tropism for monocytes and macrophages [1]. *E. chaffeensis* causes human monocytic ehrlichiosis (HME), an emerging life-threatening tick-borne zoonosis. The primary function of the professional phagocyte is destruction of engulfed bacteria. When a bacterial pathogen is engulfed by a phagocyte, the cell membrane surrounding the bacterium is invaginated and separated from the plasma membrane to form a phagosome [2], [3], [4], [5], [6]. The phagosome undergoes a series of fusions with early endosome, late endosome, and lysosome, which leads to the formation of the phagolysosome and degradation of the ingested particles [7], [8], [9]. However, *E. chaffeensis* resides and replicates in a cytoplasmic vacuole, which is designated as *Ehrlichia-*containing vacuole (ECV) in this study. The key strategy utilized by *E. chaffeensis* to survive inside the host cell is to prevent lysosomal fusion with the ECV. Although previous studies have demonstrated that *E. chaffeensis* resides in early endosomes that do not fuse with lysosomes [10], neither the ECV molecular composition nor the mechanism of preventing phagosome-lysosome fusion by *E. chaffeensis* is clear. Elucidating the molecular composition of the *E. chaffeensis* vacuoles is essential for understanding the mechanism by which *E. chaffeensis* inhibits lysosomal fusion with the phagosome.

In this study, we purified ECVs from infected canine macrophages, analyzed the proteomics of the ECVs and compared the composition of the ECVs with that of the phagolysosomes containing latex beads.

## Materials and Methods

### Cultivation of *E. chaffeensis* and preparation of antigen slides

*E. chaffeensis* was cultivated in DH82 cells [11], a canine monocyte-macrophage cell line, in minimum essential medium (MEM) supplemented with 10% newborn calf serum at 37°C in air containing 5% CO_2_. The bacterial infection in DH82 cells was assessed by staining of the infected cells with Diff-Quik Kit (Baxter Scientific Products, Obetz, Ohio). To prepare cell-free *Ehrlichia* inocula, *E. chaffeensis-*infected cells in a T25 flask were removed using a cell scraper, and then harvested by centrifugation at 400×g for 10 min. The pellets were resuspended in 5 ml of serum-free MEM, and the cells were disrupted with glass beads by vortexing twice for 30 s. The cell debris and unbroken cells were removed by centrifugation at 200×g for 10 min. The supernatant was passed through a 5 µm pore-size syringe filter (Millex, Sigma-Aldrich, St. Louis, MO, USA). Cell-free *E. chaffeensis* were inoculated on each well of Labtech II 8-well chamber slides (Nalgene Nunc, Naperville, IL, USA), which contained a monolayer of DH82 cells. The 8-well chamber slides were incubated at 37°C for 3 days in a 5% CO_2_ atmosphere. *E. chaffeensis*-infected cells were permeabilized with acetone and methanol at a ratio of 1∶1 for 10 minutes at −20°C. The slides were kept at −20°C until testing and rehydrated for 1 h at room temperature with PBS before use.

### Co-infection of DH82 cells with *E. chaffeensis* and formalin-fixed *Escherichia coli*

The *E. coli* (Top10 strain, Invitrogen, Carlsbad, CA, USA) were cultivated overnight at 37°C and centrifuged at 400×g for 15 min. The pellets were fixed in 10% formalin at room temperature overnight and kept at 4°C until use.

Host cell-free *E. chaffeensis* were inoculated (multiplicity of infection of 100∶1, bacteria to macrophage) in each well of Labtech II 8-well chamber slides and incubated for 90 min at 37°C to allow for internalization. Non-ingested *E. chaffeensis* were washed away, and the slides were incubated for an additional 3 days. On day 3 post inoculation (pi), formalin-fixed *E. coli* were then added to each well of the slides and incubated for 90 min at 37°C. After removing extracellular *E. coli*, cells were further incubated for 30 min at 37°C. LysoTracker Red (Invitrogen, Eugene, OR, USA) was diluted to the final working concentration in the medium. The prewarmed (37°C) LysoTracker Red-containing medium was added to each well of the slides. The cells were incubated at 37°C for 1 h, and then stained with DAPI at room temperature for 30 min before examination by confocal microscopy.

### Primary and secondary antibodies

Goat antibodies to cathepsin D (sc-6486), CD71 (sc-7088), EEA1 (sc-6414), v-ATPase (sc-20949), Rab5C (sc-26571), Rab7A (sc-6563) and LAMPII (sc-8101) were purchased from Santa Cruz Biotechnology (Dallas, TX, USA). Rabbit antibodies to Beclin-1 and MAP LC3 (APG8a) were purchased from Abgent (San Diego, CA, USA). All antibodies were diluted at 1∶100 for use. The conjugated antibodies included Alexa fluor 647-labeled anti-rabbit IgG (H+L) and Alexa fluor 647-labeled anti-goat IgG (H+L) (Molecular Probes Invitrogen Detection Technologies, Eugene, OR, USA).

### Preparation of *Ehrlichia chaffeensis* vacuoles and latex bead phagolysosomes

Latex bead phagolysosomes were prepared as described previously [12]. DH82 cells were incubated with host cell-free *E. chaffeensis* (multiplicity of infection of 100∶1, bacteria to macrophage) for 90 min to allow for internalization. Non-ingested *E. chaffeensis* were removed by washing with PBS, and the cells were incubated for an additional 3 days. When the infectivity reached to 95%, the infected DH82 cells were removed using a cell scraper. The cells were washed twice with PBS and once with homogenization buffer (250 mM sucrose, 0.5 mM EGTA, 20 mM HEPES/KOH, pH 7.2) containing protease inhibitor cocktail (Sigma-Aldrich, St. Louis, MO, USA). Cells were homogenized on ice using a 10 ml syringe with a 22-G needle (12–14 strokes). Homogenization was carried out until approximately 80% of cells were disrupted without major breakage of nuclei, as monitored by light microscopy. Cell extracts were pelleted by centrifugation at 300×g for 5 min at 4°C. The resulting supernatant contained ECV, which was designated as the post-nuclear supernatant (PNS). The PNS was brought to 39% sucrose by adding 65% sucrose solution. For purification of the ECVs, this 39% sucrose suspension containing *E. chaffeensis* phagosomes (10 ml) was gently layered on top of a sucrose solution consisting of 5 gradients of 10% sucrose (9 ml), 25% sucrose (7 ml), 32.5% sucrose (7 ml), 55% sucrose (3 ml), and 65% sucrose (2 ml). The samples were centrifuged at 100,000×g for 1 h at 4°C. *E. chaffeensis* phagosomes were located between the 55% and 65% sucrose gradients. The 55–65% sucrose fraction was collected, mixed with sucrose-free HB buffer (0.5 mM EGTA, 20 mM HEPES/KOH, pH 7.2) to a final sucrose concentration of 11%, and then was placed on top of 15% Ficoll solution (5% sucrose, 0.5 mM EGTA, 20 mM HEPES/KOH, pH 7.2). The samples were centrifuged at 18,000×g for 30 min at 4°C. The resulting pellets were resuspended in HB buffer containing protease inhibitor cocktail (Sigma-Aldrich, St. Louis, MO, USA), followed by differential centrifugation (300×g, 10 min; 1,000×g, 10 min) to remove nuclei. Smears of the ECV preparations were checked by microscopy after staining with Diff-Quik Kit (Baxter Scientific Products, Obetz, Ohio).

### Mass spectrometry

The phagolysosomes containing latex beads and the ECVs were resuspended in sodium dodecyl sulfate polyacrylamide gel electrophoresis (SDS-PAGE) sample buffer, and then separated by SDS-PAGE (4–12%, Bio-Rad, Hercules, CA, USA). After Coomassie blue staining, gel slices were digested with trypsin, and the resulting peptides were analyzed by nano-high-performance liquid chromatography tandem mass spectrometry (nano-HPLC-MS/MS) in the Protein Core Facility at the University of Texas Medical Branch (Galveston, TX, USA). LC-MS/MS results were searched against data in the International Protein Index (IPI) canine database using the Mascot search algorithm (v2.1, Matrix Science, London, UK). The search parameters were described below. Oxidation was used as variable modification; mass tolerance for peptide was set as 0.01 Da; and mass tolerance for fragment was set as 0.3 Da. The use of trypsin was specified and one missed cleavage was allowed. A cutoff expectation value of 0.01 (significance threshold) was used for MS/MS spectra that resulted in a false discovery rate of 2.40% (automatic decoy database search). Criteria of protein identification were as follows: minimum of two observed significant peptides; single peptide with E values of <0.005.

### Confocal microscopy analysis

The antigen slides were blocked with 1% bovine serum albumin (BSA) for 1 h at room temperature to reduce non-specific staining, and then incubated sequentially with assorted primary antibodies and corresponding secondary antibodies for 1 h at 37°C, respectively. Negative controls consisted of uninfected DH82 cells incubated with primary antibodies and corresponding secondary antibodies and infected DH82 cells incubated with secondary conjugated antibodies alone. The antigen slides were washed in PBS, and incubated with DAPI in PBS for 15 min at room temperature to label host nuclei. LysoSensor Green DND-153 and LysoSensor Green DND-189 (Molecular Probes Invitrogen Detection Technologies, Eugene, OR, USA) were used to indicate the pH inside *E. chaffeensis* morulae according to the manufacturer's instructions. Briefly, LysoSensor probes were mixed with medium to obtain the final working solution. The prewarmed (37°C) LysoSensor probe-containing medium was added to the cells. Uninfected DH82 cells labeled with LysoSensor probes were used as a negative control. After incubation for 1 h at 37°C, the cells were stained with DAPI before examination by confocal microscopy. The slides were examined with a Zeiss LSM 510 UV META laser scanning confocal microscopy (Carl Zeiss Optronics GmbH, Oberkochen, Germany). The images were analyzed using the LSM 5 Image Browser (Carl Zeiss Optronics GmbH).

## Results

### Purification of vacuoles containing *E. chaffeensis* or latex beads

We initially attempted to purify ECVs at different time points post-infection. However, we failed to purify ECVs from DH82 cells at 1 day after *E. chaffeensis* infection and obtained only a small amount of ECVs from host cells at 2 days after infection due to the small size and number of the morulae. A large quantity of ECVs was purified at 3 days after *E. chaffeensis* infection when morulae became larger and infectivity reached to approximately 95%. Therefore, we purified ECVs from DH82 cells at 3 days post-infection.

The fact that the density of latex bead-containing phagolysosomes was less than that of any other cell organelle allowed one-step purification by sucrose density gradient centrifugation. However, the purification of ECVs required multiple steps because their densities are similar to other organelles. We found that the density of nuclei was similar to the larger ECVs. Thus, ECVs were further purified by removing contaminated nuclei with low speed centrifugation. Furthermore, it was convenient to aspirate the interface of 65–55% sucrose and reduce the contamination of cells and intact nuclei by increasing the volume of 65% sucrose from 1 ml to 2 ml.

### Proteomics analysis of ECVs and latex bead phagolysosomes

**Figure 1:**
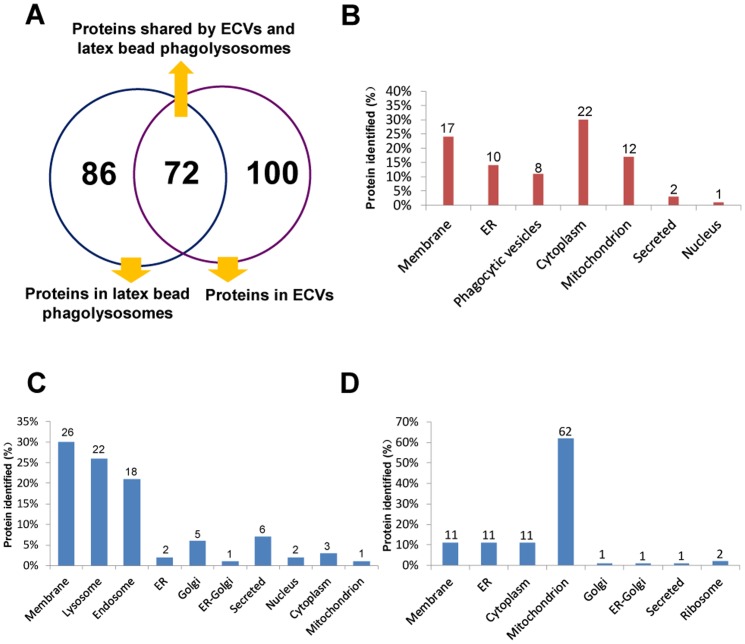
Graphic representation of the proteins identified in ECVs and phagolysosomes containing latex beads. (A): Schematic representation of proteins present in ECVs and latex bead phagolysosomes; (B): Proteins shared by ECVs and latex bead-containing phagolysosomes were grouped according to their cellular locations; (C): Proteins detected in all latex bead-containing phagolysosomes but not detected in any ECVs were classified based on their cellular locations; (D): Proteins present only in ECVs were grouped according to their cellular locations.

The LC-MS/MS experiments were repeated four times. We identified 680 proteins in at least one of the ECV preparations by LC-MS/MS, of which 260 different proteins were found in at least three of the ECV preparations. Of the 260 proteins, 172 were identified in all four ECV preparations. A total of 743 proteins were identified in at least one of the latex bead phagolysosome preparations. We identified 259 proteins in at least three of the latex bead phagolysosomes, of which all but 158 were found in all four of the latex bead phagolysosomes. In addition, 72 proteins were identified four times in both ECVs and latex bead phagolysosomes, demonstrating moderate concordance of proteins within the two compartments (Figure 1A). These 72 proteins were grouped according to their experimentally determined or predicted cellular locations (Figure 1B, Table S1, Table S4). A significant number of proteins were identified as being located in the cytoplasm (30%), membrane (24%), endoplasmic reticulum (ER) (14%), phagocytic vesicles (11%), and mitochondria (17%) in both ECVs and latex bead phagolysosomes. Only a few nuclear proteins (1%) were identified in the two compartments. These proteins have roles in trafficking, phagosome acidification, endocytosis, signaling, or the cytoskeleton (Table S1). Among the proteins shared by phagolysosomes containing latex beads and ECVs were the small GTPases: Rab7A, Rab5C, Rab10 and Rap-1B. Membrane and endoplasmic reticulum (ER) proteins were also identified within the two compartments, including aminopeptidase N, neutral amino acid transporter B (0), calnexin and protein disulfide-isomerase. Calnexin has been previously identified in phagosomes containing latex beads, zymosan, killed bacteria, and *M. tuberculosis* [13], [14], [15], [16], [17]. We identified calnexin in both latex bead phagolysosomes and ECV preparations. Clathrin, which contributes to the uptake of pathogenic bacteria, has been identified previously in the proteome of *Legionella*-containing vacuoles (LCVs) [18]. Another study demonstrated that clathrin was detected in the PNS but was absent from the *M. bovis* BCG phagosomes [19]. We identified clathrin heavy chain 1 within the two compartments. The early endosomal protein, transferrin receptor, and proteins involved in compartmental acidification, including V-type ATPase catalytic subunit A, V-type ATPase subunit d1, V-type ATPase subunit C1, V-type ATPase subunit B, and V-type ATPase subunit E1 were detected in both latex bead phagolysosomes and ECVs. Many proteins that have been identified as major constituents of exosomes were present in ECV preparations, including Rap-1B, annexin A2, actin, elongation factor 1 alpha 1 and 14-3-3. The 14-3-3 family proteins, which have been identified by proteomics in latex bead phagosomes and bacterial phagosomes [13], [19], participate in several signaling pathways and interact with numerous proteins involved in vesicular transport [20]. We detected two members of the 14-3-3 family (ζ/δ and γ) in two of four latex bead phagolysosome preparations and two members of this protein (γ and θ) in two of four ECV preparations.

Although some proteins were present in both ECVs and latex bead phagolysosomes, the unique protein profile for each compartment distinguished ECVs and latex bead phagolysosomes, indicating important differences in their cell biology. The 86 proteins (Figure 1A) present in all four preparations of the latex bead phagolysosomes but undetected in ECVs were grouped according to their putative cellular location (Figure 1C, Table S2, Table S5). A number of membrane proteins (30%), as well as proteins designated as located in lysosomes (26%) and endosomes (21%) were found to be associated with latex bead phagolysosomes. These proteins consisted of (a) proteins involved in endocytic recycling, Rab-11A, ADP-ribosylation factor 6 and myoferlin; (b) plasma membrane proteins flotillin-1; (c) cell membrane proteins, transforming protein RhoA, Rho-related GTP-binding protein RhoG, Ras-related protein Rab-35, and Ras-related protein Rab-13 (d) proteins involved in membrane trafficking, Rab-14, Rab-6A, and Rab-2A; (e) late endosomal and lysosomal proteins, ADP-ribosylation factor (Arf)-like protein 8b/8a, acid ceramidase, cathepsin D, tripeptidyl-peptidase 1, cathepsin S, alpha-N-acetylglucosaminidase, lysosomal acid phosphatase, proactivator polypeptide, regulator complex protein LAMTOR1, and vacuolar fusion protein CCZ1 homolog. The absence of the lysosomal proteins in ECVs suggested that no fusion of the *E. chaffeensis* phagosomes had occurred with lysosomes.

As shown in Figure 1A, 100 proteins were present only in ECVs, but not in latex bead phagolysosomes (Table S3 and Table S6). These proteins were classified based on their putative cellular location (Table S3). ER and mitochondria have been previously recognized to be associated with *Legionella*-containing vacuoles [18], [21]. In our study, many mitochondrial proteins (62%), as well as proteins designated as located in membrane (11%), ER (11%) and cytoplasm (11%) were found to be associated with purified ECVs (Figure 1D). In contrast, only a few proteins designated as located in the Golgi apparatus (1%), ER-Golgi compartment (1%) and ribosomal compartment (2%) were identified in the ECV proteome. Moesin was identified by mass spectrometry in *M. bovis* BCG phagosomes and latex bead phagolysosomes [19]. In this study, we identified this protein in all of the ECV preparations, but did not identify it in any of the latex bead phagolysosome preparations. The function of moesin within the ECVs remains to be determined, but, in view of its previously demonstrated roles [22], [23], it could be a crucial component of the actin assembly machinery and play a role in phagosome acidification. Lipid raft protein erlin-2, which has been identified as an ER-associated raft protein [24], and platelet endothelial cell adhesion molecule that is involved in cell adhesion and signal transduction were detected in all ECV preparations. Although these two proteins have been reported previously in BCG phagosome proteomics studies [19], their roles in phagosome formation and maturation have not been determined.

### Confirmation of LC-MS/MS results by confocal microscopy

**Figure 2:**
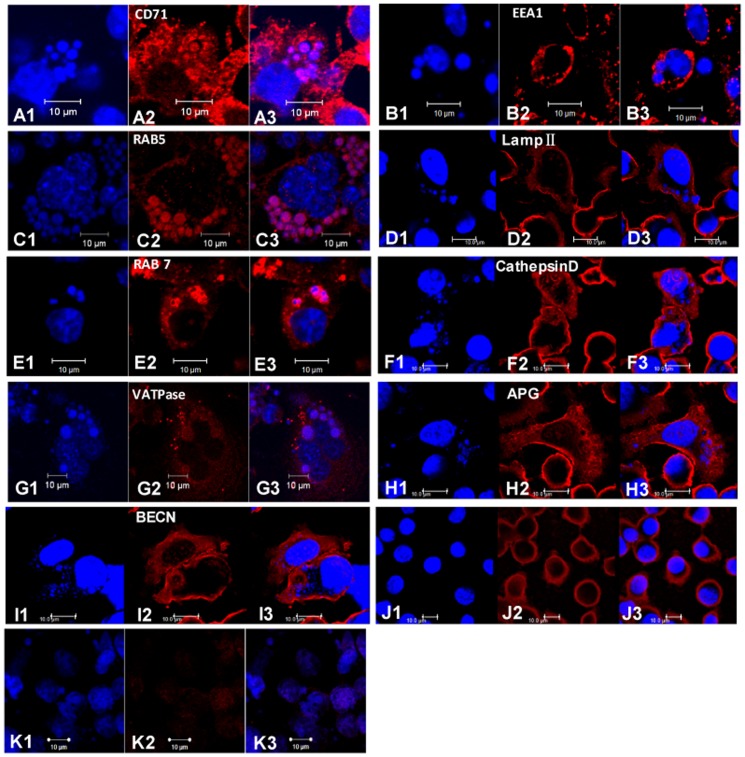
Confocal microscopy images of selected cell markers in infected DH82 cells. When the infectivity reached 95%, the infected cells were fixed and permeabilized. The slides were incubated with the primary antibodies specific to the selected makers (anti-CD71, anti-EEA1, anti-Rab5, anti-LAMP II, anti-Rab7, anti-VATPase, anti-MAP LC3, or anti-BECN antibodies), and then incubated with corresponding secondary conjugated antibodies. In all images, the blue indicated DAPI-stained nuclei (large blue body) or *E. chaffeensis* morulae; the red indicated host cell proteins labeled with antibodies to CD71 (A), EEA1 (B), Rab5 (C), LAMP II (D), Rab 7 (E), cathepsin D (F), VATPase (G), MAP LC3 (H), and BECN (I). Uninfected DH82 cells incubated with primary antibodies and corresponding secondary antibodies (J) and infected DH82 cells incubated with secondary conjugated antibodies alone (K) served as negative controls. In each panel, whether the DAPI staining was co-stained with host cell proteins determined the co-localization of *E. chaffeensis* and the host cell proteins. These results were confirmed in three independent experiments.

Confocal microscopy was used to confirm whether some proteins detected by MS were indeed located on *E. chaffeensis* vacuoles. Confocal microscopy demonstrated that *E. chaffeensis* vacuoles were strongly labeled by antibodies to Rab5, transferrin receptor (CD71), and Rab7, and weakly labeled by vacuolar type H^+^-ATPase, but not labeled by antibodies to EEA1, cathepsin D and LAMP II (Fig. 2). The autophagic markers, MAP LC3 (APG8a) and Beclin-1, were not detected on *E. chaffeensis* vacuoles (Fig. 2). These findings were consistent with the MS results.

### Evaluation of the pH inside *E. chaffeensis* vacuoles by immunofluorescence analysis

**Figure 3:**
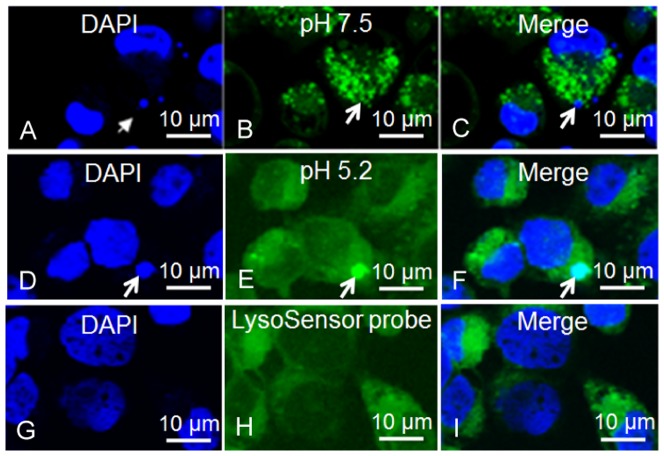
LysoSensor Green DND-189 and DND-153 labeling of *E. chaffeensis*-infected DH82 cells. (A and D): The confocal images containing DAPI-stained nuclei and *E. chaffeensis* morulae (blue); (B): LysoSensor Green DND-153-labeled infected DH82 cells; (C): merged image of A and B; (E): LysoSensor Green DND-189-labeled infected DH82 cells (green); (F): merged image of D and E; (G): DAPI-stained uninfected DH82 cells; (H): LysoSensor probe-labeled uninfected DH82 cells; (I): merged image of G and H. Arrows indicate *E. chaffeensis* morulae. These results were confirmed in three independent labeling experiments.

Confocal microscopy demonstrated that *E. chaffeensis* vacuoles co-localized with LysoSensor Green DND-189 with a p*K*_a_ value of 5.2, but did not co-localize with LysoSensor Green DND-153 with a p*K*_a_ value of 7.5. This result suggested that *E. chaffeensis* vacuoles were acidified with an approximate pH of 5.2 (Fig. 3).

### *Ehrlichia* vacuoles did not fuse with lysosomes nor inhibit heterologous particles to fuse with lysosomes

**Figure 4:**
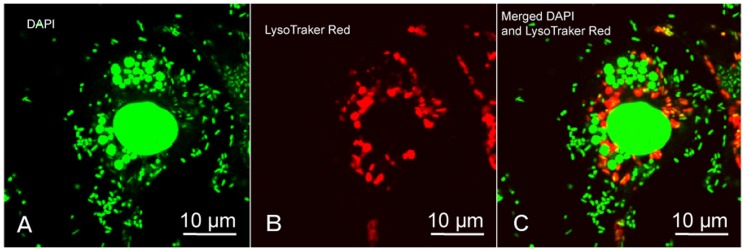
Co-inoculation of DH82 cells with *E. chaffeensis* and formalin-fixed *E. coli*. Three days after *E. chaffeensis* infection, DH82 cells were inoculated with formalin-fixed *E. coli* and stained with DAPI and LysoTracker Red. (A): The confocal images containing DAPI-stained nuclei, *E. chaffeensis* morulae and formalin-fixed *E. coli* (green); (B): LysoTracker Red-stained infected DH82 cells; (C): merged image of A and B. When the DAPI and LysoTracker Red images were merged (C), co-localization was not apparent providing evidence that ECVs did not fuse with the cellular lysosomes. But, *E. coli* in the same cell near the nuclei were co-localized with Lysotracker Red (yellow in the merged panel).

To demonstrate the mechanism by which *E. chaffeensis* inhibits fusion of lysosomes with phagosomes, we inoculated DH82 cells simultaneously with *E. chaffeensis* and formalin-fixed *E. coli*. Confocal microscopy demonstrated that *E. coli* inside DH82 cells near nuclei were co-localized with LysoTracker Red (a red-fluorescent dye for labeling and tracking acidic organelles in live cells, Invitrogen), indicating fusion of *E. coli* phagosomes with lysosomes. Within the same cell, *E. chaffeensis* were located in the cytoplasm, and never co-localized with LysoTracker red (Fig. 4). These results demonstrated that *E. chaffeensis* vacuoles did not fuse with lysosomes, but *E. chaffeensis* infection did not prevent phagosomes containing heterologous particles from fusing with lysosomes.

## Discussion

In the present study, we present a method for purification of *E. chaffeensis* vacuoles by using a combination of sucrose density gradient techniques and differential centrifugation, and then determined the protein constituents of *E. chaffeensis* vacuoles by proteomics methods and compared the composition of *E. chaffeensis* phagosomes with that of the latex bead-containing phagolysosomes. This is the first comprehensive proteomics study of *E. chaffeensis* vacuoles and the first MS-based proteomics study of latex bead phagolysosomes in DH82 cells. Proteins identified in the phagosomes by proteomics were further confirmed by immunofluorescence analysis to interpret the characteristics and functions of *E. chaffeensis* vacuoles.

During phagocytosis, immediately after engulfing a particle, the membrane of the nascent phagosome resembles the plasma membrane [25], [26], and then undergoes a series of fusions with vesicles and eventually matures into a phagolysosome by incorporating lysosomal proteins. Within five minutes, the nascent phagosome selectively fuses with early endosomes to become an early phagosome, which has distinct molecular markers such as Rab5, early endosome antigen 1 (EEA1), transferrin receptor, and VAMP-3. In 10 minutes, the phagosome selectively fuses with late endosomes becoming the late phagosome, which is characterized by the presence of Rab7, Rab9, and/or the mannose-6 phosphate receptor (M6PR). In 20 to 40 minutes, the phagosome fuses with lysosomes and finally matures into a phagolysosome, which contains lysosome-associated membrane proteins (LAMPs) and hydrolytic enzymes such as mature cathepsin D. Small GTPases, particularly Rab5 and Rab7, are required for docking and fusion of the phagosome with endosomes. Rab5 regulates the docking and fusion of the phagosome with early endosomes [27]. Rab7 is required for the fusion of a late phagosome with a lysosome and is generally used as a late endosome marker protein [28], [29], [30], [31]. The pH inside the vacuole is around 6 for early phagosomes, 5.5 for late phagosomes, and 4.5 for phagolysosomes [9]. Our results demonstrated that the *E. chaffeensis* vacuoles were acidified and positive for Rab7, but negative for lysosomal markers, suggesting that *E. chaffeensis* resided in a late endosomal compartment.

Rab5 is found in early endosomes and has been implicated in the maturation of phagosomes containing intracellular pathogens [32]. The presence of Rab5 has been demonstrated on *M. tuberculosis* and *E. chaffeensis* vacuoles [33], [34] by immunoelectron microscopy. In our study, we identified Rab5C in all of the latex bead-containing phagolysosomes and ECVs. Rab5C identification by mass spectrometry on vacuoles was further confirmed by immunofluorescence analysis, which indicated that Rab5C was recruited to *E. chaffeensis* vacuoles.

Since the early endosome serves as a hub of the endocytic pathway, their effectors play an important role in early endosomal trafficking. The Rab5 effector early endosomal membrane-tethering molecule, EEA1, was undetectable in either latex bead-containing phagolysosomes or ECVs by proteomics analysis. Immunofluorescence analysis results also showed that *E. chaffeensis* vacuoles were not labeled by EEA1 antibody. A previous study reported that *E. chaffeensis* inclusions were co-localized with EEA1 [34]. Another study has shown transient EEA1 recruitment to latex bead phagosomes and exclusion from mycobacterial phagosomes [35]. In our study, EEA1 was undetectable in latex bead phagolysosomes most likely because the latex bead phagolysosomes were prepared 3 days post-internalization, not from 1-h post-internalization. Based on a previous observation, the presence of EEA1 on phagosomes is required for the subsequent acquisition of late endocytic characteristics [35]. We could not detect EEA1 on *E. chaffeensis* vacuoles because EEA1 might have been transiently present on *E. chaffeensis* vacuoles and were undetectable at the time points that we collected phagosomes containing *E. chaffeensis*.

Rab7 regulates late endocytic/phagocytic trafficking, and it has been reported to be present on bacterial phagosomes and latex bead phagosomes [13], [36], [37]. We identified Rab7A in all of the latex bead phagolysosomes and *E. chaffeensis* phagosomes from DH82 cells. In addition to Rab7, Rap-1B, which has been identified in late endosomes/lysosomes by immunomicroscopy [38] and latex bead phagosomes by proteomics studies [13], and Rab10, which plays a prominent role in phagolysosome formation and modulating phagosome maturation [39], were also identified by mass spectrometry in *E. chaffeensis* vacuoles. These results suggested that Rab7A, Rap-1B and Rab10 played important roles in modulating *E. chaffeensis* vacuole maturation.

Several proteins that have been identified as major constituents of lysosomes were present in all four latex bead phagolysosomes, but in none of the *E. chaffeensis* phagosomes. *E. chaffeensis* vacuoles were not co-localized with the LysoTracker Red probe, which was useful for selective fluorescent labeling and tracking of acidic organelles such as lysosomes in live cells. The absence of lysosomal proteins in ECVs indicated that fusion between ECVs and lysosomes did not occur. Previous studies demonstrated that ECVs did not contain LAMP I and CD63 [10], [34]. Our study demonstrated that ECVs were negative for LAMP II and lysosomal protease cathepsin D, further supporting the conclusion that the *E. chaffeensis* vacuoles did not fuse with lysosomes.

Rab2, which plays a role in protein transport from the ER to the Golgi apparatus, has been identified by proteomics studies [40], [41] and in latex bead phagosome preparations [13]. It has been suggested that loss of Rab2 delays lysosome fusion [42], but the mechanism by which Rab2 mediates phagosome maturation remains uncertain. We identified this protein in all of the latex bead phagosome preparations, but in none of the *E. chaffeensis* vacuole preparations. It is not clear whether loss of Rab2 plays a role in delaying *E. chaffeensis* phagosome maturation. Thus, its role on phagosomes remains to be elucidated.

Acidification is essential during phagosome maturation and is also a marker of a ‘functional’ phagosome [43]. V-type ATPases function to acidify compartments. V-ATPase D subunit and A1 subunit of the V_0_ ATPase are required for phagosome maturation and fusion of phagosomes with lysosomes [44], [45], respectively. However, how V-type ATPases regulate phagosome maturation is unknown. In our study, V-type ATPase catalytic subunit A, V-type ATPase subunit d1 (D subunit of the V_0_ ATPase),V-type ATPase subunit B, V-type ATPase subunit E1 and V-type ATPase subunit C1 were identified in all latex bead phagolysosomes and *E. chaffeensis* vacuoles by mass spectrometry. Confocal microscopy results demonstrated that the pH inside *E. chaffeensis* vacuoles was low (pH≈5.2) and the *E. chaffeensis* vacuoles reacted with antibodies to H^+^-ATPase. A previous study also demonstrated that *E. chaffeensis* vacuoles were weakly positive for H^+^-ATPase [10]. These results suggested that the *E. chaffeensis* vacuoles were acidified by proton translocation across the phagosomal membrane, which was mediated by vacuolar-type H^+^-ATPase [46].

Mitochondria have been reported to accumulate around the parasitophorous vacuole of intracellular bacteria *Chlamydia psittaci* and *Legionella pneumophila* [47], [48]. Previous studies have investigated the association of *E. chaffeensis* with mitochondria by electron microscopy and immunofluorescence microscopy [49], [50]. Our results demonstrated that ECVs are stably associated with mitochondria even after homogenization.

Autophagy has been identified as the second line of lysosomal defense against intracellular bacteria that breaches the primary lysosomal defense by entering the cytosol [51], [52], [53]. The first evidence of autophagy as a defense system against bacteria comes from a study of *Rickettsia* [52]. MAP-LC3 is associated with autophagosomal membranes, and it is a useful marker for monitoring autophagy. Study has demonstrated that autophagic markers MAP-LC3 and Beclin-1 are co-localized with *Anaplasma phagocytophilum* vacuoles and stimulation of autophagy by rapamycin-favored *A. phagocytophilum* infection [54]. *Ehrlichia* and *Anaplasma* belong to the *Anaplasmataceae* family. Thus, we wanted to know whether *E. chaffeensis* is associated with autophagy. No autophagic markers were detected by mass spectrometry. Furthermore, we confirmed that autophagy did not occur by using MAP-LC3 and Beclin-1 as markers for monitoring autophagy, suggesting that *E. chaffeensis* inhibited its vacuoles to fuse with autophagic vacuoles.

We demonstrated that in DH82 cells containing both *E. chaffeensis* and formalin-fixed *E. coli*, *Ehrlichia* morulae did not fuse with lysosomes, but did not inhibit phagosomes containing formalin-fixed *E. coli* from fusing with lysosomes. Formalin-fixed *E. coli* within the cell did not co-localize with LysoTracker Red most likely because these *E. coli* just entered the cell in phagosomes that had not yet fused with the lysosomes; but *E. coli* surrounding the nuclei had fused with lysosomes and were co-localized with LysoTracker Red. These observations suggested that *Ehrlichia* did not affect the phagosome-lysosome fusion machinery of the host cell and the mechanism responsible for the inhibition of phagosome-lysosome fusion by *E. chaffeensis* is most likely ehrlichial modification of its vacuolar membrane rather than inhibition of expression of proteins involved in membrane trafficking.

A previous study suggested that *Ehrlichia* vacuoles are early endosomes because they selectively accumulate transferrin receptor and lack the lysosomal protein LAMP I [10], [34]. However, this conclusion is premature because the transferrin receptor is not specific for early endosomes and the presence of a late endosomal marker on *Ehrlichia* vacuole such as Rab7 had not been determined. The transferrin receptor is present not only on early phagosomes, but also on late phagosomes and phagolysosomes containing the dead listeriolysin-deficient mutant, *Listeria monocytogenes* [55].

Our study demonstrated that *E. chaffeensis* vacuoles were positive for the early endosomal marker, Rab5, and the late endosomal marker, Rab7. Based on the fact that the *E. chaffeensis* vacuoles were positive for Rab7, but negative for lysosomal proteins, and *E. chaffeensis* vacuoles were acidified, we concluded that *E. chaffeensis* vacuoles were capable of fusing with early endosomes and maturing into late endosomes, but did not fuse with lysosomes. Thus, *E. chaffeensis* vacuoles exhibited arrested maturation despite Rab7 acquisition. This phenomenon has been reported in a study of *Mycobacterium tuberculosis* and *Legionella pneumophila* phagosomes [36]. Further studies indicated that *E. chaffeensis* inhibits its vacuoles from fusing with autophagic vacuoles to escape autophagy and the possible mechanism by which *E. chaffeensis* inhibits fusion of lysosomes with phagosomes is to modify its vacuolar membrane.

## Supporting Information

Table S1 **Proteins shared by** ***Ehrlichia chaffeensis*** **phagosomes and latex bead phagolysosomes.**
(DOCX)

Table S2 **Proteins detected in all of latex bead phagosomes that were not detectable in any ECVs.**
(DOCX)

Table S3 **Proteins detected in ECVs that were not detectable in any latex bead phagolysosomes.**
(DOCX)

Table S4 **Mascot results for proteins shared by** ***Ehrlichia chaffeensis*** **phagosomes and latex bead phagolysosomes.**
(DOCX)

Table S5 **Mascot results for proteins detected in all of latex bead phagosomes that were not detectable in any ECVs.**
(DOCX)

Table S6 **Mascot results for proteins detected in ECVs that were not detectable in any latex bead phagolysosomes.**
(DOCX)
