# Supplementary material for: Proteomic Analysis of the Ehrlichia chaffeensis Phagosome in Cultured DH82 Cells
Source: PLoS One. 2014 Feb 18;9(2):e88461. doi: 10.1371/journal.pone.0088461 (PMC3928192; doi:10.1371/journal.pone.0088461)
Supplement: Table S1 — Proteins shared by Ehrlichia chaffeensis phagosomes and latex bead phagolysosomes. (DOCX) [file pone.0088461.s001.docx]

**Supplemental Table S1: Proteins shared by *Ehrlichia chaffeensis* phagosomes and latex bead phagolysosomes**

| **Protein identified** | **Accession No.** | **Reported location** | **Physiological function** |
| --- | --- | --- | --- |
| **Membrane** |  |  |  |
| Rap-1B | P61223 | Cell membrane | Involved in initiation of oxidative burst in neutrophils |
| Rab10 | Q5R5U1 | Cell membrane | Vesicular traffic and neurotransmitter release |
| Keratin | P04264 | Cell membrane | Regulate the activity of kinases |
| Keratin, type I cytoskeletal 10 | Q6EIZ0 | Cell membrane | Regulate the activity of kinases |
| Keratin, type II cytoskeletal 7 | Q29S21 | Cell membrane | Regulate the activity of kinases |
| Keratin, type II cytoskeletal 1 | Q6EIY9 | Cell membrane | Regulate the activity of kinases |
| Keratin, type II cytoskeletal 79 | Q148H7 | Cell membrane | Regulate the activity of kinases |
| Keratin, type I cytoskeletal 9 | P35527 | Cell membrane | Regulate the activity of kinases |
| Integrin alpha-V | P06756 | Membrane | Receptors for vitronectin |
| Integrin β2 | P11835 | Plasma membrane | Cell surface adhesion glycoprotein |
| Aminopeptidase N | P79143 | Membrane | Aminopeptidase |
| Neutral amino acid transporter B(0) | Q95JC7 | Membrane | Unknown |
| Thioredoxin-related transmembrane protein 1 | Q0Z7W6 | Membrane | Involved in various redox reactions |
| Leucine-rich repeat-containing protein 59 | Q5E9X4 | Microsome membrane | Required for nuclear import of FGF1 |
| Clathrin heavy chain 1 | Q68FD5 | Cytoplasmic vesicle membrane | The major protein of the polyhedral coat of coated pits and vesicles |
| V-type proton ATPase 116 kDa subunit a isoform 1 | Q29466 | Cytoplasmic vesicle membrane | Required for assembly and activity of the vacuolar ATPase |
| CD44 antigen | Q28284 | Plasma membrane | Hyaluronic acid receptor |
| **Endoplasmic reticulum (ER)** |  |  |  |
| Calnexin | P24643 | Endoplasmic reticulum membrane | Chaperone; protein synthesis and folding |
| Thromboxane-A synthase | Q2KIG5 | Endoplasmic reticulum membrane | Enzyme |
| Transmembrane emp24 domain-containing protein 9 | Q3T133 | Endoplasmic reticulum membrane | Involved in vesicular protein trafficking |
| Peptidyl-prolyl cis-trans isomerase B | P80311 | Endoplasmic reticulum lumen | Accelerating the folding of protein |
| Endoplasmin (fragment) | O18750 | Endoplasmic reticulum lumen | Endoplasmic reticulum protein |
| Endoplasmic reticulum resident protein 29 | P81623 | Endoplasmic reticulum lumen | Processing of secretory proteins within the ER |
| Protein disulfide-isomerase A3 | P38657 | Endoplasmic reticulum lumen | Catalyze the rearrangement of –s-s- bonds in proteins |
| Protein disulfide-isomerase | P05307 | Endoplasmic reticulum | Disulfide bond rearrangement |
| Dolichyl-diphosphooligosaccharide-protein glycosyltransferase 48 kDa subunit | A6QPY0 | Endoplasmic reticulum membrane | Essential subunit of the N-oligosaccharyl transferase complex |
| Endoplasmin | P41148 | Endoplasmic reticulum lumen | Processing and transport of secreted proteins |
| **Phagocytic vesicles** |  |  |  |
| Rab5C | P51147 | Early endosome | Regulate early endocytic/phagocytic trafficking |
| Transferrin receptor protein 1 | Q9GLD3 | Early endosome, cell membrane | Receptor for endocytosis |
| Rab7A | P18067 | Late endosome | Regulate late endocytic/phagocytic trafficking |
| V-type ATPase catalytic subunit A | P31404 | Endosome-lysosome | Involved in phagosome acidification |
| V-type ATPase subunit d1 | P61420 | Endosome-lysosome | Involved in phagosome acidification |
| V-type ATPase subunit C1 | P21282 | Endosome-lysosome | Involved in phagosome acidification |
| V-type ATPase subunit B | P31408 | Endosome-lysosome | Involved in phagosome acidification |
| V-type ATPase subunit E 1 | P11019 | Endosome-lysosome | Involved in phagosome acidification |
| **Cytoplasm** |  |  |  |
| Actin, cytoplasmic | Q4L0Y2 | Cytoplasm, Cytoskeleton | Involved in various types of cell motility and are ubiquitously expressed in all eukaryotic cells |
| Actin, cytoplasmic 1 | P29751 | Cytoplasm, Cytoskeleton | Involved in various types of cell motility and are ubiquitously expressed in all eukaryotic cells |
| Vimentin | P48616 | Cytoplasm | Cytoskeleton; intermediate filament |
| Cofilin-2 | Q148F1 | Cytoplasm | Controls actin polymerization |
| Elongation factor 1-alpha 1 | A2Q0Z0 | Cytoplasm | EF-TU |
| Guanine nucleotide-binding protein G(I)/G(S)/G(T) subunit β2 | P11017 | Cytoplasma | Transmembrane signaling |
| 14-3-3^a^ | Q5R651 | Cytoplasm | Involved in exocytosis through actin interaction |
|  | P68253 |  |  |
|  | Q0VC36 |  |  |
| 40S ribosomal protein S10 | Q3T0F4 | Cytoplasm | Component of the 40S ribosomal subunit |
| 40S ribosomal protein S18 | Q3T0R1 | Cytoplasm | Component of the 40S ribosomal subunit |
| Peroxiredoxin-1 | Q6B4U9 | Cytoplasm | Involved in redox regulation of the cell |
| 40S ribosomal protein S15a | Q76I82 | Cytoplasm | Component of the 40S ribosomal subunit |
| 40S ribosomal protein S13 | Q56JX8 | Cytoplasm | Component of the 40S ribosomal subunit |
| 60S acidic ribosomal protein P2 | P42899 | Cytoplasm | Plays an important role in the elongation step of protein synthesis |
| 60S ribosomal protein L18 | Q5E973 | Cytoplasm | Belongs to the ribosomal protein L18a family |
| 60S ribosomal protein L10a (Fragment) | P53027 | Cytoplasm | Component of the 60S ribosomal subunit |
| 60S ribosomal protein L13a | Q3SZ90 | Cytoplasm | Component of the 60S ribosomal subunit |
| 60S ribosomal protein L35 | Q29361 | Cytoplasm | Component of the 60S ribosomal subunit |
| Exosome complex component RRP40 | Q3T0E1 | Cytoplasm | Involved in multitude of cellular RNA processing and degradation events |
| 60S ribosomal protein L13 | Q56JZ1 | Cytoplasm | Component of the 60S ribosomal subunit |
| Ubiquitin-60s ribosomal protein L40 | P63050 | Cytoplasm | Involved in DNA repair, ERAD, cell-cycle regulation, and so on |
| Heat shock cognate 71 kDa protein | A2Q0Z1 | Cytoplasm | Acts as a repressor of transcriptional activation |
| Alpha-actinin-1 | Q3B7N2 | Cytoplasm | Anchor actin to a variety of intracellular structures |
| **Mitochondrion** |  |  |  |
| Voltage-dependent anion-selective channel protein 1 | Q9MZ16 | Mitochondrion, Plasma membrane | Channel for small hydrophilic molecules;present in secretory pathway and plasma membrane |
| Voltage-dependent anion-selective channel protein 2 | Q9MZ15 | Mitochondrion | Channel for small hydrophilic molecules |
| Dihydrolipoyllysine-residue succinyltransferase component of 2-oxoglutarate dehydrogenase complex | P11179 | Mitochondrion | Enzyme |
| Stress-70 protein | Q3ZCH0 | Mitochondrion | Implicated in the control of cell proliferation and cellular aging |
| Malate dehydrogenase | Q32LG3 | Mitochondrion matrix | Enzyme |
| GrpE protein homolog 1 | Q3SZC1 | Mitochondrion matrix | Involved in translocation of transit peptide-containing proteins from the inner membrane into the mitochondrion matrix |
| ADP/ATP translocase 3 | P32007 | Mitochondrion inner membrane | ADP/ATP translocation |
| ATP synthase subunit gamma | P05631 | Mitochondrion inner membrane | Mitochondrial membrane ATP synthase |
| ATP synthase subunit α | P19483 | Mitochondrion inner membrane | Generation of ATP; Ion transport |
| ATP synthase subunit alpha liver isoform | Q29596 | Mitochondrion inner membrane | Mitochondrial membrane ATP synthase |
| Mitochondrial import receptor subunit TOM40 homolog | Q1LZB5 | Mitochondrion outer membrane | Import of protein precursors into mitochondria |
| Mitochondrial fission 1 protein | Q3T0I5 | Mitochondrion outer membrane | Promotes the fragmentation of the mitochondrial network |
| **Secreted** |  |  |  |
| Alpha-1-antiproteinase | P34955 | Secreted | Inhibitor of serine proteases |
| AnnexinA2 | P04272 | Secreted, extracellular space, extracellular matrix, basement membrane | Cross-link PM phospholipids with actin cytoskeleton and be involved in exocytosis |
| **Nucleus** |  |  |  |
| Histone H4 | P62802 | Nucleus | Core component of nucleosome |

The experiments of LC-MS/MS were repeated four times.

^a^14-3-3 ζ/δ(Q5R651) and 14-3-3γ(P68253) were detected in two of four latex bead phagolysosomes, 14-3-3γ(P68253) and 14-3-3θ(Q0VC36) were detected in two of four *E. chaffeensis* phagosomes.
