# Supplementary material for: Proteomic Analysis of the Ehrlichia chaffeensis Phagosome in Cultured DH82 Cells
Source: PLoS One. 2014 Feb 18;9(2):e88461. doi: 10.1371/journal.pone.0088461 (PMC3928192; doi:10.1371/journal.pone.0088461)
Supplement: Table S2 — Proteins detected in all of latex bead phagosomes that were not detectable in any ECVs. (DOCX) [file pone.0088461.s002.docx]

**Supplemental Table S2: Proteins detected in all of latex bead phagosomes that were not detectable in any ECVs**

| **Protein identified** | **Accession No.** | **Reported location** | **Physiological function** | **Detection frequency** |
| --- | --- | --- | --- | --- |
| **Membrane** |  |  |  |  |
| Lactadherin | P79385 | Membrane | Contributes to phagocytic removal of apoptotic cells in many tissues | 4 |
| Flotillin-1 | Q5RBL4 | Plasma membrane, phagosomal membrane | Lipid rafts; clathrin-independent endocytosis; formation of caveolae or caveolae-like vesicles | 4 |
| Myoferlin | Q9NZM1 | Cell membrane | Involved in endocytic recycling | 4 |
| Erythrocyte band 7 integral membrane protein | P27105 | Cell membrane | Regulate cation conductance | 4 |
| Ras-related protein Rab-35 | Q5U316 | Cell membrane | Regulator of a fast recycling pathway back to the plasma membrane | 4 |
| DLA class Ⅰhistocompatibility antigen, A9/A9 alpha chain | P18466 | Membrane | Presentation of foreign antigens | 4 |
| Ras-related protein Rab31 | Q6GQP4 | Cell membrane | Unknown | 4 |
| Ras-related C3 botulinum toxin substrate 1 | Q6RUV5 | Cell membrane | Plasma membrane-associated small GTPase which cycles between active GTP-bound and inactive GDP-bound states | 4 |
| ATP-binding cassette sub-family B member 6 | Q9DC29 | Cell membrane | Heme synthesis | 4 |
| Plasma membrane calcium-transporting ATPase 1 | P11505 | Cell membrane | Catalyzed hydrolysis of ATP | 4 |
| Ras-related protein R-Ras | D3Z8L7 | Cell membrane | Regulated the organization of the actin cytoskeleton | 4 |
| Rho-related GTP-binding protein RhoG | P84095 | Cell membrane | formation of membrane ruffles during macropinocytosis | 4 |
| AP-2 complex subunit alpha-2 | Q0VCK5 | Cell membrane | Protein transport | 4 |
| Receptor-type tyrosine-protein phosphatase C | P06800 | Membrane | Protein tyrosine-protein phosphatase | 4 |
| Lipid phosphate phosphohydrolase 1 | O14494 | Cell membrane | Dephosphorylates exogenous bioactive glycerolipids and sphingolipids | 4 |
| Vacuolar fusion protein MON1 homolog B | Q4R4E4 | Membrane | Rab5 effector | 4 |
| Vesicle-associated membrane protein 3 | Q2KJD2 | Membrane | Involved in vesicular transport from the late endosome to the trans-Golgi network | 4 |
| Secretory carrier-associated membrane protein 3 | Q58DR5 | Membrane | Functions in post-Golgi recycling pathways | 4 |
| Solute carrier family 12 member 9 | Q66HR0 | Cell membrane | Inhibitor of SLC12A1 | 4 |
| Ras-related protein Rab-13 | Q5KTJ6 | Cell membrane | Involved in polarized transport | 4 |
| Platelet glycoprotein 4 | Q07969 | Membrane | Cell adhesion molecule | 4 |
| Stabilin-1 | Q9NY15 | Membrane | Acts as a scavenger receptor for acetylated low density lipoprotein | 4 |
| Transforming protein RhoA | Q5REY6 | Cell membrane | Regulates signal transduction pathway | 4 |
| Intercellular adhesion molecule 1 | P33729 | Membrane | Ligands for the leukocyte adhesion protein integrin alpha-L/beta-2 | 4 |
| Synaptotagmin-like protein 4 | Q8VHQ7 | Membrane | Modulates exocytosis | 4 |
| DnaJ homolog subfamily C membrane 5 | Q9H3Z4 | Membrane | Presynaptic function | 4 |
| **Lysosome** |  |  |  |  |
| Lysosome-associated membrane glycoprotein 2 | P13473 | Lysosome, endosome, PM | Protects lysosomal membrane from autodigestion | 4 |
| Lysosome-associated membrane glycoprotein 1 | P11279 | Lysosome, endosome, PM | Presents carbohydrate ligands to selectins | 4 |
| Acid ceramidase | A5A6P2 | Lysosome | Hydrolyzes ceramide into sphingosine and free fatty acid | 4 |
| Tripeptidyl-peptidase 1 | Q9XSB8 | Lysosome | Act as a non-specific lysosomal peptidase | 4 |
| Cathepsin D | Q4LAL9 | Lysosome | Protein degradation | 4 |
| Cathepsin S | Q8HY81 | Lysosome | Key protease responsible for the removal of the invariant chain from MHC classⅡ molecules | 4 |
| Cathepsin Z | Q9UBR2 | Lysosome | Carboxydipeptidase | 4 |
| Alpha-N-acetylglucosaminidase | P54802 | Lysosome | Degradation of heparan sulfate | 4 |
| Lysosomal acid phosphatase | Q0P5F0 | Lysosome | Phosphate monoester hydrolysis | 4 |
| Cathepsin-K | Q3ZKN1 | Lysosome | Osteoclastic bone resorption | 4 |
| N-acetylglucosamine-6-salfatase | Q1LZH9 | Lysosome | Heparan sulfate/keratan sulfate hydrolysis | 4 |
| Vacuolar fusion protein CCZ1 homolog | Q0VD30 | Lysosome membrane | Unknown | 4 |
| Proactivator polypeptide | P07602 | Lysosome | Degradation protein | 4 |
| Cathepsin B | A1E295 | Lysosome | Degradation protein | 4 |
| Ceroid-lipofuscinosis neuronal protein 5 | Q5JZQ9 | Lysosome | Unknown | 4 |
| Vacuolar protein sorting-associated protein 4B | Q0VD48 | Lysosome | Endosomal multivesicular bodies pathway | 4 |
| Tissue alpha-L-fucosidase | Q2KIM0 | Lysosome | Hydrolysis protein | 4 |
| Palmitoyl-protein thioesterase 1 | Q8HXW6 | Lysosome | Protein degradation | 4 |
| Legumain | Q4R4T8 | Lysosome | Hydrolysis of asparaginyl bonds | 4 |
| Beta-glucuronidase | O18835 | Lysosome | Degradation of dermatan and keratan sulfates | 4 |
| N-acetylgalactosamine-6-sulfatase | Q32KH5 | Lysosome | Hydrolysis protein | 4 |
| Dipeptidyl peptidase 2 | Q9EPB1 | Lysosome | Degradation of some oligopeptides | 4 |
| **Endosome** |  |  |  |  |
| Ras-related protein Rab-21 | P55745 | Endosome | Control traffic of β1 integrins | 4 |
| ADP-ribosylation factor 6 | Q007T5 | Endosome membrane, Golgi apparatus | Regulate endocytic recycling and cytoskeleton remodeling | 4 |
| Ras-related protein Rap-2a | Q06AU2 | Recycling endosome | Regulate cytoskeletal rearrangements, cell migration, cell adhesion and cell spreadin | 4 |
| Ras-related protein Rab-11A | P62490 | Recycling endosome | Regulates endocytic recycling | 4 |
| Ras-related protein Rab-8A | P61007 | Recycling endosome | Involved in vesicular trafficking | 4 |
| Ras-related protein Rab-8B | Q5REC9 | Recycling endosome | Involved in vesicular trafficking | 4 |
| Ras-related protein Rab-14 | Q5R8Z8 | Early endosome membrane | Membrane trafficking between the Golgi and endosome | 4 |
| Vesicle-associated membrane protein 7 | Q5RF94 | Late endosome, cytoplasmic vesicle, lysosome | Target or/and fusion of transport vesicles to their target membrane | 4 |
| Ragulator complex protein LAMTOR3 | Q5R3Z6 | Late endosome membrane | Regulate TOR pathway | 4 |
| ADP-ribosylation factor-like protein 8A | Q8VEH3 | Late endosome, lysosome membrane | Endosome and lysosome motility | 4 |
| ADP-ribosylation factor-like protein 8B | Q2KI07 | Late endosome, lysosome membrane | Endosome and lysosome motility | 4 |
| Niemann-pick C1 protein | O15118 | Late endosome membrane | Trafficking of cholesterol | 4 |
| Ras-related protein Rab-27A | Q1HE58 | Late endosome, lysosome | Cytotoxic granule exocytosis | 4 |
| Ragulator complex protein LAMTOR1 | Q3T0D8 | Late endosome, lysosome | Cholesterol homeostasis regulating LDL uptake and Cholesterol release from late endosome/lysosome | 4 |
| CD63 antigen | Q76B49 | Late endosome, lysosome | May regulate transport of proteins | 4 |
| Syntaxin-7 | O70439 | Early endosome membrane | Protein trafficking from plasma membrane to the early endosome | 4 |
| Charged multivesicular body protein 4b | Q9D8B3 | Late endosome membrane | Probable core component of the endosomal sorting | 4 |
| MLN64 N-terminal domain homolog | Q9DCI3 | Late endosome membrane | Interacts with STARD3 | 4 |
| **Endoplasmic reticulum (ER)** |  |  |  |  |
| Calreticulin | P28491 | Endoplasmic reticulum lumen | Promote folding, oligomeric assembly and quality control in the ER | 4 |
| Cytoskeleton-associated protein 4 | Q8BMK4 | Endoplasmic reticulum membrane | Mediates the anchoring of the endoplasmic reticulum to microtubules | 4 |
| **Golgi apparatus** |  |  |  |  |
| Rab-6A | Q5RAV6 | Golgi apparatus membrane | Regulate traffic within Golgi stacks and between ER and Golgi | 4 |
| Rab-6B | A6QR46 | Golgi apparatus membrane | Regulate traffic within Golgi stacks and between ER and Golgi | 4 |
| Nicastrin | Q92542 | Golgi apparatus, ER | Component of the gamma-secretase complex | 4 |
| ADP-ribosylation factor 4 | Q3SZF2 | Golgi apparatus | Protein trafficking | 4 |
| Golgi-associated plant pathogenesis-related protein 1 | Q9CYL5 | Golgi apparatus membrane | Interacts with CAV1 | 4 |
| **Endoplasmic reticulum-Golgi** |  |  |  |  |
| Ras-related protein Rab-2A | Q4R4X6 | Endoplasmic reticulum-Golgi intermediate compartment membrane | Required for protein transport from the endoplasmic reticulum to the Golgi complex | 4 |
| **Secreted** |  |  |  |  |
| Complement C3 | Q2UVX4 | Secreted | Activation of the complement system | 4 |
| Apolipoprotein E | Q03247 | Secreted | Cholesterol transport and catabolism | 4 |
| CD166 antigen | O46634 | Secreted | Cell adhesion | 4 |
| Alpha-2-macroglobulin | Q7SIH1 | Secreted | Inhibit proteinases | 4 |
| Factor XⅡa inhibitor | P50448 | Secreted | Regulate physiological pathways | 4 |
| Sulfated glycoprotein 1 | P10960 | Secreted | Unknown | 4 |
| **Nucleus** |  |  |  |  |
| Histone H2B type 3-B | Q8CGP0 | Nucleus | Core component of nucleosome | 4 |
| Core histone macro-H2A.1 | O75367 | Nucleus | transcription regulation, DNA repair | 4 |
| **Cytoplasm** |  |  |  |  |
| Serpin A3-2 | A2I7M9 | Cytoplasmic vesicle | Serine protease inhibitor | 4 |
| Plectin | Q15149 | Cytoplasm | Interlinks intermediate filaments with microtubules and microfilaments | 4 |
| Synaptic vesicle membrane protein VAT-1 homolog | Q99536 | Cytoplasm | Possesses ATPase activity | 4 |
| **Mitochondrion** |  |  |  |  |
| Prohibitin-2 | Q5RB19 | Mitochondrion inner membrane | Acts as a mediator of transcriptional repression | 4 |

The experiments of LC-MS/MS were repeated four times. Detection frequency indicates the number of latex bead phagosome preparations (out of four total) in which the indicated protein was identified.
