# Supplementary material for: Proteomic Analysis of the Ehrlichia chaffeensis Phagosome in Cultured DH82 Cells
Source: PLoS One. 2014 Feb 18;9(2):e88461. doi: 10.1371/journal.pone.0088461 (PMC3928192; doi:10.1371/journal.pone.0088461)
Supplement: Table S3 — Proteins detected in ECVs that were not detectable in any latex bead phagolysosomes. (DOCX) [file pone.0088461.s003.docx]

**Supplemental Table S3: Proteins detected in ECVs that were not detectable in any latex bead phagolysosomes**

| **Protein identified** | **Accession No.** | **Reported location** | **Physiological function** |
| --- | --- | --- | --- |
| **Membrane** |  |  |  |
| Moesin | Q2HJ49 | Cell membrane; Peripheral membrane protein | Connection of membrane to cytoskeleton |
| Ras-related protein Ral-B | P11234 | Membrane | Signal transduction; regulation of exocytosis |
| Regulator of G-protein signaling 20 | P79348 | Membrane | Inhibit signal transduction |
| Solute carrier family 15 member 2 | P46029 | Membrane | Unknown |
| Platelet endothelial cell adhesion molecule | Q95242 | Plasma membrane | Cell adhesion; signal transduction |
| Adenylate cyclase type 10 | Q866F4 | Cell membrane | Production of the cAMP |
| EH domain-containing protein 1 | Q5E9R3 | Cell membrane, early endosome membrane | Involved in early endocytic membrane fusion and membrane trafficking of recycling endosomes |
| Signal peptidase complex subunit 3 | Q3SZU5 | Menbrane | Component of microsomal signal peptidase complex |
| Major facilitator superfamily domain-containing protein 10 | Q0P5M9 | Membrane | Cellular resistance to apoptosis |
| Stomatin-like protein 2 | Q32LL2 | Membrane | Interact with CACNA2D2 |
| Cleft lip and palate transmembrane protein 1 | Q2NL17 | Membrane | T-cell development |
| **Endoplasmic reticulum (ER)** |  |  |  |
| Erlin-2 | Q1RMU4 | Endoplasmic reticulum membrane; Single-pass type II membrane protein | Lipid raft-associated |
| Kinectin | O97961 | Endoplasmic reticulum membrane; Single-pass type II membrane protein | Involved in Kinesin-driven vesicle motility |
| ERO1-like protein alpha | A5PJN2 | ER membrane | Oxidize proteins to produce disulfide bonds |
| Dolichyl-diphosphooligosaccharide-protein glycosyltransferase subunit 2 | Q3SZI6 | ER membrane | Essential subunit of N-oligosaccharyl transferase enzyme |
| Dolichyl-diphosphooligosaccharide-protein glycosyltransferase subunit 1 | Q9GMB0 | ER membrane | Essential subunit of N-oligosaccharyl transferase enzyme |
| Dolichyl-diphosphooligosaccharide-protein glycosyltransferase subunit STT3A | Q2KJI2 | ER membrane | Essential subunit of N-oligosaccharyl transferase enzyme |
| Neutral alpha-glucosidase AB | P79403 | ER | Metabolism |
| Dolichol kinase | Q58CR4 | ER | Involved in synthesis of the sugar donor Dol-P-Man |
| DnaJ homolog subfamily B member 11 | P81999 | ER | Co-chaperone for HSPA5 |
| Sarcoplasmic/endoplasmic reticulum calcium ATPase | O46674 | ER membrane | Catalysis of hydrolysis of ATP |
| Signal recognition particle receptor subunit alpha | Q3MHE8 | ER membrane | Component of the SRP receptor |
| **Cytoplasm** |  |  |  |
| Tubulin beta-5 chain | Q2KJD0 | Cytoplasm | Cytoskeleton |
| 40s ribosomal protein S9 | A6QLG5 | Cytoplasm | Protein synthesis |
| Myosin light polypeptide 6 | P60661 | Cytoplasm | Cytoskeleton |
| Cytoplasmic dynein 1 intermediate chain 2 | Q0III3 | Cytoplasm | Involved in linking dynein to cargos and to adapter proteins |
| Calcium-binding protein p22 | Q3SYS6 | Cytoplasm | Membrane traffic |
| Transitional endoplasmic reticulum ATPase | Q3ZBT1 | Cytoplasm | Fragmentation of Golgi stacks, formation of the tER |
| Tudor domain-containing protein 3 | Q2HJG4 | Cytoplasm | Recognize and bind dimethylarginine-containing proteins |
| Testin | Q2QLB2 | Cytoplasm | Cell adhesion, cell spreading |
| Septin-2 | Q2NKY7 | Cytoplasm | Required for normal organization of the actin cytoskeleton |
| tRNA-splicing ligase RtcB homolog | Q5E9T9 | Cytoplasm | Catalytic subunit of the tRNA-splicing ligase complex |
| Pyrroline-5-carboxylate reductase 2 | Q17QJ7 | Cytoplasm | Catalysis of the last step in proline biosynthesis |
| **Mitochondrion** |  |  |  |
| Very long-chain specific acyl-CoA dehydrogenase | P48818 | Mitochondrial | Fatty acid β-oxidation system |
| Isocitrate dehydrogenase | P33198 | Mitochondrial | metabolism and energy production |
| NADH-ubiquinone oxidoreductase 75kDa subunit | P15690 | Mitochondrial | Metabolism |
| Succinyl-CoA ligase subunit beta | Q3MHX5 | Mitochondrial | Metabolism |
| Single-stranded DNA-binding protein | Q95KK4 | Mitochondrial | Mitochondrial DNA replication |
| Thioredoxin-dependent peroxide reductase | P35705 | Mitochondrial | Redox regulation |
| Pyruvate carboxylase | Q29RK2 | Mitochondrial | ATP-dependent carboxylation |
| Pyruvate dehydrogenase E1 component subunit beta | P11966 | Mitochondrial | Catalyzes the conversion of pyruvate to acetyl-CoA |
| AFG3-like protein 2 | Q2KJI7 | Mitochondrial | ATP-dependent protease |
| Serine beta-lactamase-like protein LACTB | P83095 | Mitochondrial | Unknown |
| Aconitate hydratase | P16276 | Mitochondrial | Catalyzes the isomerization |
| Aldehyde dehydrogenase | P20000 | Mitochondrial | Metabolism |
| Medium-chain specific acyl-CoA dehydrogenase | Q3SZB4 | Mitochondrial | Lipid metabolism |
| NADH dehydrogenase flavoprotein 2 | P04394 | Mitochondrial | Catalysis |
| Propionyl-CoA carboxylase beta chain | Q2TBR0 | Mitochondrial | Metabolism |
| Acyl-coenzyme A thioesterase 9 | Q3SWX2 | Mitochondrial | Catalyzes the hydrolysis of acyl-CoAs |
| Cytochrome c oxidase subunit 2 | Q2Y0B9 | Mitochondrial | Component of respiratory chain |
| Citrate synthase | Q29RK1 | Mitochondrial | Metabolism |
| Fumarate hydratase | P10173 | Mitochondrial | Metabolism |
| Mitochondrial 2-oxoglutarate/malate carrier protein | P22292 | Mitochondrial | Catalyzes the transport of 2-oxoglutarate |
| 10 kDa heat shock protein | P61603 | Mitochondrial | Protein biogenesis |
| 60 kDa heat shock protein | P31081 | Mitochondrial | Involved in mitochondrial protein import and macromolecular assembly |
| 28S ribosomal protein S22 | P82649 | Mitochondrial | Metabolism |
| ATP synthase subunit O | Q2EN81 | Mitochondrial | Production of ATP |
| Mitochondrial carrier homolog 2 | Q9N285 | Mitochondrial | Induction of mitochondrial depolarization |
| Hexokinase-1 | P27595 | Mitochondrial | Metabolism |
| Glutamate dehydrogenase 1 | P00366 | Mitochondrial | Involved in learning and memory reactions |
| Succinate dehydrogenase flavoprotein subunit | P31039 | Mitochondrial | Involved in transferring electrons |
| Serine hydroxymethyltransferase | Q3SZ20 | Mitochondrial | Mitochondrial thymidylate biosynthesis |
| Acetyl-CoA acetyltransferase | Q29RZ0 | Mitochondrial | Ketone body metabolism |
| NADP transhydrogenase | P11024 | Mitochondrial | Transhydrogenation |
| Sideroflexin-3 | A6QP55 | Mitochondrial | Unknown |
| MOSC domain-containing protein 2 | Q1LZH1 | Mitochondrial | Component of the benzamidoxime prodrug-converting complex |
| Dihydrolipoyl dehydrogenase | P09623 | Mitochondrial | Involved in hyperactivation of spermatazoa |
| Mitochondrial Rho GTPase 1 | Q2HJF8 | Mitochondrial | Mitochondrial trafficking |
| Tricarboxylate transport protein | P79110 | Mitochondrial | Involved in citrate-H(+)/malate exchange |
| LETM1 and EF-hand domain-containing protein 1 | Q0VCA3 | Mitochondrial | Maintenance of mitochondrial tubular networks |
| Cytochrome c1 | P00125 | Mitochondrial | Component of the cytochrome b-c1 complex |
| Pyruvate dehydrogenase protein X component | P22439 | Mitochondrial | Anchoring dihydrolipoamide dehydrogenase (E3) to the dihydrolipoamide transacetylase (E2) core of the pyruvate dehydrogenase complexes of eukaryotes |
| Methylmalonate-semialdehyde dehydrogenase | Q07536 | Mitochondrial | Valine and pyrimidine metabolism |
| Electron transfer flavoprotein subunit alpha | Q2KJE4 | Mitochondrial | Transferring electrons |
| SRA stem-loop-interacting RNA-binding protein | Q32P59 | Mitochondrial | Nuclear receptor corepressor |
| Peptidyl-tRNA hydrolase 2 | Q3ZBL5 | Mitochondrial | Hydrolysis |
| Sideroflexin-1 | B2LU20 | Mitochondrial | Unknown |
| Pentatricopeptide repeat-containing protein 3 | Q2KI62 | Mitochondrial | Unknown |
| Cytochrome c oxidase subunit 4 isoform 1 | Q9TTT8 | Mitochondrial | Component of cytochrome c oxidase |
| Sarcoplasmic/endoplasmic reticulum calcium ATPase 2 | Q00779 | Mitochondrial | Catalyzes the hydrolysis of ATP |
| Lon protease homolog | Q59HJ6 | Mitochondrial | Mediate the degradation of polypeptides |
| Lipoamide acyltransferase component of branched-chain alpha-keto acid dehydrogenase complex | P11181 | Mitochondrial | Conversion of alpha-keto acids to acyl-CoA and CO(2) |
| 2-oxoglutarate dehydrogenase | Q148N0 | Mitochondrial | Conversion of 2-oxoglutarate to succinyl-CoA and CO(2) |
| Sorting and assembly machinery component 50 homolog | Q2HJ55 | Mitochondrial | Required for the assembly pathway of mitochondrial outer membrane proteins |
| ATP synthase subunit d | P13620 | Mitochondrial | Production of ATP |
| ATP synthase subunit e | Q9MYT8 | Mitochondrial | Production of ATP |
| 28S ribosomal protein S23 | Q2NL27 | Mitochondrial | Component of mitochondrial ribosome small subunit 28S |
| NADH dehydrogenase 1 alpha subcomplex subunit 4 | Q01321 | Mitochondrial | Involved in transferring electrons |
| Succinate dehydrogenase iron-sulfur subunit | Q3T189 | Mitochondrial | Involved in transferring electrons |
| 39S ribosomal protein L43 | Q95KE5 | Mitochondrial | Component of mitochondrial ribosome small subunit 39S |
| 28S ribosomal protein S28 | P82928 | Mitochondrial | Component of mitochondrial ribosome small subunit 28S |
| Up-regulated during skeletal muscle growth protein5 | Q3ZBI7 | Mitochondrial | Maintaining the ATP synthase population in mitochondria |
| 4-aminobutyrate aminotransferase | Q9BGI0 | Mitochondrial | Catalysis |
| NADH-ubiquinone oxidoreductase chain 2 | Q330A7 | Mitochondrial | Catalysis |
| 3-hydroxyacyl-CoA dehydrogenase type-2 | O02691 | Mitochondrial | Mitochondrial tRNA maturation |
| **Golgi** |  |  |  |
| AP-1 complex subunit beta-1 | Q08DS7 | Golgi apparatus | Involved in protein sorting in the late-Golgi/trans-Golgi network and/or endosome |
| **ER-Golgi** |  |  |  |
| Vacuole membrane protein 1 | Q0VCK9 | ER-Golgi intermediate compartment membrane | Promote the formation of intracellular vacuoles |
| **Secreted** |  |  |  |
| Bactericidal permeability-increasing protein | P17453 | Secreted | Cytotoxic action |
| **Ribosome** |  |  |  |
| 60S ribosomal protein L24 | Q66WF5 | Ribosome | Component of mitochondrial ribosome small subunit 60S |
| 60S ribosomal protein L7a | Q2TBQ5 | Ribosome | Component of mitochondrial ribosome small subunit 60S |
