# Supplementary material for: Proteomic Analysis of the Ehrlichia chaffeensis Phagosome in Cultured DH82 Cells
Source: PLoS One. 2014 Feb 18;9(2):e88461. doi: 10.1371/journal.pone.0088461 (PMC3928192; doi:10.1371/journal.pone.0088461)
Supplement: Table S4 — Mascot results for proteins shared by Ehrlichia chaffeensis phagosomes and latex bead phagolysosomes. (DOCX) [file pone.0088461.s004.docx]

**Supplemental Table S4: Mascot results for proteins shared by *Ehrlichia chaffeensis* phagosomes and latex bead phagolysosomes**

| **Protein identified** | **Accession No.** | **Protein score** | **Mascot Results** | | | | | | | | | | |
| --- | --- | --- | --- | --- | --- | --- | --- | --- | --- | --- | --- | --- | --- |
| Rap-1B | P61223 | 168^a^ | **Observed** | **Mr(expt)** | **Mr(calc)** | **Score** | | | | **Exp Value** | | **Peptide** | |
|  |  |  | 493.3074 | 984.6002 | 984.5968 | 46 | | | | 0.00048 | | K.LVVLGSGGVGK.S | |
|  |  |  | 629.7857 | 1257.5568 | 1257.5513 | 49 | | | | 0.00023 | | K.YDPTIEDSYR.K | |
|  |  |  | 747.9048 | 1493.7950 | 1493.7878 | 65 | | | | 7.5e-006 | | K.INVNEIFYDLVR.Q | |
|  |  |  | 548.6290 | 1642.8651 | 1642.8600 | 28 | | | | 0.032 | | R.VKDTDDVPMILVGNK.C | |
|  |  |  | 830.4379 | 1658.8612 | 1658.8549 | 71 | | | | 1.5e-006 | | R.VKDTDDVPMILVGNK.C | |
|  |  |  | 555.9806 | 1664.9199 | 1664.9138 | 52 | | | | 7.8e-005 | | K.SALTVQFVQGIFVEK.Y | |
| Rap-1B | P61223 | 104^b^ | **Observed** | **Mr(expt)** | **Mr(calc)** | | **Score** | | | **Exp Value** | | **Peptide** | |
|  |  |  | 493.3110 | 984.6074 | 984.5968 | | 45 | | | 0.00023 | | K.LVVLGSGGVGK.S | |
|  |  |  | 629.7900 | 1257.5655 | 1257.5513 | | 34 | | | 0.0023 | | K.YDPTIEDSYR.K | |
|  |  |  | 747.9099 | 1493.8052 | 1493.7878 | | 78 | | | 1.3e-007 | | K.INVNEIFYDLVR.Q | |
| Rab10 | Q5R5U1 | 81^a^ | **Observed** | **Mr(expt)** | **Mr(calc)** | | **Score** | | | **Exp Value** | | **Peptide** | |
|  |  |  | 631.3637 | 1260.7129 | 1260.7078 | | 55 | | | 6.2e-005 | | K.AFLTLAEDILR.K | |
|  |  |  | 658.8356 | 1315.6567 | 1315.6521 | | 59 | | | 5.1e-005 | | K.LQIWDTAGQER.F | |
| Rab10 | Q5R5U1 | 125^b^ | **Observed** | **Mr(expt)** | **Mr(calc)** | | **Score** | | | **Exp Value** | | **Peptide** | |
|  |  |  | 412.7127 | 823.4107 | 823.4076 | | 26 | | | 0.12 | | K.SFENISK.W | |
|  |  |  | 415.2099 | 828.4052 | 828.4018 | | 28 | | | 0.066 | | R.FFETSAK.A | |
|  |  |  | 536.3264 | 1070.6382 | 1070.6336 | | 57 | | | 4.3e-005 | | K.LLLIGDSGVGK.T | |
|  |  |  | 631.3636 | 1260.7127 | 1260.7078 | | 65 | | | 6.9e-006 | | K.AFLTLAEDILR.K | |
|  |  |  | 658.8359 | 1315.6572 | 1315.6521 | | 52 | | | 0.00023 | | K.LQIWDTAGQER.F | |
|  |  |  | 480.8853 | 1439.6340 | 1439.6277 | | 24 | | | 0.056 | | R.NIDEHANEDVER.M | |
| Keratin | P04264 | 398^a^ | **Observed** | **Mr(expt)** | **Mr(calc)** | | | | **Score** | **Exp Value** | | **Peptide** | |
|  |  |  | 563.2722 | 1124.5297 | 1124.5349 | | | | 45 | 0.00098 | | K.AEAESLYQSK.Y | |
|  |  |  | 590.3073 | 1178.6000 | 1178.5931 | | | | 73 | 2.4e-006 | | K.YEELQITAGR.H | |
|  |  |  | 633.3254 | 1264.6363 | 1264.6299 | | | | 37 | 0.0073 | | R.TNAENEFVTIK.K | |
|  |  |  | 639.3623 | 1276.7101 | 1276.7027 | | | | 53 | 0.00013 | | K.LALDLEIATYR.T | |
|  |  |  | 651.8642 | 1301.7138 | 1301.7078 | | | | 80 | 2.9e-007 | | R.SLDLDSIIAEVK.A | |
|  |  |  | 658.7679 | 1315.5212 | 1315.5173 | | | | 36 | 0.00084 | | K.NMQDMVEDYR.N | |
|  |  |  | 670.8404 | 1339.6662 | 1339.6619 | | | | 65 | 9.5e-006 | | K.SKAEAESLYQSK.Y | |
|  |  |  | 679.3543 | 1356.6940 | 1356.6885 | | | | 65 | 1.1e-005 | | K.LNDLEDALQQAK.E | |
|  |  |  | 692.3522 | 1382.6898 | 1382.6830 | | | | 67 | 5.6e-006 | | K.SLNNQFASFIDK.V | |
|  |  |  | 738.3982 | 1474.7819 | 1474.7780 | | | | 72 | 1.8e-006 | | R.FLEQQNQVLQTK.W | |
|  |  |  | 858.9315 | 1715.8483 | 1715.8438 | | | | 90 | 2.5e-008 | | K.QISNLQQSISDAEQR.G | |
|  |  |  | 665.3333 | 1992.9780 | 1992.9693 | | | | 53 | 8.6e-005 | | R.THNLEPYFESFINNLR.R | |
|  |  |  | 1136.5590 | 4542.2069 | 4542.1894 | | | | 33 | 0.0013 | | K.LDNLQQEIDFLTALYQAELSQMQTQISETNVILSMDNNR.S | |
| Keratin | P04264 | 184^b^ | **Observed** | **Mr(expt)** | **Mr(calc)** | | | **Score** | | **Exp Value** | | **Peptide** | |
|  |  |  | 563.2769 | 1124.5392 | 1124.5349 | | | 50 | | 0.00039 | | K.AEAESLYQSK.Y | |
|  |  |  | 590.3083 | 1178.6020 | 1178.5931 | | | 50 | | 0.00048 | | K.YEELQITAGR.H | |
|  |  |  | 639.3614 | 1276.7083 | 1276.7027 | | | 49 | | 0.00032 | | K.LALDLEIATYR.T | |
|  |  |  | 651.8632 | 1301.7119 | 1301.7078 | | | 74 | | 1.4e-006 | | R.SLDLDSIIAEVK.A | |
|  |  |  | 692.3514 | 1382.6882 | 1382.6830 | | | 56 | | 8.1e-005 | | K.SLNNQFASFIDK.V | |
|  |  |  | 465.2506 | 1392.7300 | 1392.7249 | | | 31 | | 0.025 | | R.TNAENEFVTIKK.D | |
|  |  |  | 738.3990 | 1474.7834 | 1474.7780 | | | 71 | | 2.7e-006 | | R.FLEQQNQVLQTK.W | |
| Keratin, type I cytoskeletal 10 | Q6EIZ0 | 84^a^ | **Observed** | **Mr(expt)** | **Mr(calc)** | | | **Score** | | | **Exp Value** | | **Peptide** |
|  |  |  | 516.3044 | 1030.5942 | 1030.5910 | | | 46 | | | 0.00069 | | R.VLDELTLTK.A |
|  |  |  | 545.7709 | 1089.5272 | 1089.5237 | | | 39 | | | 0.0054 | | K.VTMQNLNDR.L |
|  |  |  | 691.3318 | 1380.6490 | 1380.6408 | | | 66 | | | 6.3e-006 | | R.ALEESNYELEGK.I |
| Keratin, type I cytoskeletal 10 | Q6EIZ0 | 282^b^ | **Observed** | **Mr(expt)** | **Mr(calc)** | | | **Score** | | | **Exp Value** | | **Peptide** |
|  |  |  | 497.2593 | 992.5041 | 992.4927 | | | 30 | | | 0.017 | | K.YENEVALR.Q |
|  |  |  | 545.7754 | 1089.5363 | 1089.5237 | | | 51 | | | 0.00013 | | K.VTMQNLNDR.L |
|  |  |  | 555.2549 | 1108.4953 | 1108.4825 | | | 36 | | | 0.0019 | | K.DAEAWFNEK.S |
|  |  |  | 583.3102 | 1164.6058 | 1164.5775 | | | 33 | | | 0.0069 | | R.LENEIQTYR.S |
|  |  |  | 617.8502 | 1233.6859 | 1233.6717 | | | 36 | | | 0.0022 | | R.LKYENEVALR.Q |
|  |  |  | 691.3356 | 1380.6567 | 1380.6408 | | | 80 | | | 9.4e-008 | | R.ALEESNYELEGK.I |
|  |  |  | 854.3996 | 1706.7846 | 1706.7649 | | | 116 | | | 1.5e-011 | | K.GSIGGGFSSGGFSGGSFSR.G |
|  |  |  | 1056.5375 | 2111.0604 | 2111.0343 | | | 72 | | | 3e-007 | | K.ADLEMQIESLTEELAYLK.K |
|  |  |  | 747.3941 | 2239.1606 | 2239.1293 | | | 29 | | | 0.005 | | K.ADLEMQIESLTEELAYLKK.N |
|  |  |  | 789.7678 | 2366.2817 | 2366.2553 | | | 52 | | | 1.5e-005 | | K.NQILNLTTDNANILLQIDNAR.L |
| Keratin, type II cytoskeletal 7 | Q29S21 | 98^a^ | **Observed** | **Mr(expt)** | **Mr(calc)** | | | **Score** | | | **Exp Value** | | **Peptide** |
|  |  |  | 414.2202 | 826.4259 | 826.4225 | | | 21 | | | 0.033 | | K.FASFIDK.V |
|  |  |  | 639.3620 | 1276.7095 | 1276.7027 | | | 41 | | | 0.0002 | | K.LALDIEIATYR.K |
|  |  |  | 651.8657 | 1301.7168 | 1301.7078 | | | 80 | | | 2.1e-008 | | R.SLDLDSIIAEVK.A |
| Keratin, type II cytoskeletal 7 | Q29S21 | 156^b^ | **Observed** | **Mr(expt)** | **Mr(calc)** | | | **Score** | | | **Exp Value** | | **Peptide** |
|  |  |  | 547.2735 | 1092.5325 | 1092.5199 | | | 48 | | | 0.00024 | | K.AQYEEIANR.S |
|  |  |  | 639.3674 | 1276.7202 | 1276.7027 | | | 53 | | | 3.4e-005 | | K.LALDIEIATYR.K |
|  |  |  | 651.8691 | 1301.7236 | 1301.7078 | | | 83 | | | 4.6e-008 | | R.SLDLDSIIAEVK.A |
| Keratin, type II cytoskeletal 1 | Q6EIY9 | 174^a^ | **Observed** | **Mr(expt)** | **Mr(calc)** | | | **Score** | | | **Exp Value** | | **Peptide** |
|  |  |  | 692.3523 | 1382.6900 | 1382.6830 | | | 74 | | | 6.1e-008 | | K.SLNNQFASFIDK.V |
|  |  |  | 738.3812 | 1474.7479 | 1474.7416 | | | 71 | | | 1.9e-007 | | K.WELLQQVDTSTR.T |
|  |  |  | 738.3989 | 1474.7832 | 1474.7780 | | | 74 | | | 8.7e-008 | | R.FLEQQNQVLQTK.W |
|  |  |  | 546.9607 | 1637.8604 | 1637.8525 | | | 20 | | | 0.019 | | K.SLNNQFASFIDKVR.F |
| Keratin, type II cytoskeletal 1 | Q6EIY9 | 151^b^ | **Observed** | **Mr(expt)** | **Mr(calc)** | | | **Score** | | | **Exp Value** | | **Peptide** |
|  |  |  | 437.7578 | 873.5010 | 873.4920 | | | 46 | | | 0.00056 | | R.SLVNLGGSK.S |
|  |  |  | 517.2679 | 1032.5212 | 1032.5087 | | | 34 | | | 0.0063 | | R.TLLEGEESR.M |
|  |  |  | 571.2695 | 1140.5245 | 1140.5121 | | | 50 | | | 8e-005 | | R.DYQELMNTK.L |
|  |  |  | 579.2678 | 1156.5211 | 1156.5070 | | | 48 | | | 9.9e-005 | | R.DYQELMNTK.L |
|  |  |  | 633.3288 | 1264.6431 | 1264.6299 | | | 42 | | | 0.00073 | | R.TNAENEFVTIK.K |
|  |  |  | 692.3576 | 1382.7007 | 1382.6830 | | | 65 | | | 2.7e-006 | | K.SLNNQFASFIDK.V |
|  |  |  | 738.3926 | 1474.7705 | 1474.7780 | | | 39 | | | 0.0014 | | R.FLEQQNQVLQTK.W |
| Keratin, type II cytoskeletal 79 | Q148H7 | 136^a^ | **Observed** | **Mr(expt)** | **Mr(calc)** | | | **Score** | | | **Exp Value** | | **Peptide** |
|  |  |  | 414.2202 | 826.4259 | 826.4225 | | | 21 | | | 0.033 | | K.FASFIDK.V |
|  |  |  | 665.3710 | 1328.7274 | 1328.7187 | | | 51 | | | 2.6e-005 | | R.NLDLDSIIAEVK.A |
|  |  |  | 632.3592 | 1262.7036 | 1262.6870 | | | 115 | | | 7.2e-011 | | K.LALDVEIATYR.K |
| Keratin, type II cytoskeletal 79 | Q148H7 | 123^b^ | **Observed** | **Mr(expt)** | **Mr(calc)** | | | **Score** | | | **Exp Value** | | **Peptide** |
|  |  |  | 632.3590 | 1262.7033 | 1262.6870 | | | 77 | | | 1.7e-007 | | K.LALDVEIATYR.K |
|  |  |  | 665.3746 | 1328.7347 | 1328.7187 | | | 75 | | | 3.2e-007 | | R.NLDLDSIIAEVK.A |
| Keratin, type I cytoskeletal 9 | P35527 | 124^a^ | **Observed** | **Mr(expt)** | **Mr(calc)** | | | **Score** | | | **Exp Value** | | **Peptide** |
|  |  |  | 457.2094 | 912.4042 | 912.4011 | | | 21 | | | 0.12 | | R.MTLDDFR.I |
|  |  |  | 530.7870 | 1059.5594 | 1059.5560 | | | 44 | | | 0.0022 | | K.TLLDIDNTR.M |
|  |  |  | 533.2550 | 1064.4954 | 1064.4920 | | | 49 | | | 0.0005 | | K.STMQELNSR.L |
|  |  |  | 603.8072 | 1205.5999 | 1205.5962 | | | 65 | | | 1.3e-005 | | R.QVLDNLTMEK.S |
|  |  |  | 837.3855 | 2509.1348 | 2509.1245 | | | 68 | | | 1e-006 | | K.EIETYHNLLEGGQEDFESSGAGK.I |
| Keratin, type I cytoskeletal 9 | P35527 | 496^b^ | **Observed** | **Mr(expt)** | **Mr(calc)** | | | **Score** | | | **Exp Value** | | **Peptide** |
|  |  |  | 449.2124 | 896.4102 | 896.4062 | | | 36 | | | 0.0079 | | R.MTLDDFR.I |
|  |  |  | 530.7875 | 1059.5605 | 1059.5560 | | | 47 | | | 0.00099 | | K.TLLDIDNTR.M |
|  |  |  | 533.2553 | 1064.4960 | 1064.4920 | | | 48 | | | 0.00061 | | K.STMQELNSR.L |
|  |  |  | 561.2977 | 1120.5809 | 1120.5764 | | | 42 | | | 0.0037 | | R.QEYEQLIAK.N |
|  |  |  | 603.8078 | 1205.6011 | 1205.5962 | | | 39 | | | 0.0054 | | R.QVLDNLTMEK.S |
|  |  |  | 616.8048 | 1231.5950 | 1231.5906 | | | 79 | | | 4.2e-007 | | R.SGGGGGGGLGSGGSIR.S |
|  |  |  | 618.2707 | 1234.5269 | 1234.5215 | | | 78 | | | 1.5e-007 | | R.FSSSSGYGGGSSR.V |
|  |  |  | 793.8895 | 1585.7643 | 1585.7583 | | | 80 | | | 2.4e-007 | | K.VQALEEANNDLENK.I |
|  |  |  | 896.3711 | 1790.7277 | 1790.7205 | | | 153 | | | 1.2e-015 | | R.GGSGGSYGGGGSGGGYGGGSGSR.G |
|  |  |  | 613.3290 | 1836.9651 | 1836.9581 | | | 55 | | | 4.7e-005 | | R.HGVQELEIELQSQLSK.K |
|  |  |  | 777.7110 | 2330.1111 | 2330.1021 | | | 24 | | | 0.051 | | K.SDLEMQYETLQEELMALKK.N |
|  |  |  | 837.3852 | 2509.1339 | 2509.1245 | | | 75 | | | 1.8e-007 | | K.EIETYHNLLEGGQEDFESSGAGK.I |
|  |  |  | 1088.8473 | 3263.5201 | 3263.5066 | | | 96 | | | 1.2e-009 | | K.DIENQYETQITQIEHEVSSSGQEVQSSAK.E |
| Integrin alpha-V | P06756 | 249^a^ | **Observed** | **Mr(expt)** | **Mr(calc)** | | | **Score** | | | **Exp Value** | | **Peptide** |
|  |  |  | 488.2903 | 974.5660 | 974.5623 | | | 38 | | | 0.0042 | | R.MFLLVGAPK.A |
|  |  |  | 500.3034 | 998.5922 | 998.5873 | | | 59 | | | 3.1e-005 | | K.AGTQLLAGLR.F |
|  |  |  | 634.8847 | 1267.7548 | 1267.7500 | | | 40 | | | 0.00075 | | K.VDLAVLAAVEIR.G |
|  |  |  | 666.3847 | 1330.7549 | 1330.7496 | | | 61 | | | 1.3e-005 | | K.LNFQVELLLDK.L |
|  |  |  | 744.8762 | 1487.7378 | 1487.7296 | | | 76 | | | 6.8e-007 | | K.SSASFNVIEFPYK.N |
|  |  |  | 777.8967 | 1553.7788 | 1553.7726 | | | 56 | | | 8.3e-005 | | K.FDLQIQSSNLFDK.V |
|  |  |  | 787.4488 | 1572.8831 | 1572.8763 | | | 76 | | | 2.7e-007 | | K.IYIGDDNPLTLIVK.A |
|  |  |  | 785.0513 | 2352.1320 | 2352.1234 | | | 25 | | | 0.034 | | K.ENPETEEDVGPVVQHIYELR.N |
|  |  |  | 931.1637 | 2790.4694 | 2790.4592 | | | 56 | | | 1.2e-005 | | R.VLLGGPGSFYWQGQLISDQVAEIVSK.Y |
| Integrin alpha-V | P06756 | 241^b^ | **Observed** | **Mr(expt)** | **Mr(calc)** | | | **Score** | | | **Exp Value** | | **Peptide** |
|  |  |  | 500.3030 | 998.5912 | 998.5881 | | | 51 | | | 0.00019 | | K.AGTQLLAGLR.F |
|  |  |  | 634.8842 | 1267.7537 | 1267.7500 | | | 61 | | | 2.7e-005 | | K.VDLAVLAAVEIR.G |
|  |  |  | 666.3847 | 1330.7549 | 1330.7496 | | | 72 | | | 1.8e-006 | | K.LNFQVELLLDK.L |
|  |  |  | 744.8757 | 1487.7369 | 1487.7296 | | | 55 | | | 4.9e-005 | | K.SSASFNVIEFPYK.N |
|  |  |  | 787.4456 | 1572.8767 | 1572.8763 | | | 76 | | | 6.2e-007 | | K.IYIGDDNPLTLIVK.A |
|  |  |  | 931.1631 | 2790.4601 | 2790.4592 | | | 61 | | | 1.9e-005 | | R.VLLGGPGSFYWQGQLISDQVAEIVSK.Y |
| Integrin β2 | P11835 | 146^a^ | **Observed** | **Mr(expt)** | **Mr(calc)** | | | **Score** | | | **Exp Value** | | **Peptide** |
|  |  |  | 485.2575 | 968.5005 | 968.4968 | | | 51 | | | 0.00032 | | R.IGFGSFVDK.T |
|  |  |  | 563.8168 | 1125.6191 | 1125.6142 | | | 29 | | | 0.032 | | K.LGAILTPNDGR.C |
|  |  |  | 681.8275 | 1361.6405 | 1361.6364 | | | 53 | | | 0.00012 | | K.SQWNNDNPLFK.S |
|  |  |  | 930.9851 | 1859.9557 | 1859.9476 | | | 106 | | | 5.4e-010 | | K.SAVGELSDDSSNVVQLIK.N |
| Integrin β2 | P11835 | 127^b^ | **Observed** | **Mr(expt)** | **Mr(calc)** | | | **Score** | | | **Exp Value** | | **Peptide** |
|  |  |  | 485.2569 | 968.4997 | 968.4968 | | | 48 | | | 0.0004 | | R.IGFGSFVDK.T |
|  |  |  | 681.8270 | 1361.6396 | 1361.6364 | | | 35 | | | 0.00071 | | K.SQWNNDNPLFK.S |
|  |  |  | 930.9846 | 1859.9547 | 1859.9476 | | | 104 | | | 9.9e-010 | | K.SAVGELSDDSSNVVQLIK.N |
| Aminopeptidase N | P79143 | 84^a^ | **Observed** | **Mr(expt)** | **Mr(calc)** | | | **Score** | | | **Exp Value** | | **Peptide** |
|  |  |  | 668.8508 | 1335.6870 | 1335.6823 | | | 77 | | | 7.3e-007 | | K.DLVSTLFAEWR.K |
|  |  |  | 720.3759 | 1438.7372 | 1438.7317 | | | 40 | | | 0.0034 | | K.NPQNNPIYPNLR.S |
| Aminopeptidase N | P79143 | 82^b^ | **Observed** | **Mr(expt)** | **Mr(calc)** | | | **Score** | | | **Exp Value** | | **Peptide** |
|  |  |  | 668.8568 | 1335.6990 | 1335.6823 | | | 70 | | | 1.2e-006 | | K.DLVSTLFAEWR.K |
|  |  |  | 720.3814 | 1438.7483 | 1438.7317 | | | 40 | | | 0.0012 | | K.NPQNNPIYPNLR.S |
| Neutral amino acid transporter B(0) | Q95JC7 | 101^a^ | **Observed** | **Mr(expt)** | **Mr(calc)** | | | **Score** | | | **Exp Value** | | **Peptide** |
|  |  |  | 548.8235 | 1095.6324 | 1095.6288 | | | 57 | | | 2.9e-005 | | K.LGPEGELLIR.F |
|  |  |  | 653.3597 | 1304.7048 | 1304.6976 | | | 75 | | | 9.9e-007 | | K.EVLDSFLDLVR.N |
| Neutral amino acid transporter B(0) | Q95JC7 | 84^b^ | **Observed** | **Mr(expt)** | **Mr(calc)** | | | **Score** | | | **Exp Value** | | **Peptide** |
|  |  |  | 548.8239 | 1095.6332 | 1095.6288 | | | 40 | | | 0.00014 | | K.LGPEGELLIR.F |
|  |  |  | 653.3593 | 1304.7040 | 1304.6976 | | | 64 | | | 3.8e-007 | | K.EVLDSFLDLVR.N |
| Thioredoxin-related transmembrane protein 1 | Q0Z7W6 | 100^a^ | **Observed** | **Mr(expt)** | **Mr(calc)** | | | **Score** | | | **Exp Value** | | **Peptide** |
|  |  |  | 629.3320 | 1256.6495 | 1256.6361 | | | 45 | | | 0.0013 | | K.VDVTEQPGLSGR.F |
|  |  |  | 830.0115 | 1659.8110 | 1659.8103 | | | 85 | | | 8.5e-008 | | K.RSDVRIITDENWR.E |
| Thioredoxin-related transmembrane protein 1 | Q0Z7W6 | 93^b^ | **Observed** | **Mr(expt)** | **Mr(calc)** | | | **Score** | | | **Exp Value** | | **Peptide** |
|  |  |  | 629.3320 | 1256.6495 | 1256.6361 | | | 44 | | | 0.00085 | | K.VDVTEQPGLSGR.F |
|  |  |  | 830.0142 | 1659.8115 | 1659.8103 | | | 77 | | | 5.3e-007 | | K.RSDVRIITDENWR.E |
| Leucine-rich repeat-containing protein 59 | Q5E9X4 | 97^a^ | **Observed** | **Mr(expt)** | **Mr(calc)** | | | **Score** | | | **Exp Value** | | **Peptide** |
|  |  |  | 658.4054 | 1314.7963 | 1314.7911 | | | 76 | | | 3.7e-008 | | R.LVTLPVSFAQLK.S |
|  |  |  | 521.3006 | 1560.8801 | 1560.8736 | | | 48 | | | 0.00033 | | R.LVNLQHLDLLNNR.L |
| Leucine-rich repeat-containing protein 59 | Q5E9X4 | 97^b^ | **Observed** | **Mr(expt)** | **Mr(calc)** | | | **Score** | | | **Exp Value** | | **Peptide** |
|  |  |  | 521.3006 | 1560.8801 | 1560.8736 | | | 50 | | | 4.5e-005 | | R.LVNLQHLDLLNNR.L |
|  |  |  | 658.4056 | 1314.7967 | 1314.7911 | | | 73 | | | 2.3e-007 | | R.LVTLPVSFAQLK.S |
| Clathrin heavy chain 1 | Q68FD5 | 245^a^ | **Observed** | **Mr(expt)** | **Mr(calc)** | | | **Score** | | | **Exp Value** | | **Peptide** |
|  |  |  | 555.8406 | 1109.6667 | 1109.6597 | | | 30 | | | 0.00096 | | K.LLLPWLEAR.I |
|  |  |  | 677.4302 | 1352.8459 | 1352.8391 | | | 68 | | | 1.7e-007 | | R.NLQNLLILTAIK.A |
|  |  |  | 740.4073 | 1478.8001 | 1478.7922 | | | 69 | | | 1.7e-007 | | K.VGYTPDWIFLLR.N |
|  |  |  | 971.9647 | 1941.9148 | 1941.9068 | | | 99 | | | 1.6e-010 | | R.TSIDAYDNFDNISLAQR.L |
|  |  |  | 974.0150 | 1946.0154 | 1946.0070 | | | 49 | | | 1.2e-005 | | K.AFMTADLPNELIELLEK.I |
|  |  |  | 691.0216 | 2070.0429 | 2070.0343 | | | 30 | | | 0.0012 | | R.GYFEELITMLEAALGLER.A |
| Clathrin heavy chain 1 | Q68FD5 | 133^b^ | **Observed** | **Mr(expt)** | **Mr(calc)** | | | **Score** | | | **Exp Value** | | **Peptide** |
|  |  |  | 555.8399 | 1109.6652 | 1109.6597 | | | 17 | | | 0.021 | | K.LLLPWLEAR.I |
|  |  |  | 677.4301 | 1352.8457 | 1352.8391 | | | 63 | | | 4.8e-007 | | R.NLQNLLILTAIK.A |
|  |  |  | 974.0155 | 1946.0164 | 1946.0070 | | | 49 | | | 1.2e-005 | | K.AFMTADLPNELIELLEK.I |
|  |  |  | 685.6894 | 2054.0463 | 2054.0394 | | | 62 | | | 1e-006 | | R.GYFEELITMLEAALGLER.A |
| V-type proton ATPase 116 kDa subunit a isoform 1 | Q29466 | 104^a^ | **Observed** | **Mr(expt)** | **Mr(calc)** | | | **Score** | | | **Exp Value** | | **Peptide** |
|  |  |  | 553.8103 | 1105.6061 | 1105.6019 | | | 39 | | | 0.0052 | | R.NFLELTELK.F |
|  |  |  | 711.8400 | 1421.6654 | 1421.6609 | | | 68 | | | 4e-006 | | R.MQTNQTPPTYNK.T |
|  |  |  | 771.4259 | 1540.8373 | 1540.8290 | | | 66 | | | 5.5e-006 | | K.SVFIIFFQGDQLK.N |
| V-type proton ATPase 116 kDa subunit a isoform 1 | Q29466 | 254^b^ | **Observed** | **Mr(expt)** | **Mr(calc)** | | | **Score** | | | **Exp Value** | | **Peptide** |
|  |  |  | 523.3131 | 1044.6117 | 1044.6080 | | | 72 | | | 1.6e-006 | | R.LGFVAGVINR.E |
|  |  |  | 553.8108 | 1105.6071 | 1105.6019 | | | 41 | | | 0.0035 | | R.NFLELTELK.F |
|  |  |  | 711.8398 | 1421.6650 | 1421.6609 | | | 55 | | | 7.5e-005 | | R.MQTNQTPPTYNK.T |
|  |  |  | 771.4257 | 1540.8369 | 1540.8290 | | | 73 | | | 8.6e-007 | | K.SVFIIFFQGDQLK.N |
|  |  |  | 614.9619 | 1841.8640 | 1841.8578 | | | 43 | | | 0.00089 | | R.IDDLQMVLNQTEDHR.Q |
|  |  |  | 1043.0115 | 2084.0084 | 2084.0004 | | | 97 | | | 3.2e-009 | | K.FTYGFQNIVDAYGIGTYR.E |
|  |  |  | 718.6853 | 2153.0340 | 2153.0277 | | | 43 | | | 0.00071 | | R.QAEIENPLEDPVTGDYVHK.S |
| CD44 antigen | Q28284 | 99^a^ | **Observed** | **Mr(expt)** | **Mr(calc)** | | | **Score** | | | **Exp Value** | | **Peptide** |
|  |  |  | 462.9244 | 1385.7514 | 1385.7456 | | | 62 | | | 2.3e-005 | | R.YGFIEGHVVIPR.I |
|  |  |  | 756.1013 | 3020.3762 | 3020.3669 | | | 42 | | | 6.3e-005 | | K.SQEMVHLVNKEPSETPDQYTTADETR.N |
| CD44 antigen | Q28284 | 83^b^ | **Observed** | **Mr(expt)** | **Mr(calc)** | | | **Score** | | | **Exp Value** | | **Peptide** |
|  |  |  | 462.9244 | 1385.7514 | 1385.7456 | | | 38 | | | 0.0047 | | R.YGFIEGHVVIPR.I |
|  |  |  | 756.1011 | 3020.3759 | 3020.3669 | | | 72 | | | 5.7e-007 | | K.SQEMVHLVNKEPSETPDQYTTADETR.N |
| Calnexin | P24643 | 204^a^ | **Observed** | **Mr(expt)** | **Mr(calc)** | | | **Score** | | | **Exp Value** | | **Peptide** |
|  |  |  | 531.3059 | 1060.5972 | 1060.5917 | | | 49 | | | 2.6e-005 | | R.GTLSGWILSK.A |
|  |  |  | 635.3154 | 1268.6163 | 1268.6111 | | | 42 | | | 0.00018 | | K.TPYTIMFGPDK.C |
|  |  |  | 486.2428 | 1455.7067 | 1455.6994 | | | 22 | | | 0.021 | | K.TPELNLDQFHDK.T |
|  |  |  | 737.8557 | 1473.6968 | 1473.6889 | | | 51 | | | 1.4e-005 | | R.VVDDWANDGWGLK.K |
|  |  |  | 854.4197 | 1706.8248 | 1706.8192 | | | 49 | | | 1.7e-005 | | K.IPNPDFFEDLEPFK.M |
|  |  |  | 885.9243 | 1769.8341 | 1769.8261 | | | 101 | | | 7.3e-011 | | K.APVPTGEVYFADSFDR.G |
| Calnexin | P24643 | 184^b^ | **Observed** | **Mr(expt)** | **Mr(calc)** | | | **Score** | | | **Exp Value** | | **Peptide** |
|  |  |  | 531.3098 | 1060.6051 | 1060.5917 | | | 66 | | | 2.8e-006 | | R.GTLSGWILSK.A |
|  |  |  | 737.8614 | 1473.7082 | 1473.6889 | | | 54 | | | 3.7e-005 | | R.VVDDWANDGWGLK.K |
|  |  |  | 854.4281 | 1706.8417 | 1706.8192 | | | 69 | | | 1e-006 | | K.IPNPDFFEDLEPFK.M |
|  |  |  | 885.9308 | 1769.8470 | 1769.8261 | | | 82 | | | 4.2e-008 | | K.APVPTGEVYFADSFDR.G |
| Thromboxane-A synthase | Q2KIG5 | 127^a^ | **Observed** | **Mr(expt)** | **Mr(calc)** | | | **Score** | | | **Exp Value** | | **Peptide** |
|  |  |  | 393.2627 | 785.5181 | 785.5155 | | | 63 | | | 5.5e-007 | | R.LGLLELK.A |
|  |  |  | 656.8552 | 1311.6959 | 1311.6782 | | | 89 | | | 2e-009 | | K.PLLVLLLSFPSI.M |
| Thromboxane-A synthase | Q2KIG5 | 91^b^ | **Observed** | **Mr(expt)** | **Mr(calc)** | | | **Score** | | | **Exp Value** | | **Peptide** |
|  |  |  | 393.2624 | 785.5176 | 785.5155 | | | 66 | | | 5.5e-007 | | R.LGLLELK.A |
|  |  |  | 656.8550 | 1311.6954 | 1311.6782 | | | 52 | | | 2.3e-005 | | K.PLLVLLLSFPSI.M |
| Transmembrane emp24 domain-containing protein 9 | Q3T133 | 114^a^ | **Observed** | **Mr(expt)** | **Mr(calc)** | | | **Score** | | | **Exp Value** | | **Peptide** |
|  |  |  | 671.3743 | 1340.7340 | 1340.7300 | | | 92 | | | 3.9e-009 | | R.QLVEQVEQIQK.E |
|  |  |  | 364.6858 | 727.3571 | 727.3541 | | | 48 | | | 9.4e-005 | | K.SFFEAK.K |
| Transmembrane emp24 domain-containing protein 9 | Q3T133 | 153^b^ | **Observed** | **Mr(expt)** | **Mr(calc)** | | | **Score** | | | **Exp Value** | | **Peptide** |
|  |  |  | 671.3745 | 1340.7344 | 1340.7300 | | | 89 | | | 1.3e-008 | | R.QLVEQVEQIQK.E |
|  |  |  | 364.6857 | 727.3569 | 727.3541 | | | 92 | | | 2.8e-009 | | K.SFFEAK.K |
| Peptidyl-prolyl cis-trans isomerase B | P80311 | 69^a^ | **Observed** | **Mr(expt)** | **Mr(calc)** | | | **Score** | | | **Exp Value** | | **Peptide** |
|  |  |  | 416.7698 | 831.5251 | 831.5218 | | | 34 | | | 0.00042 | | R.VVIGLFGK.T |
|  |  |  | 682.8585 | 1363.7025 | 1363.6983 | | | 55 | | | 3.6e-006 | | K.TVDNFVALATGEK.G |
| Peptidyl-prolyl cis-trans isomerase B | P80311 | 89^b^ | **Observed** | **Mr(expt)** | **Mr(calc)** | | | **Score** | | | **Exp Value** | | **Peptide** |
|  |  |  | 416.7700 | 831.5255 | 831.5218 | | | 32 | | | 0.0035 | | R.VVIGLFGK.T |
|  |  |  | 682.8589 | 1363.7032 | 1363.6983 | | | 74 | | | 1.4e-006 | | K.TVDNFVALATGEK.G |
| Endoplasmin (fragment) | O18750 | 84^a^ | **Observed** | **Mr(expt)** | **Mr(calc)** | | | **Score** | | | **Exp Value** | | **Peptide** |
|  |  |  | 638.3275 | 1274.6404 | 1274.6354 | | | 53 | | | 1.1e-005 | | R.ELISNASDALDK.I |
|  |  |  | 743.3844 | 1484.7542 | 1484.7471 | | | 54 | | | 9.4e-006 | | K.GVVDSDDLPLNVSR.E |
| Endoplasmin (fragment) | O18750 | 178^b^ | **Observed** | **Mr(expt)** | **Mr(calc)** | | | **Score** | | | **Exp Value** | | **Peptide** |
|  |  |  | 594.3454 | 1186.6763 | 1186.6710 | | | 36 | | | 0.00062 | | K.SILFVPTSAPR.G |
|  |  |  | 638.3277 | 1274.6408 | 1274.6354 | | | 74 | | | 9.6e-008 | | R.ELISNASDALDK.I |
|  |  |  | 653.8322 | 1305.6498 | 1305.6427 | | | 27 | | | 0.0048 | | K.EFEPLLNWMK.D |
|  |  |  | 743.3841 | 1484.7536 | 1484.7471 | | | 57 | | | 4.6e-006 | | K.GVVDSDDLPLNVSR.E |
|  |  |  | 763.3706 | 1524.7267 | 1524.7195 | | | 78 | | | 4.4e-008 | | K.EEASDYLELDTIK.N |
| Endoplasmic reticulum resident protein 29 | P81623 | 79^a^ | **Observed** | **Mr(expt)** | **Mr(calc)** | | | **Score** | | | **Exp Value** | | **Peptide** |
|  |  |  | 567.8330 | 1133.6514 | 1133.6444 | | | 49 | | | 7.4e-005 | | K.SLNILTAFQK.K |
|  |  |  | 780.5667 | 1560.6709 | 1560.6700 | | | 58 | | | 1.7e-005 | | K.RLAENSASSDDLLVA.E |
| Endoplasmic reticulum resident protein 29 | P81623 | 86^b^ | **Observed** | **Mr(expt)** | **Mr(calc)** | | | **Score** | | | **Exp Value** | | **Peptide** |
|  |  |  | 567.8332 | 1133.6592 | 1133.6444 | | | 60 | | | 3.09e-06 | | K.SLNILTAFQK.K |
|  |  |  | 780.3395 | 1560.6717 | 1560.6700 | | | 54 | | | 2.1e-005 | | K.RLAENSASSDDLLVA.E |
| Protein disulfide-isomerase A3 | P38657 | 88^a^ | **Observed** | **Mr(expt)** | **Mr(calc)** | | | **Score** | | | **Exp Value** | | **Peptide** |
|  |  |  | 542.7899 | 1083.5653 | 1083.5601 | | | 14 | | | 0.12 | | K.YGVSGYPTLK.I |
|  |  |  | 586.7767 | 1171.5389 | 1171.5332 | | | 35 | | | 0.00072 | | K.FVMQEEFSR.D |
|  |  |  | 832.8867 | 1663.7588 | 1663.7512 | | | 75 | | | 3.2e-008 | | K.MDATANDVPSPYEVR.G |
| Protein disulfide-isomerase A3 | P38657 | 194^b^ | **Observed** | **Mr(expt)** | **Mr(calc)** | | | **Score** | | | **Exp Value** | | **Peptide** |
|  |  |  | 542.7947 | 1083.5748 | 1083.5601 | | | 53 | | | 6.6e-005 | | K.YGVSGYPTLK.I |
|  |  |  | 586.7807 | 1171.5468 | 1171.5332 | | | 37 | | | 0.0019 | | K.FVMQEEFSR.D |
|  |  |  | 596.3108 | 1190.6070 | 1190.5931 | | | 43 | | | 0.0007 | | K.LAPEYEAAATR.L |
|  |  |  | 671.3536 | 1340.6927 | 1340.6765 | | | 53 | | | 5.8e-005 | | R.GFPTIYFSPANK.K |
|  |  |  | 677.8544 | 1353.6942 | 1353.6776 | | | 39 | | | 0.0015 | | R.EATNPPVIQEEK.P |
|  |  |  | 684.8437 | 1367.6727 | 1367.6569 | | | 29 | | | 0.012 | | K.SEPIPESNDGPVK.V |
|  |  |  | 832.8924 | 1663.7702 | 1663.7512 | | | 77 | | | 1.4e-007 | | K.MDATANDVPSPYEVR.G |
| Protein disulfide-isomerase | P05307 | 119^a^ | **Observed** | **Mr(expt)** | **Mr(calc)** | | | **Score** | | | **Exp Value** | | **Peptide** |
|  |  |  | 374.7354 | 747.4562 | 747.4531 | | | 32 | | | 0.00085 | | K.LLDFIK.H |
|  |  |  | 382.2225 | 762.4304 | 762.4276 | | | 22 | | | 0.035 | | K.IFGGEIK.T |
|  |  |  | 483.7888 | 965.5630 | 965.5586 | | | 43 | | | 8e-005 | | R.ILEFFGLK.K |
|  |  |  | 541.3445 | 1080.6745 | 1080.6695 | | | 35 | | | 0.00032 | | K.THILLFLPK.S |
|  |  |  | 890.9249 | 1779.8352 | 1779.8275 | | | 71 | | | 8.7e-008 | | K.VDATEESDLAQQYGVR.G |
| Protein disulfide-isomerase | P05307 | 156^b^ | **Observed** | **Mr(expt)** | **Mr(calc)** | | | **Score** | | | **Exp Value** | | **Peptide** |
|  |  |  | 374.7383 | 747.4620 | 747.4531 | | | 33 | | | 0.0052 | | K.LLDFIK.H |
|  |  |  | 483.7930 | 965.5715 | 965.5586 | | | 36 | | | 0.0019 | | R.ILEFFGLK.K |
|  |  |  | 519.2539 | 1036.4932 | 1036.4825 | | | 27 | | | 0.02 | | R.NNFEGEVTK.E |
|  |  |  | 541.3484 | 1080.6822 | 1080.6695 | | | 39 | | | 0.00022 | | K.THILLFLPK.S |
|  |  |  | 647.3105 | 1292.6064 | 1292.5918 | | | 62 | | | 5.7e-006 | | K.MDSTANEVEAVK.V |
|  |  |  | 890.9309 | 1779.8473 | 1779.8275 | | | 62 | | | 4.3e-006 | | K.VDATEESDLAQQYGVR.G |
| Dolichyl-diphosphooligosaccharide-protein glycosyltransferase 48 kDa subunit | A6QPY0 | 135^a^ | **Observed** | **Mr(expt)** | **Mr(calc)** | | | **Score** | | | **Exp Value** | | **Peptide** |
|  |  |  | 523.8054 | 1045.5962 | 1045.5920 | | | 35 | | | 0.012 | | K.SSLNPILFR.G |
|  |  |  | 585.3558 | 1168.6971 | 1168.6928 | | | 69 | | | 1.5e-006 | | K.NTLLIAGLQAR.N |
|  |  |  | 642.3907 | 1282.7669 | 1282.7609 | | | 51 | | | 8.2e-005 | | R.TLVLLDNLNLR.E |
|  |  |  | 656.8537 | 1311.6927 | 1311.6863 | | | 52 | | | 0.00018 | | K.LPDVYGVFQFK.V |
|  |  |  | 918.4555 | 1834.8964 | 1834.8890 | | | 49 | | | 0.00027 | | K.WVPFDGDDIQLEFVR.I |
| Dolichyl-diphosphooligosaccharide-protein glycosyltransferase 48 kDa subunit | A6QPY0 | 101^b^ | **Observed** | **Mr(expt)** | **Mr(calc)** | | | **Score** | | | **Exp Value** | | **Peptide** |
|  |  |  | 523.8094 | 1045.6043 | 1045.5920 | | | 29 | | | 0.015 | | K.SSLNPILFR.G |
|  |  |  | 580.3212 | 1158.6279 | 1158.6132 | | | 38 | | | 0.0022 | | K.TADDPSLSLIK.Y |
|  |  |  | 585.3611 | 1168.7076 | 1168.6928 | | | 61 | | | 2.1e-006 | | K.NTLLIAGLQAR.N |
|  |  |  | 642.3949 | 1282.7753 | 1282.7609 | | | 28 | | | 0.0048 | | R.TLVLLDNLNLR.E |
|  |  |  | 656.8585 | 1311.7025 | 1311.6863 | | | 40 | | | 0.0011 | | K.LPDVYGVFQFK.V |
|  |  |  | 918.4638 | 1834.9131 | 1834.8890 | | | 36 | | | 0.0018 | | K.WVPFDGDDIQLEFVR.I |
| Endoplasmin | P41148 | 241^a^ | **Observed** | **Mr(expt)** | **Mr(calc)** | | | **Score** | | | **Exp Value** | | **Peptide** |
|  |  |  | 541.2777 | 1080.5408 | 1080.5352 | | | 44 | | | 0.00019 | | K.FAFQAEVNR.M |
|  |  |  | 594.3454 | 1186.6763 | 1186.6710 | | | 36 | | | 0.00062 | | K.SILFVPTSAPR.G |
|  |  |  | 638.3277 | 1274.6408 | 1274.6354 | | | 74 | | | 9.6e-008 | | R.ELISNASDALDK.I |
|  |  |  | 645.3066 | 1288.5987 | 1288.5935 | | | 45 | | | 5.4e-005 | | K.DISTNYYASQK.K |
|  |  |  | 653.8322 | 1305.6498 | 1305.6427 | | | 27 | | | 0.0048 | | K.EFEPLLNWMK.D |
|  |  |  | 743.3841 | 1484.7536 | 1484.7471 | | | 57 | | | 4.6e-006 | | K.GVVDSDDLPLNVSR.E |
|  |  |  | 763.3706 | 1524.7267 | 1524.7195 | | | 78 | | | 4.4e-008 | | K.EEASDYLELDTIK.N |
|  |  |  | 515.6168 | 1543.8285 | 1543.8205 | | | 18 | | | 0.034 | | R.ELISNASDALDKIR.L |
| Endoplasmin | P41148 | 200^b^ | **Observed** | **Mr(expt)** | **Mr(calc)** | | | **Score** | | | **Exp Value** | | **Peptide** |
|  |  |  | 482.3031 | 962.5916 | 962.5800 | | | 35 | | | 0.0014 | | K.LIINSLYK.N |
|  |  |  | 541.2809 | 1080.5473 | 1080.5352 | | | 36 | | | 0.0035 | | K.FAFQAEVNR.M |
|  |  |  | 575.7789 | 1149.5432 | 1149.5302 | | | 43 | | | 0.00069 | | K.EAESSPFVER.L |
|  |  |  | 594.3511 | 1186.6877 | 1186.6710 | | | 34 | | | 0.0036 | | K.SILFVPTSAPR.G |
|  |  |  | 638.3325 | 1274.6504 | 1274.6354 | | | 68 | | | 2.1e-006 | | R.ELISNASDALDK.I |
|  |  |  | 645.3116 | 1288.6087 | 1288.5935 | | | 35 | | | 0.0031 | | K.DISTNYYASQK.K |
|  |  |  | 743.3898 | 1484.7650 | 1484.7471 | | | 64 | | | 3.9e-006 | | K.GVVDSDDLPLNVSR.E |
|  |  |  | 763.3766 | 1524.7386 | 1524.7195 | | | 79 | | | 1e-007 | | K.EEASDYLELDTIK.N |
|  |  |  | 749.3622 | 2245.0648 | 2245.0400 | | | 30 | | | 0.0053 | | R.FQSSHHPSDITSLDQYVER.M |
| Rab5C | P51147 | 127^a^ | **Observed** | **Mr(expt)** | **Mr(calc)** | | | **Score** | | | **Exp Value** | | **Peptide** |
|  |  |  | 543.3374 | 1084.6602 | 1084.6492 | | | 34 | | | 0.0029 | | K.LVLLGESAVGK.S |
|  |  |  | 636.8200 | 1271.6255 | 1271.6106 | | | 59 | | | 1.3e-005 | | R.GVDLQENSPASR.S |
|  |  |  | 676.3251 | 1350.6356 | 1350.6204 | | | 52 | | | 6.7e-005 | | K.FEIWDTAGQER.Y |
|  |  |  | 1013.5284 | 2025.0422 | 2025.0167 | | | 64 | | | 1.7e-006 | | R.GAQAAIVVYDITNTDTFAR.A |
| Rab5C | P51147 | 410^b^ | **Observed** | **Mr(expt)** | **Mr(calc)** | | | **Score** | | | **Exp Value** | | **Peptide** |
|  |  |  | 543.3339 | 1084.6532 | 1084.6492 | | | 51 | | | 0.00019 | | K.LVLLGESAVGK.S |
|  |  |  | 636.8148 | 1271.6151 | 1271.6106 | | | 63 | | | 1.3e-005 | | R.GVDLQENSPASR.S |
|  |  |  | 676.3202 | 1350.6258 | 1350.6204 | | | 72 | | | 1.8e-006 | | K.FEIWDTAGQER.Y |
|  |  |  | 698.4039 | 1394.7933 | 1394.7881 | | | 52 | | | 7.8e-005 | | R.QASPNIVIALAGNK.A |
|  |  |  | 784.8971 | 1567.7796 | 1567.7738 | | | 73 | | | 1.3e-006 | | K.TAMNVNEIFMAIAK.K |
|  |  |  | 1013.5196 | 2025.0246 | 2025.0167 | | | 104 | | | 6.1e-010 | | R.GAQAAIVVYDITNTDTFAR.A |
|  |  |  | 898.7502 | 2693.2287 | 2693.2166 | | | 86 | | | 1.5e-008 | | R.AVEFQEAQAYADDNSLLFMETSAK.T |
| Transferrin receptor protein 1 | Q9GLD3 | 143^a^ | **Observed** | **Mr(expt)** | **Mr(calc)** | | | **Score** | | | **Exp Value** | | **Peptide** |
|  |  |  | 408.7417 | 815.4688 | 815.4654 | | | 35 | | | 0.031 | | R.FPIVNAR.I |
|  |  |  | 518.8151 | 1035.6157 | 1035.6117 | | | 47 | | | 0.00033 | | R.IFNVFGVIK.G |
|  |  |  | 665.3930 | 1328.7714 | 1328.7663 | | | 84 | | | 4.2e-008 | | K.SSVGTALLLELAR.I |
|  |  |  | 710.4117 | 1418.8089 | 1418.8020 | | | 73 | | | 5.8e-007 | | K.VSASPLLYSLLEK.T |
| Transferrin receptor protein 1 | Q9GLD3 | 134^b^ | **Observed** | **Mr(expt)** | **Mr(calc)** | | | **Score** | | | **Exp Value** | | **Peptide** |
|  |  |  | 518.8152 | 1035.6157 | 1035.6117 | | | 51 | | | 1.1e-005 | | R.IFNVFGVIK.G |
|  |  |  | 665.3926 | 1328.7705 | 1328.7663 | | | 45 | | | 3e-005 | | K.SSVGTALLLELAR.I |
|  |  |  | 710.4116 | 1418.8087 | 1418.8020 | | | 79 | | | 1.6e-008 | | K.VSASPLLYSLIEK.T |
| Rab7A | P18067 | 201^a^ | **Observed** | **Mr(expt)** | **Mr(calc)** | | | **Score** | | | **Exp Value** | | **Peptide** |
|  |  |  | 518.7935 | 1035.5725 | 1035.5601 | | | 42 | | | 0.00074 | | K.ATIGADFLTK.E |
|  |  |  | 529.3223 | 1056.6300 | 1056.6179 | | | 30 | | | 0.0093 | | K.VIILGDSGVGK.T |
|  |  |  | 594.3223 | 1186.6301 | 1186.6135 | | | 50 | | | 0.00014 | | R.FQSLGVAFYR.G |
|  |  |  | 738.3891 | 1474.7637 | 1474.7456 | | | 53 | | | 5e-005 | | R.DPENFPFVVLGNK.I |
|  |  |  | 795.4281 | 1588.8416 | 1588.8209 | | | 87 | | | 1.4e-008 | | K.EAINVEQAFQTIAR.N |
|  |  |  | 824.4211 | 1646.8276 | 1646.8086 | | | 80 | | | 9.2e-008 | | R.LVTMQIWDTAGQER.F |
| Rab7A | P18067 | 408^b^ | **Observed** | **Mr(expt)** | **Mr(calc)** | | | **Score** | | | **Exp Value** | | **Peptide** |
|  |  |  | 518.7892 | 1035.5638 | 1035.5601 | | | 49 | | | 0.00051 | | K.ATIGADFLTK.E |
|  |  |  | 529.3179 | 1056.6211 | 1056.6179 | | | 46 | | | 0.00064 | | K.VIILGDSGVGK.T |
|  |  |  | 594.3166 | 1186.6187 | 1186.6135 | | | 47 | | | 0.00087 | | R.FQSLGVAFYR.G |
|  |  |  | 599.3021 | 1196.5897 | 1196.5859 | | | 58 | | | 5.2e-005 | | K.TSLMNQYVNK.K |
|  |  |  | 738.3834 | 1474.7523 | 1474.7456 | | | 63 | | | 1.7e-005 | | R.DPENFPFVVLGNK.I |
|  |  |  | 795.4206 | 1588.8267 | 1588.8209 | | | 80 | | | 2.9e-007 | | K.EAINVEQAFQTIAR.N |
|  |  |  | 824.4147 | 1646.8149 | 1646.8086 | | | 95 | | | 1e-008 | | R.LVTMQIWDTAGQER.F |
|  |  |  | 645.3337 | 1932.9792 | 1932.9694 | | | 38 | | | 0.0039 | | K.TLDSWRDEFLIQASPR.D |
|  |  |  | 982.9825 | 1963.9504 | 1963.9415 | | | 78 | | | 2.5e-007 | | K.QETEVELYNEFPEPIK.L |
|  |  |  | 797.7440 | 2390.2102 | 2390.2005 | | | 32 | | | 0.0074 | | R.NALKQETEVELYNEFPEPIK.L |
| V-type ATPase catalytic subunit A | P31404 | 437^a^ | **Observed** | **Mr(expt)** | **Mr(calc)** | | | **Score** | | | **Exp Value** | | **Peptide** |
|  |  |  | 451.7569 | 901.4993 | 901.4981 | | | 51 | | | 0.00055 | | R.GVNVSALSR.D |
|  |  |  | 481.7813 | 961.5480 | 961.5444 | | | 24 | | | 0.13 | | K.TVISQSLSK.Y |
|  |  |  | 517.7523 | 1033.4901 | 1033.4862 | | | 38 | | | 0.0056 | | R.GNEMSEVLR.D |
|  |  |  | 624.8343 | 1247.6541 | 1247.6485 | | | 43 | | | 0.002 | | K.FSMVQVWPVR.Q |
|  |  |  | 758.4034 | 1514.7922 | 1514.7875 | | | 88 | | | 5.7e-008 | | R.TALVANTSNMPVAAR.E |
|  |  |  | 875.4423 | 1748.8701 | 1748.8621 | | | 42 | | | 0.0015 | | R.EASIYTGITLSEYFR.D |
|  |  |  | 891.4335 | 1780.8524 | 1780.8454 | | | 75 | | | 6.1e-007 | | R.LAEMPADSGYPAYLGAR.L |
|  |  |  | 892.9215 | 1783.8284 | 1783.8199 | | | 82 | | | 1.1e-007 | | K.ADYAQLLEDMQNAFR.S |
|  |  |  | 899.4302 | 1796.8459 | 1796.8403 | | | 52 | | | 0.00011 | | R.LAEMPADSGYPAYLGAR.L |
|  |  |  | 676.0100 | 2025.0083 | 2024.9989 | | | 49 | | | 0.00022 | | K.IKADYAQLLEDMQNAFR.S |
|  |  |  | 1028.0504 | 2054.0862 | 2054.0783 | | | 99 | | | 1.4e-009 | | K.EILQEEEDLAEIVQLVGK.A |
|  |  |  | 729.3901 | 2185.1484 | 2185.1379 | | | 61 | | | 9.4e-006 | | R.VGSHITGGDIYGIVNENSLIK.H |
| V-type ATPase catalytic subunit A | P31404 | 374^b^ | **Observed** | **Mr(expt)** | **Mr(calc)** | | | **Score** | | | **Exp Value** | | **Peptide** |
|  |  |  | 517.7523 | 1033.4900 | 1033.4862 | | | 47 | | | 5.8e-005 | | R.GNEMSEVLR.D |
|  |  |  | 624.8342 | 1247.6539 | 1247.6485 | | | 54 | | | 1.4e-005 | | K.FSMVQVWPVR.Q |
|  |  |  | 758.4038 | 1514.7931 | 1514.7875 | | | 86 | | | 5.5e-009 | | R.TALVANTSNMPVAAR.E |
|  |  |  | 891.4342 | 1780.8538 | 1780.8454 | | | 72 | | | 6.3e-008 | | R.LAEMPADSGYPAYLGAR.L |
|  |  |  | 892.9218 | 1783.8291 | 1783.8199 | | | 69 | | | 1.2e-007 | | K.ADYAQLLEDMQNAFR.S |
|  |  |  | 899.4307 | 1796.8469 | 1796.8403 | | | 59 | | | 1.3e-006 | | R.LAEMPADSGYPAYLGAR.L |
|  |  |  | 910.4352 | 1818.8557 | 1818.8467 | | | 87 | | | 2.4e-009 | | K.TVGMLSNMIAFYDMAR.R |
|  |  |  | 685.7029 | 2054.0868 | 2054.0783 | | | 43 | | | 4.9e-005 | | K.EILQEEEDLAEIVQLVGK.A |
| V-type ATPase subunit d1 | P61420 | 146^a^ | **Observed** | **Mr(expt)** | **Mr(calc)** | | | **Score** | | | **Exp Value** | | **Peptide** |
|  |  |  | 615.8482 | 1229.6818 | 1229.6768 | | | 30 | | | 0.033 | | R.LYPEGLAQLAR.A |
|  |  |  | 631.2930 | 1260.5714 | 1260.5662 | | | 52 | | | 0.00013 | | K.NVADYYPEYK.L |
|  |  |  | 652.8299 | 1303.6453 | 1303.6408 | | | 64 | | | 1.8e-005 | | K.LLFEGAGSNPGDK.T |
|  |  |  | 992.5000 | 2974.4782 | 2974.4672 | | | 89 | | | 1e-008 | | K.LHLQSTDYGNFLANEASPLTVSVIDDR.L |
| V-type ATPase subunit d1 | P61420 | 156^b^ | **Observed** | **Mr(expt)** | **Mr(calc)** | | | **Score** | | | **Exp Value** | | **Peptide** |
|  |  |  | 398.7094 | 795.4043 | 795.3949 | | | 38 | | | 0.0027 | | K.MVVEFR.H |
|  |  |  | 510.7612 | 1019.5078 | 1019.4964 | | | 36 | | | 0.0037 | | K.AYLESFYK.F |
|  |  |  | 615.8522 | 1229.6898 | 1229.6768 | | | 29 | | | 0.013 | | R.LYPEGLAQLAR.A |
|  |  |  | 631.2978 | 1260.5810 | 1260.5662 | | | 55 | | | 2.8e-005 | | K.NVADYYPEYK.L |
|  |  |  | 652.8347 | 1303.6549 | 1303.6408 | | | 78 | | | 2.4e-007 | | K.LLFEGAGSNPGDK.T |
|  |  |  | 992.5095 | 2974.5067 | 2974.4672 | | | 61 | | | 2.1e-006 | | K.LHLQSTDYGNFLANEASPLTVSVIDDR.L |
| V-type ATPase subunit C1 | P21282 | 162^a^ | **Observed** | **Mr(expt)** | **Mr(calc)** | | | **Score** | | | **Exp Value** | | **Peptide** |
|  |  |  | 423.7417 | 845.4689 | 845.4647 | | | 53 | | | 0.00045 | | K.FNIPDLK.V |
|  |  |  | 472.2543 | 942.4940 | 942.4883 | | | 37 | | | 0.0084 | | K.GNLQNLER.K |
|  |  |  | 551.7919 | 1101.5692 | 1101.5666 | | | 34 | | | 0.022 | | K.GVTQIDNDLK.S |
|  |  |  | 814.9624 | 1627.9103 | 1627.9033 | | | 80 | | | 1.2e-007 | | K.VGTLDVLVGLSDELAK.L |
|  |  |  | 764.7656 | 2291.2750 | 2291.2665 | | | 32 | | | 0.0026 | | K.KDDFVLDSEYLVTLLVVVPK.L |
|  |  |  | 896.1718 | 2685.4936 | 2685.4841 | | | 76 | | | 6.2e-008 | | K.VGTLDVLVGLSDELAKLDAFVEGVVK.K |
| V-type ATPase subunit C1 | P21282 | 104^b^ | **Observed** | **Mr(expt)** | **Mr(calc)** | | | **Score** | | | **Exp Value** | | **Peptide** |
|  |  |  | 538.8090 | 1075.6034 | 1075.5914 | | | 46 | | | 0.00028 | | K.LDAFVEGVVK.K |
|  |  |  | 551.7961 | 1101.5775 | 1101.5666 | | | 30 | | | 0.019 | | K.GVTQIDNDLK.S |
|  |  |  | 814.9701 | 1627.9256 | 1627.9033 | | | 80 | | | 2.6e-008 | | K.VGTLDVLVGLSDELAK.L |
| V-type ATPase subunit B | P31408 | 289^a^ | **Observed** | **Mr(expt)** | **Mr(calc)** | | | **Score** | | | **Exp Value** | | **Peptide** |
|  |  |  | 552.7729 | 1103.5313 | 1103.5281 | | | 63 | | | 1.9e-005 | | R.TPVSEDMLGR.V |
|  |  |  | 654.8235 | 1307.6325 | 1307.6258 | | | 35 | | | 0.012 | | R.NFIAQGPYENR.T |
|  |  |  | 719.3755 | 1436.7365 | 1436.7300 | | | 56 | | | 6.5e-005 | | R.IPQSTLSEFYPR.D |
|  |  |  | 760.9046 | 1519.7947 | 1519.7882 | | | 81 | | | 2.1e-007 | | K.AVVQVFEGTSGIDAK.K |
|  |  |  | 798.9623 | 1595.9101 | 1595.9035 | | | 41 | | | 0.00053 | | R.QIYPPINVLPSLSR.L |
|  |  |  | 853.9633 | 1705.9121 | 1705.9039 | | | 66 | | | 3.7e-006 | | R.TVYETLDIGWQLLR.I |
|  |  |  | 571.6515 | 1711.9326 | 1711.9257 | | | 26 | | | 0.039 | | R.YAEIVHLTLPDGTKR.S |
|  |  |  | 956.9469 | 1911.8792 | 1911.8713 | | | 37 | | | 0.0028 | | R.GFPGYMYTDLATIYER.A |
|  |  |  | 640.0109 | 1917.0109 | 1917.0030 | | | 26 | | | 0.035 | | K.HVLVILTDMSSYAEALR.E |
|  |  |  | 1177.1178 | 2352.2210 | 2352.2100 | | | 81 | | | 7.4e-008 | | K.AVVGEEALTSDDLLYLEFLQK.F |
|  |  |  | 803.7344 | 2408.1815 | 2408.1715 | | | 57 | | | 2.9e-005 | | R.IYPEEMIQTGISAIDGMNSIAR.G |
| V-type ATPase subunit B | P31408 | 324^b^ | **Observed** | **Mr(expt)** | **Mr(calc)** | | | **Score** | | | **Exp Value** | | **Peptide** |
|  |  |  | 545.7914 | 1089.5682 | 1089.5666 | | | 32 | | | 0.036 | | R.SGQVLEVSGSK.A |
|  |  |  | 552.7730 | 1103.5315 | 1103.5281 | | | 52 | | | 0.00024 | | R.TPVSEDMLGR.V |
|  |  |  | 654.8234 | 1307.6322 | 1307.6258 | | | 37 | | | 0.0072 | | R.NFIAQGPYENR.T |
|  |  |  | 719.3755 | 1436.7365 | 1436.7300 | | | 74 | | | 1.1e-006 | | R.IPQSTLSEFYPR.D |
|  |  |  | 760.9042 | 1519.7938 | 1519.7882 | | | 88 | | | 4.5e-008 | | K.AVVQVFEGTSGIDAK.K |
|  |  |  | 798.9619 | 1595.9093 | 1595.9035 | | | 45 | | | 0.00025 | | R.QIYPPINVLPSLSR.L |
|  |  |  | 853.9634 | 1705.9123 | 1705.9039 | | | 66 | | | 3.6e-006 | | R.TVYETLDIGWQLLR.I |
|  |  |  | 956.9470 | 1911.8795 | 1911.8713 | | | 25 | | | 0.05 | | R.GFPGYMYTDLATIYER.A |
|  |  |  | 640.0108 | 1917.0106 | 1917.0030 | | | 53 | | | 6.5e-005 | | K.HVLVILTDMSSYAEALR.E |
|  |  |  | 1177.1169 | 2352.2192 | 2352.2100 | | | 76 | | | 2.6e-007 | | K.AVVGEEALTSDDLLYLEFLQK.F |
|  |  |  | 998.4757 | 2992.4052 | 2992.3946 | | | 43 | | | 0.00035 | | K.SKDVVDYSEENFAIVFAAMGVNMETAR.F |
| V-type ATPase subunit E 1 | P11019 | 114^a^ | **Observed** | **Mr(expt)** | **Mr(calc)** | | | **Score** | | | **Exp Value** | | **Peptide** |
|  |  |  | 554.7636 | 1107.5126 | 1107.5084 | | | 13 | | | 0.17 | | K.AEEEFNIEK.G |
|  |  |  | 772.4051 | 1542.7957 | 1542.7898 | | | 46 | | | 7.1e-005 | | R.LDLIAQQMMPEVR.G |
|  |  |  | 793.9260 | 1574.7853 | 1585.8311 | | | 77 | | | 3.7e-008 | | R.ARDDLITDLLNEAK.Q |
| V-type ATPase subunit E 1 | P11019 | 117^b^ | **Observed** | **Mr(expt)** | **Mr(calc)** | | | **Score** | | | **Exp Value** | | **Peptide** |
|  |  |  | 495.7649 | 989.5153 | 989.5043 | | | 44 | | | 0.0008 | | R.GALFGANANR.K |
|  |  |  | 788.4051 | 1574.7956 | 1574.7796 | | | 58 | | | 1.7e-005 | | R.LDLIAQQMMPEVR.G |
|  |  |  | 793.9330 | 1585.8514 | 1585.8311 | | | 63 | | | 3.8e-006 | | R.ARDDLITDLLNEAK.Q |
|  |  |  | 537.2444 | 1608.7113 | 1608.6912 | | | 34 | | | 0.0012 | | K.HMMAFIEQEANEK.A |
| Actin, cytoplasmic | Q4L0Y2 | 231^a^ | **Observed** | **Mr(expt)** | **Mr(calc)** | | | **Score** | | | **Exp Value** | | **Peptide** |
|  |  |  | 488.7293 | 975.4440 | 975.4410 | | | 66 | | | 8.4e-006 | | K.AGFAGDDAPR.A |
|  |  |  | 499.7492 | 997.4838 | 997.4790 | | | 44 | | | 0.0014 | | R.DLTDYLMK.I |
|  |  |  | 566.7689 | 1131.5232 | 1131.5197 | | | 46 | | | 0.00068 | | R.GYSFTTTAER.E |
|  |  |  | 581.3147 | 1160.6148 | 1160.6111 | | | 48 | | | 0.0008 | | K.EITALAPSTMK.I |
|  |  |  | 586.2905 | 1170.5664 | 1170.5638 | | | 42 | | | 0.0019 | | R.HQGVMVGMGQK.D |
|  |  |  | 589.3118 | 1176.6091 | 1176.6060 | | | 31 | | | 0.042 | | K.EITALAPSTMK.I |
|  |  |  | 506.2407 | 1515.7002 | 1515.6954 | | | 29 | | | 0.029 | | K.QEYDESGPSIVHR.K |
|  |  |  | 895.9535 | 1789.8925 | 1789.8846 | | | 60 | | | 2.1e-005 | | K.SYELPDGQVITIGNER.F |
|  |  |  | 1116.0411 | 2230.0676 | 2230.0576 | | | 80 | | | 1.7e-007 | | K.DLYANTVLSGGTTMYPGIADR.M |
| Actin, cytoplasmic | Q4L0Y2 | 244^b^ | **Observed** | **Mr(expt)** | **Mr(calc)** | | | **Score** | | | **Exp Value** | | **Peptide** |
|  |  |  | 473.2813 | 944.5479 | 944.5444 | | | 29 | | | 0.068 | | R.AVFPSIVGR.P |
|  |  |  | 488.7291 | 975.4437 | 975.4410 | | | 69 | | | 4.1e-006 | | K.AGFAGDDAPR.A |
|  |  |  | 566.7692 | 1131.5239 | 1131.5197 | | | 48 | | | 0.00039 | | R.GYSFTTTAER.E |
|  |  |  | 581.3149 | 1160.6153 | 1160.6111 | | | 38 | | | 0.0087 | | K.EITALAPSTMK.I |
|  |  |  | 589.3120 | 1176.6095 | 1176.6060 | | | 42 | | | 0.0032 | | K.EITALAPSTMK.I |
|  |  |  | 505.9231 | 1514.7476 | 1514.7419 | | | 31 | | | 0.026 | | K.IWHHTFYNELR.V |
|  |  |  | 895.9528 | 1789.8910 | 1789.8846 | | | 40 | | | 0.0021 | | K.SYELPDGQVITIGNER.F |
|  |  |  | 1116.0396 | 2230.0646 | 2230.0576 | | | 106 | | | 4e-010 | | K.DLYANTVLSGGTTMYPGIADR.M |
| Actin, cytoplasmic 1 | P29751 | 231^a^ | **Observed** | **Mr(expt)** | **Mr(calc)** | | | **Score** | | | **Exp Value** | | **Peptide** |
|  |  |  | 488.7293 | 975.4440 | 975.4410 | | | 66 | | | 8.4e-006 | | K.AGFAGDDAPR.A |
|  |  |  | 499.7492 | 997.4838 | 997.4790 | | | 44 | | | 0.0014 | | R.DLTDYLMK.I |
|  |  |  | 566.7689 | 1131.5232 | 1131.5197 | | | 46 | | | 0.00068 | | R.GYSFTTTAER.E |
|  |  |  | 581.3147 | 1160.6148 | 1160.6111 | | | 48 | | | 0.0008 | | K.EITALAPSTMK.I |
|  |  |  | 586.2905 | 1170.5664 | 1170.5638 | | | 42 | | | 0.0019 | | R.HQGVMVGMGQK.D |
|  |  |  | 895.9535 | 1789.8925 | 1789.8846 | | | 60 | | | 2.1e-005 | | K.SYELPDGQVITIGNER.F |
|  |  |  | 1116.0411 | 2230.0676 | 2230.0576 | | | 80 | | | 1.7e-007 | | K.DLYANTVLSGGTTMYPGIADR.M |
| Actin, cytoplasmic 1 | P29751 | 256^b^ | **Observed** | **Mr(expt)** | **Mr(calc)** | | | **Score** | | | **Exp Value** | | **Peptide** |
|  |  |  | 488.7339 | 975.4532 | 975.4410 | | | 50 | | | 0.00013 | | K.AGFAGDDAPR.A |
|  |  |  | 566.7740 | 1131.5334 | 1131.5197 | | | 56 | | | 2.7e-005 | | R.GYSFTTTAER.E |
|  |  |  | 581.3112 | 1160.6077 | 1160.6111 | | | 43 | | | 0.0011 | | K.EITALAPSTMK.I |
|  |  |  | 586.2954 | 1170.5763 | 1170.5638 | | | 28 | | | 0.019 | | R.HQGVMVGMGQK.D |
|  |  |  | 589.3173 | 1176.6201 | 1176.6060 | | | 39 | | | 0.0021 | | K.EITALAPSTMK.I |
|  |  |  | 505.9272 | 1514.7599 | 1514.7419 | | | 28 | | | 0.017 | | K.IWHHTFYNELR.V |
|  |  |  | 895.9607 | 1789.9069 | 1789.8846 | | | 66 | | | 1.7e-006 | | K.SYELPDGQVITIGNER.F |
|  |  |  | 1108.0521 | 2214.0896 | 2214.0627 | | | 116 | | | 1.2e-011 | | K.DLYANTVLSGGTTMYPGIADR.M |
| Vimentin | P48616 | 314^a^ | **Observed** | **Mr(expt)** | **Mr(calc)** | | | **Score** | | | **Exp Value** | | **Peptide** |
|  |  |  | 547.2695 | 1092.5245 | 1092.5200 | | | 65 | | | 7.7e-007 | | K.FADLSEAANR.N |
|  |  |  | 558.2903 | 1114.5661 | 1114.5618 | | | 41 | | | 0.00025 | | K.VELQELNDR.F |
|  |  |  | 585.3637 | 1168.7128 | 1168.7067 | | | 61 | | | 7.1e-007 | | K.ILLAELEQLK.G |
|  |  |  | 648.8097 | 1295.6049 | 1295.5993 | | | 38 | | | 0.00037 | | R.EEAESTLQSFR.Q |
|  |  |  | 655.3094 | 1308.6043 | 1308.5986 | | | 27 | | | 0.0019 | | K.NLQEAEEWYK.S |
|  |  |  | 656.3373 | 1310.6601 | 1310.6540 | | | 50 | | | 3.3e-005 | | K.MALDIEIATYR.K |
|  |  |  | 511.9582 | 1532.8528 | 1532.8450 | | | 41 | | | 0.0001 | | R.KVESLQEEIAFLK.K |
|  |  |  | 568.9477 | 1703.8212 | 1703.8148 | | | 26 | | | 0.0046 | | R.VEVERDNLAEDIMR.L |
|  |  |  | 1063.5410 | 2125.0674 | 2125.0579 | | | 113 | | | 6.8e-012 | | R.LLQDSVDFSLADAINTEFK.N |
| Vimentin | P48616 | 468^b^ | **Observed** | **Mr(expt)** | **Mr(calc)** | | | **Score** | | | **Exp Value** | | **Peptide** |
|  |  |  | 435.7264 | 869.4382 | 869.4283 | | | 34 | | | 0.013 | | R.FANYIDK.V |
|  |  |  | 531.7730 | 1061.5315 | 1061.5175 | | | 36 | | | 0.0036 | | K.LQEEMLQR.E |
|  |  |  | 546.7596 | 1091.5047 | 1091.4917 | | | 63 | | | 5.2e-006 | | R.DNLAEDIMR.L |
|  |  |  | 547.2733 | 1092.5321 | 1092.5200 | | | 62 | | | 9.1e-006 | | K.FADLSEAANR.N |
|  |  |  | 558.2945 | 1114.5744 | 1114.5618 | | | 54 | | | 6.1e-005 | | K.VELQELNDR.F |
|  |  |  | 559.7859 | 1117.5573 | 1117.5437 | | | 50 | | | 0.00013 | | R.LQDEIQNMK.E |
|  |  |  | 585.3685 | 1168.7223 | 1168.7067 | | | 60 | | | 2e-006 | | K.ILLAELEQLK.G |
|  |  |  | 627.7950 | 1253.5755 | 1253.5598 | | | 65 | | | 2.7e-006 | | R.LGDLYEEEMR.E |
|  |  |  | 656.3424 | 1310.6702 | 1310.6540 | | | 72 | | | 7.7e-007 | | K.MALDIEIATYR.K |
|  |  |  | 703.3905 | 1404.7665 | 1404.7500 | | | 41 | | | 0.0007 | | K.VESLQEEIAFLK.K |
|  |  |  | 511.9618 | 1532.8635 | 1532.8450 | | | 40 | | | 0.00039 | | R.KVESLQEEIAFLK.K |
|  |  |  | 563.6208 | 1687.8406 | 1687.8199 | | | 34 | | | 0.02 | | R.VEVERDNLAEDIMR.L |
|  |  |  | 1063.5504 | 2125.0862 | 2125.0579 | | | 83 | | | 2.4e-008 | | R.LLQDSVDFSLADAINTEFK.N |
| Cofilin-2 | Q148F1 | 88^a^ | **Observed** | **Mr(expt)** | **Mr(calc)** | | | **Score** | | | **Exp Value** | | **Peptide** |
|  |  |  | 669.3195 | 1336.6244 | 1336.6187 | | | 51 | | | 2.3e-005 | | R.YALYDATYETK.E |
|  |  |  | 525.2779 | 1048.5413 | 1048.5400 | | | 64 | | | 1.1e-005 | | R.KSSTQEEIK.K |
| Cofilin-2 | Q148F1 | 92^b^ | **Observed** | **Mr(expt)** | **Mr(calc)** | | | **Score** | | | **Exp Value** | | **Peptide** |
|  |  |  | 669.3210 | 1336.6259 | 1336.6187 | | | 57 | | | 3.3e-006 | | R.YALYDATYETK.E |
|  |  |  | 525.2776 | 1048.5406 | 1048.5400 | | | 71 | | | 2.3e-006 | | R.KSSTQEEIK.K |
| Elongation factor 1-alpha 1 | A2Q0Z0 | 164^a^ | **Observed** | **Mr(expt)** | **Mr(calc)** | | | **Score** | | | **Exp Value** | | **Peptide** |
|  |  |  | 488.2796 | 974.5447 | 974.5437 | | | 29 | | | 0.0033 | | R.LPLQDVYK.I |
|  |  |  | 513.3107 | 1024.6069 | 1024.6030 | | | 74 | | | 7.2e-008 | | K.IGGIGTVPVGR.V |
|  |  |  | 468.9157 | 1403.7251 | 1403.7197 | | | 40 | | | 0.00026 | | K.YYVTIIDAPGHR.D |
|  |  |  | 844.4670 | 2530.3791 | 2530.3717 | | | 31 | | | 0.00087 | | R.VETGVLKPGMVVTFAPVNVTTEVK.S |
| Elongation factor 1-alpha 1 | A2Q0Z0 | 109^b^ | **Observed** | **Mr(expt)** | **Mr(calc)** | | | **Score** | | | **Exp Value** | | **Peptide** |
|  |  |  | 488.2839 | 974.5533 | 974.5437 | | | 35 | | | 0.0039 | | R.LPLQDVYK.I |
|  |  |  | 513.3145 | 1024.6144 | 1024.6030 | | | 65 | | | 2e-006 | | K.IGGIGTVPVGR.V |
|  |  |  | 560.8108 | 1119.6071 | 1119.5924 | | | 34 | | | 0.0042 | | K.STTTGHLIYK.C |
|  |  |  | 657.8818 | 1313.7491 | 1313.7343 | | | 42 | | | 0.00055 | | R.EHALLAYTLGVK.Q |
|  |  |  | 468.9192 | 1403.7359 | 1403.7197 | | | 30 | | | 0.012 | | K.YYVTIIDAPGHR.D |
|  |  |  | 844.4746 | 2530.4021 | 2530.3717 | | | 31 | | | 0.00075 | | R.VETGVLKPGMVVTFAPVNVTTEVK.S |
| Guanine nucleotide-binding protein G(I)/G(S)/G(T) subunit β2 | P11017 | 66^a^ | **Observed** | **Mr(expt)** | **Mr(calc)** | | | **Score** | | | **Exp Value** | | **Peptide** |
|  |  |  | 403.2608 | 804.5070 | 804.4970 | | | 28 | | | 0.0057 | | K.VHAIPLR.S |
|  |  |  | 509.2887 | 1016.5629 | 1016.5502 | | | 37 | | | 0.0031 | | R.LLVSASQDGK.L |
|  |  |  | 677.3642 | 1352.7139 | 1352.6976 | | | 57 | | | 1.6e-005 | | K.LIIWDSYTTNK.V |
| Guanine nucleotide-binding protein G(I)/G(S)/G(T) subunit β2 | P11017 | 71^b^ | **Observed** | **Mr(expt)** | **Mr(calc)** | | | **Score** | | | **Exp Value** | | **Peptide** |
|  |  |  | 509.2842 | 1016.5539 | 1016.5502 | | | 51 | | | 0.00043 | | R.LLVSASQDGK.L |
|  |  |  | 677.3591 | 1352.7036 | 1352.6976 | | | 46 | | | 0.00093 | | K.LIIWDSYTTNK.V |
| 14-3-3ζ/δ | Q5R651^c^ | 101^b^ | **Observed** | **Mr(expt)** | **Mr(calc)** | | | **Score** | | | **Exp Value** | | **Peptide** |
|  |  |  | 640.3320 | 1278.6494 | 1278.6456 | | | 46 | | | 0.00098 | | R.YLAEVAAGDDKK.G |
|  |  |  | 774.8624 | 1547.7102 | 1547.7063 | | | 86 | | | 4.4e-008 | | K.SVTEQGAELSNEER.N |
| 14-3-3θ | Q0VC36^d^ | 94^a^ | **Observed** | **Mr(expt)** | **Mr(calc)** | | | **Score** | | | **Exp Value** | | **Peptide** |
|  |  |  | 454.2678 | 906.5211 | 906.5174 | | | 42 | | | 9.3e-005 | | R.NLLSVAYK.N |
|  |  |  | 595.3366 | 1188.6586 | 1188.6536 | | | 75 | | | 8.8e-008 | | K.DSTLIMQLLR.D |
| 14-3-3γ | P68253^e^ | 86^a^ | **Observed** | **Mr(expt)** | **Mr(calc)** | | | **Score** | | | **Exp Value** | | **Peptide** |
|  |  |  | 595.3424 | 1188.6702 | 1188.6536 | | | 47 | | | 0.00017 | | K.DSTLIMQLLR.D |
|  |  |  | 822.4063 | 1642.7981 | 1642.7798 | | | 68 | | | 1.2e-006 | | K.NVTELNEPLSNEER.N |
| 14-3-3γ | P68253^e^ | 111^b^ | **Observed** | **Mr(expt)** | **Mr(calc)** | | | **Score** | | | **Exp Value** | | **Peptide** |
|  |  |  | 595.3421 | 1188.6698 | 1188.6536 | | | 53 | | | 7e-005 | | K.DSTLIMQLLR.D |
|  |  |  | 822.4059 | 1642.7975 | 1642.7798 | | | 86 | | | 4.9e-008 | | K.NVTELNEPLSNEER.N |
| 40S ribosomal protein S10 | Q3T0F4 | 117^a^ | **Observed** | **Mr(expt)** | **Mr(calc)** | | | **Score** | | | **Exp Value** | | **Peptide** |
|  |  |  | 555.3372 | 1108.6598 | 1108.6532 | | | 58 | | | 1.7e-006 | | R.IAIYELLFK.E |
|  |  |  | 785.3831 | 1568.7516 | 1568.7583 | | | 80 | | | 4.4e-008 | | K.KAEAGAGSATEFQFR.G |
| 40S ribosomal protein S10 | Q3T0F4 | 97^b^ | **Observed** | **Mr(expt)** | **Mr(calc)** | | | **Score** | | | **Exp Value** | | **Peptide** |
|  |  |  | 555.3362 | 1108.6579 | 1108.6532 | | | 47 | | | 0.00018 | | R.IAIYELLFK.E |
|  |  |  | 785.3830 | 1568.7513 | 1568.7583 | | | 76 | | | 3.7e-008 | | K.KAEAGAGSATEFQFR.G |
| 40S ribosomal protein S18 | Q3T0R1 | 107^a^ | **Observed** | **Mr(expt)** | **Mr(calc)** | | | **Score** | | | **Exp Value** | | **Peptide** |
|  |  |  | 474.3019 | 946.5893 | 946.5851 | | | 62 | | | 6.4e-006 | | K.IAFAITAIK.G |
|  |  |  | 501.2744 | 1000.5342 | 1000.5302 | | | 49 | | | 0.00065 | | R.VLNTNIDGR.R |
|  |  |  | 530.7844 | 1059.5543 | 1059.5502 | | | 38 | | | 0.0097 | | K.IPDWFLNR.Q |
|  |  |  | 661.3409 | 1320.6672 | 1320.6674 | | | 58 | | | 5.7e-005 | | K.YSQVLANGLDNK.L |
| 40S ribosomal protein S18 | Q3T0R1 | 88^b^ | **Observed** | **Mr(expt)** | **Mr(calc)** | | | **Score** | | | **Exp Value** | | **Peptide** |
|  |  |  | 474.3018 | 946.5891 | 946.5851 | | | 44 | | | 3.7e-005 | | K.IAFAITAIK.G |
|  |  |  | 530.7850 | 1059.5554 | 1059.5502 | | | 42 | | | 0.00023 | | K.IPDWFLNR.Q |
|  |  |  | 661.3424 | 1320.6702 | 1320.6674 | | | 48 | | | 4.3e-005 | | K.YSQVLANGLDNK.L |
| Peroxiredoxin-1 | Q6B4U9 | 191^a^ | **Observed** | **Mr(expt)** | **Mr(calc)** | | | **Score** | | | **Exp Value** | | **Peptide** |
|  |  |  | 447.7211 | 893.4277 | 893.4243 | | | 46 | | | 0.0011 | | K.ADEGISFR.G |
|  |  |  | 460.7598 | 919.5051 | 919.5015 | | | 40 | | | 0.0055 | | R.GLFIIDDK.G |
|  |  |  | 470.7368 | 939.4590 | 939.4549 | | | 38 | | | 0.0072 | | K.DISLSDYK.G |
|  |  |  | 554.3080 | 1106.6015 | 1106.5972 | | | 45 | | | 0.0011 | | R.TIAQDYGVLK.A |
|  |  |  | 598.8217 | 1195.6288 | 1195.6237 | | | 56 | | | 7.3e-005 | | R.LVQAFQFTDK.H |
|  |  |  | 606.3427 | 1210.6708 | 1210.6670 | | | 58 | | | 3.5e-005 | | R.QITVNDLPVGR.S |
|  |  |  | 453.9398 | 1358.7974 | 1358.7922 | | | 60 | | | 7.2e-006 | | R.GLFIIDDKGILR.Q |
|  |  |  | 811.9349 | 1621.8552 | 1621.8498 | | | 72 | | | 1.2e-006 | | K.QGGLGPMNIPLVSDPK.R |
| Peroxiredoxin-1 | Q6B4U9 | 107^b^ | **Observed** | **Mr(expt)** | **Mr(calc)** | | | **Score** | | | **Exp Value** | | **Peptide** |
|  |  |  | 460.7597 | 919.5048 | 919.5015 | | | 30 | | | 0.053 | | R.GLFIIDDK.G |
|  |  |  | 554.3079 | 1106.6012 | 1106.5972 | | | 42 | | | 0.0025 | | R.TIAQDYGVLK.A |
|  |  |  | 590.7888 | 1179.5631 | 1179.5594 | | | 43 | | | 0.0021 | | K.ATAVMPDGQFK.D |
|  |  |  | 598.8211 | 1195.6276 | 1195.6237 | | | 56 | | | 8.3e-005 | | R.LVQAFQFTDK.H |
|  |  |  | 606.3432 | 1210.6719 | 1210.6670 | | | 42 | | | 0.0013 | | R.QITVNDLPVGR.S |
|  |  |  | 819.9320 | 1637.8494 | 1637.8447 | | | 51 | | | 0.00018 | | K.QGGLGPMNIPLVSDPK.R |
| 40S ribosomal protein S15a | Q76I82 | 79^a^ | **Observed** | **Mr(expt)** | **Mr(calc)** | | | **Score** | | | **Exp Value** | | **Peptide** |
|  |  |  | 375.2143 | 748.4141 | 748.4119 | | | 26 | | | 0.018 | | R.FDVQLK.D |
|  |  |  | 436.2732 | 870.5319 | 870.5287 | | | 41 | | | 0.00028 | | K.IVVNLTGR.L |
|  |  |  | 495.7700 | 989.5255 | 989.5215 | | | 53 | | | 3.1e-005 | | R.MNVLADALK.S |
|  |  |  | 567.6084 | 1699.8034 | 1699.7954 | | | 33 | | | 0.0005 | | K.HGYIGEFEIIDDHR.A |
| 40S ribosomal protein S15a | Q76I82 | 85^b^ | **Observed** | **Mr(expt)** | **Mr(calc)** | | | **Score** | | | **Exp Value** | | **Peptide** |
|  |  |  | 375.2147 | 748.4148 | 748.4119 | | | 33 | | | 0.032 | | R.FDVQLK.D |
|  |  |  | 436.2729 | 870.5312 | 870.5287 | | | 60 | | | 4.1e-005 | | K.IVVNLTGR.L |
|  |  |  | 495.7699 | 989.5253 | 989.5215 | | | 61 | | | 4.7e-005 | | R.MNVLADALK.S |
| 40S ribosomal protein S13 | Q56JX8 | 79^a^ | **Observed** | **Mr(expt)** | **Mr(calc)** | | | **Score** | | | **Exp Value** | | **Peptide** |
|  |  |  | 422.2700 | 842.5255 | 842.5225 | | | 37 | | | 0.0037 | | R.LILIESR.I |
|  |  |  | 546.2986 | 1090.5826 | 1090.5771 | | | 45 | | | 0.0018 | | K.GLSQSALPYR.R |
|  |  |  | 627.3852 | 1252.7558 | 1252.7503 | | | 41 | | | 0.00068 | | K.GLTPSQIGVILR.D |
| 40S ribosomal protein S13 | Q56JX8 | 88^b^ | **Observed** | **Mr(expt)** | **Mr(calc)** | | | **Score** | | | **Exp Value** | | **Peptide** |
|  |  |  | 422.2700 | 842.5254 | 842.5225 | | | 28 | | | 0.0043 | | R.LILIESR.I |
|  |  |  | 546.2999 | 1090.5852 | 1090.5771 | | | 32 | | | 0.002 | | K.GLSQSALPYR.R |
|  |  |  | 627.3850 | 1252.7555 | 1252.7503 | | | 44 | | | 4.6e-005 | | K.GLTPSQIGVILR.D |
| 60S acidic ribosomal protein P2 | P42899 | 121^a^ | **Observed** | **Mr(expt)** | **Mr(calc)** | | | **Score** | | | **Exp Value** | | **Peptide** |
|  |  |  | 628.8488 | 1255.6829 | 1255.6772 | | | 72 | | | 1.9e-006 | | K.NIEDVIAQGIGK.L |
|  |  |  | 983.3849 | 1964.7553 | 1964.7469 | | | 76 | | | 3.7e-008 | | K.KEESEESDDDMGFGLFD.- |
| 60S acidic ribosomal protein P2 | P42899 | 101^b^ | **Observed** | **Mr(expt)** | **Mr(calc)** | | | **Score** | | | **Exp Value** | | **Peptide** |
|  |  |  | 628.8538 | 1255.6931 | 1255.6772 | | | 68 | | | 1.6e-006 | | K.NIEDVIAQGIGK.L |
|  |  |  | 983.3846 | 1964.7548 | 1964.7469 | | | 51 | | | 8.2e-005 | | K.KEESEESDDDMGFGLFD |
| 60S ribosomal protein L18 | Q5E973 | 180^a^ | **Observed** | **Mr(expt)** | **Mr(calc)** | | | **Score** | | | **Exp Value** | | **Peptide** |
|  |  |  | 625.8429 | 1249.6713 | 1249.6667 | | | 54 | | | 1e-005 | | R.TNSTFNQVVLK.R |
|  |  |  | 673.3719 | 1344.7292 | 1344.7249 | | | 84 | | | 9.5e-009 | | K.TAVVVGTITDDVR.V |
|  |  |  | 730.9068 | 1459.7991 | 1459.7922 | | | 87 | | | 2.9e-009 | | K.ILTFDQLALDSPK.G |
| 60S ribosomal protein L18 | Q5E973 | 174^b^ | **Observed** | **Mr(expt)** | **Mr(calc)** | | | **Score** | | | **Exp Value** | | **Peptide** |
|  |  |  | 625.8432 | 1249.6719 | 1249.6667 | | | 43 | | | 0.00014 | | R.TNSTFNQVVLK.R |
|  |  |  | 673.3729 | 1344.7313 | 1344.7249 | | | 83 | | | 1.3e-008 | | K.TAVVVGTITDDVR.V |
|  |  |  | 730.9076 | 1459.8006 | 1459.7922 | | | 94 | | | 5.7e-010 | | K.ILTFDQLALDSPK.G |
| 60S ribosomal protein L10a (Fragment) | P53027 | 107^a^ | **Observed** | **Mr(expt)** | **Mr(calc)** | | | **Score** | | | **Exp Value** | | **Peptide** |
|  |  |  | 742.9063 | 1483.7980 | 1483.7922 | | | 72 | | | 1.5e-006 | | K.KYDAFLASESLIK.Q |
|  |  |  | 483.7502 | 965.4858 | 965.4818 | | | 67 | | | 5.5e-006 | | R.DTLYEAVR.E |
| 60S ribosomal protein L10a (Fragment) | P53027 | 111^b^ | **Observed** | **Mr(expt)** | **Mr(calc)** | | | **Score** | | | **Exp Value** | | **Peptide** |
|  |  |  | 742.9062 | 1483.7978 | 1483.7922 | | | 58 | | | 4.3e-005 | | K.KYDAFLASESLIK.Q |
|  |  |  | 483.7500 | 965.4857 | 965.4818 | | | 80 | | | 4.6e-008 | | R.DTLYEAVR.E |
| 60S ribosomal protein L13a | Q3SZ90 | 93^a^ | **Observed** | **Mr(expt)** | **Mr(calc)** | | | **Score** | | | **Exp Value** | | **Peptide** |
|  |  |  | 578.8226 | 1155.6307 | 1155.6248 | | | 43 | | | 0.00015 | | M.AEGQVLVLDGR.G |
|  |  |  | 626.8276 | 1251.6406 | 1251.6347 | | | 55 | | | 7.9e-006 | | K.YQAVTATLEEK.R |
| 60S ribosomal protein L13a | Q3SZ90 | 169^b^ | **Observed** | **Mr(expt)** | **Mr(calc)** | | | **Score** | | | **Exp Value** | | **Peptide** |
|  |  |  | 391.7327 | 781.4508 | 781.4486 | | | 28 | | | 0.0037 | | K.YLAFLR.K |
|  |  |  | 578.8215 | 1155.6284 | 1155.6248 | | | 55 | | | 8e-006 | | M.AEGQVLVLDGR.G |
|  |  |  | 626.8273 | 1251.6400 | 1251.6347 | | | 55 | | | 9e-006 | | K.YQAVTATLEEK.R |
| 60S ribosomal protein L35 | Q29361 | 110^a^ | **Observed** | **Mr(expt)** | **Mr(calc)** | | | **Score** | | | **Exp Value** | | **Peptide** |
|  |  |  | 572.3419 | 1142.6693 | 1142.6659 | | | 56 | | | 5.4e-005 | | R.VLTVINQTQK.E |
|  |  |  | 785.9474 | 1569.8802 | 1569.8726 | | | 80 | | | 2e-007 | | K.QLEDLKVELSQLR.V |
| 60S ribosomal protein L35 | Q29361 | 100^b^ | **Observed** | **Mr(expt)** | **Mr(calc)** | | | **Score** | | | **Exp Value** | | **Peptide** |
|  |  |  | 572.3465 | 1142.6785 | 1142.6659 | | | 56 | | | 5.4e-005 | | R.VLTVINQTQK.E |
|  |  |  | 785.9470 | 1569.8795 | 1569.8726 | | | 72 | | | 2.1e-006 | | K.QLEDLKVELSQLR.V |
| Exosome complex component RRP40 | Q3T0E1 | 125^a^ | **Observed** | **Mr(expt)** | **Mr(calc)** | | | **Score** | | | **Exp Value** | | **Peptide** |
|  |  |  | 386.2581 | 771.5089 | 771.5065 | | | 89 | | | 1.4e-009 | | K.VTLGLIR.K |
|  |  |  | 584.5548 | 1169.3318 | 1169.3302 | | | 63 | | | 1.6e-005 | | R.TVLDQVVLPGE.E |
| Exosome complex component RRP40 | Q3T0E1 | 123^b^ | **Observed** | **Mr(expt)** | **Mr(calc)** | | | **Score** | | | **Exp Value** | | **Peptide** |
|  |  |  | 386.2605 | 771.5109 | 771.5065 | | | 80 | | | 2.2e-008 | | K.VTLGLIR.K |
|  |  |  | 584.2891 | 1169.3313 | 1169.3302 | | | 50 | | | 8.3e-005 | | R.TVLDQVVLPGE.E |
| 60S ribosomal protein L13 | Q56JZ1 | 130^a^ | **Observed** | **Mr(expt)** | **Mr(calc)** | | | **Score** | | | **Exp Value** | | **Peptide** |
|  |  |  | 379.7519 | 757.4893 | 757.4850 | | | 30 | | | 0.002 | | K.LILFPR.K |
|  |  |  | 475.7527 | 949.4909 | 949.4869 | | | 34 | | | 0.0025 | | R.GFSLEELR.V |
|  |  |  | 479.2756 | 956.5365 | 956.5291 | | | 37 | | | 0.0007 | | R.TIGISVDPR.R |
|  |  |  | 595.3121 | 1188.6096 | 1188.6040 | | | 53 | | | 1.2e-005 | | R.VATWFNQPAR.K |
|  |  |  | 698.9054 | 1395.7963 | 1395.7908 | | | 51 | | | 8.7e-006 | | K.LATQLTGPVMPIR.N |
| 60S ribosomal protein L13 | Q56JZ1 | 124^b^ | **Observed** | **Mr(expt)** | **Mr(calc)** | | | **Score** | | | **Exp Value** | | **Peptide** |
|  |  |  | 379.7514 | 757.4883 | 757.4850 | | | 33 | | | 0.001 | | K.LILFPR.K |
|  |  |  | 475.7530 | 949.4915 | 949.4869 | | | 41 | | | 0.00053 | | R.GFSLEELR.V |
|  |  |  | 479.2736 | 956.5327 | 956.5291 | | | 31 | | | 0.0026 | | R.TIGISVDPR.R |
|  |  |  | 595.3122 | 1188.6099 | 1188.6040 | | | 58 | | | 3.8e-006 | | R.VATWFNQPAR.K |
|  |  |  | 698.9059 | 1395.7972 | 1395.7908 | | | 49 | | | 1.2e-005 | | K.LATQLTGPVMPIR.N |
| Ubiquitin-60s ribosomal protein L40 | P63050 | 123^a^ | **Observed** | **Mr(expt)** | **Mr(calc)** | | | **Score** | | | **Exp Value** | | **Peptide** |
|  |  |  | 894.4705 | 1786.9264 | 1786.9200 | | | 65 | | | 1e-006 | | K.TITLEVEPSDTIENVK.A |
|  |  |  | 541.2819 | 1080.5492 | 1080.5451 | | | 80 | | | 4.5e-008 | | R.TLSDYNIQK.E |
| Ubiquitin-60s ribosomal protein L40 | P63050 | 111^b^ | **Observed** | **Mr(expt)** | **Mr(calc)** | | | **Score** | | | **Exp Value** | | **Peptide** |
|  |  |  | 894.4779 | 1786.9412 | 1786.9200 | | | 92 | | | 6.6e-010 | | K.TITLEVEPSDTIENVK.A |
|  |  |  | 541.2820 | 1080.5493 | 1080.5451 | | | 43 | | | 0.00034 | | R.TLSDYNIQK.E |
| Heat shock cognate 71 kDa protein | A2Q0Z1 | 228^a^ | **Observed** | **Mr(expt)** | **Mr(calc)** | | | **Score** | | | **Exp Value** | | **Peptide** |
|  |  |  | 652.3055 | 1302.5965 | 1302.5914 | | | 73 | | | 1e-007 | | K.NSLESYAFNMK.A |
|  |  |  | 744.3570 | 1486.6994 | 1486.6940 | | | 72 | | | 1.2e-007 | | R.TTPSYVAFTDTER.L |
|  |  |  | 833.4014 | 1664.7882 | 1664.7828 | | | 62 | | | 1.5e-006 | | K.NQVAMNPTNTVFDAK.R |
|  |  |  | 991.5057 | 1980.9968 | 1980.9905 | | | 48 | | | 1.7e-005 | | K.TVTNAVVTVPAYFNDSQR.Q |
| Heat shock cognate 71 kDa protein | A2Q0Z1 | 219^b^ | **Observed** | **Mr(expt)** | **Mr(calc)** | | | **Score** | | | **Exp Value** | | **Peptide** |
|  |  |  | 614.8198 | 1227.6251 | 1227.6207 | | | 53 | | | 0.00018 | | K.VEIIANDQGNR.T |
|  |  |  | 652.3062 | 1302.5977 | 1302.5914 | | | 41 | | | 0.0021 | | K.NSLESYAFNMK.A |
|  |  |  | 744.3576 | 1486.7007 | 1486.6940 | | | 78 | | | 3.8e-007 | | R.TTPSYVAFTDTER.L |
|  |  |  | 816.8984 | 1631.7823 | 1631.7753 | | | 69 | | | 3.3e-006 | | K.SFYPEEVSSMVLTK.M |
|  |  |  | 830.4538 | 1658.8931 | 1658.8879 | | | 63 | | | 1.2e-005 | | R.IINEPTAAAIAYGLDK.K |
|  |  |  | 833.4017 | 1664.7889 | 1664.7828 | | | 60 | | | 2.4e-005 | | K.NQVAMNPTNTVFDAK.R |
|  |  |  | 991.5062 | 1980.9978 | 1980.9905 | | | 39 | | | 0.0023 | | K.TVTNAVVTVPAYFNDSQR.Q |
| Alpha-actinin-1 | Q3B7N2 | 142^a^ | **Observed** | **Mr(expt)** | **Mr(calc)** | | | **Score** | | | **Exp Value** | | **Peptide** |
|  |  |  | 374.7205 | 747.4264 | 747.4167 | | | 25 | | | 0.055 | | K.YLDIPK.M |
|  |  |  | 608.3488 | 1214.6831 | 1214.6659 | | | 74 | | | 4e-007 | | K.LASDLLEWIR.R |
|  |  |  | 693.9002 | 1385.7858 | 1385.7667 | | | 66 | | | 1.8e-006 | | R.VGWEQLLTTIAR.T |
|  |  |  | 769.3999 | 1536.7853 | 1536.7671 | | | 61 | | | 9.3e-006 | | R.FAIQDISVEETSAK.E |
| Alpha-actinin-1 | Q3B7N2 | 92^b^ | **Observed** | **Mr(expt)** | **Mr(calc)** | | | **Score** | | | **Exp Value** | | **Peptide** |
|  |  |  | 693.8933 | 1385.7721 | 1385.7667 | | | 65 | | | 5.8e-006 | | R.VGWEQLLTTIAR.T |
|  |  |  | 608.3524 | 1214.6903 | 1214.6659 | | | 60 | | | 3.1e-005 | | K.LASDLLEWIR.R |
| Voltage-dependent anion-selective channel protein 1 | Q9MZ16 | 397^a^ | **Observed** | **Mr(expt)** | **Mr(calc)** | | | **Score** | | | **Exp Value** | | **Peptide** |
|  |  |  | 474.2658 | 946.5170 | 946.5124 | | | 45 | | | 0.0018 | | K.LGLGLEFQA.- |
|  |  |  | 515.8131 | 1029.6116 | 1029.6070 | | | 70 | | | 2.2e-006 | | K.LTLSALLDGK.N |
|  |  |  | 607.3164 | 1212.6182 | 1212.6139 | | | 67 | | | 7.2e-006 | | R.VTQSNFAVGYK.T |
|  |  |  | 687.8352 | 1373.6559 | 1373.6503 | | | 65 | | | 8.5e-006 | | R.WTEYGLTFTEK.W |
|  |  |  | 700.8407 | 1399.6669 | 1399.6620 | | | 82 | | | 1.7e-007 | | K.LTFDSSFSPNTGK.K |
|  |  |  | 980.4449 | 1958.8752 | 1958.8705 | | | 110 | | | 1e-010 | | K.SENGLEFTSSGSANTETTK.V |
|  |  |  | 701.7258 | 2102.1555 | 2102.1735 | | | 33 | | | 0.0025 | | K.VNNSSLIGLGYTQTLKPGIK.L |
|  |  |  | 1088.5336 | 2175.0526 | 2175.0444 | | | 112 | | | 1.1e-010 | | K.WNTDNTLGTEITVEDQLAR.G |
|  |  |  | 867.4053 | 2599.1939 | 2599.1827 | | | 59 | | | 7.9e-006 | | K.TDEFQLHTNVNDGTEFGGSIYQK.V |
| Voltage-dependent anion-selective channel protein 1 | Q9MZ16 | 316^b^ | **Observed** | **Mr(expt)** | **Mr(calc)** | | | **Score** | | | **Exp Value** | | **Peptide** |
|  |  |  | 515.8127 | 1029.6109 | 1029.6070 | | | 72 | | | 1.3e-006 | | K.LTLSALLDGK.N |
|  |  |  | 607.3162 | 1212.6179 | 1212.6139 | | | 64 | | | 1.5e-005 | | R.VTQSNFAVGYK.T |
|  |  |  | 687.8352 | 1373.6559 | 1373.6503 | | | 52 | | | 0.00016 | | R.WTEYGLTFTEK.W |
|  |  |  | 700.8408 | 1399.6671 | 1399.6620 | | | 78 | | | 4.5e-007 | | K.LTFDSSFSPNTGK.K |
|  |  |  | 701.7345 | 2102.1816 | 2102.1735 | | | 44 | | | 8.8e-005 | | K.VNNSSLIGLGYTQTLKPGIK.L |
|  |  |  | 1088.5341 | 2175.0536 | 2175.0444 | | | 115 | | | 5.8e-011 | | K.WNTDNTLGTEITVEDQLAR.G |
| Voltage-dependent anion-selective channel protein 2 | Q9MZ15 | 236^a^ | **Observed** | **Mr(expt)** | **Mr(calc)** | | | **Score** | | | **Exp Value** | | **Peptide** |
|  |  |  | 412.7385 | 823.4624 | 823.4592 | | | 27 | | | 0.0073 | | K.GFGFGLVK.L |
|  |  |  | 470.7373 | 939.4601 | 939.4563 | | | 33 | | | 0.0015 | | R.NNFAVGYR.T |
|  |  |  | 508.8052 | 1015.5958 | 1015.5914 | | | 74 | | | 7.3e-008 | | K.LTLSALVDGK.S |
|  |  |  | 647.3408 | 1292.6671 | 1292.6612 | | | 48 | | | 4.6e-005 | | K.YQLDPTASISAK.V |
|  |  |  | 714.8564 | 1427.6983 | 1427.6933 | | | 82 | | | 1.4e-008 | | K.LTFDTTFSPNTGK.K |
|  |  |  | 701.7262 | 2102.1568 | 2102.1484 | | | 26 | | | 0.0023 | | K.VNNSSLIGVGYTQTLRPGVK.L |
|  |  |  | 843.3977 | 2527.1712 | 2527.1616 | | | 80 | | | 9.9e-009 | | R.TGDFQLHTNVNDGTEFGGSIYQK.V |
| Voltage-dependent anion-selective channel protein 2 | Q9MZ15 | 157^b^ | **Observed** | **Mr(expt)** | **Mr(calc)** | | | **Score** | | | **Exp Value** | | **Peptide** |
|  |  |  | 412.7386 | 823.4626 | 823.4592 | | | 27 | | | 0.0087 | | K.GFGFGLVK.L |
|  |  |  | 470.7372 | 939.4598 | 939.4563 | | | 40 | | | 0.00029 | | R.NNFAVGYR.T |
|  |  |  | 508.8051 | 1015.5957 | 1015.5914 | | | 60 | | | 1.6e-006 | | K.LTLSALVDGK.S |
|  |  |  | 647.3410 | 1292.6675 | 1292.6612 | | | 39 | | | 0.00041 | | K.YQLDPTASISAK.V |
|  |  |  | 714.8573 | 1427.7000 | 1427.6933 | | | 72 | | | 1.4e-007 | | K.LTFDTTFSPNTGK.K |
|  |  |  | 701.7262 | 2102.1568 | 2102.1484 | | | 35 | | | 0.00033 | | K.VNNSSLIGVGYTQTLRPGVK.L |
| Dihydrolipoyllysine-residue succinyltransferase component of 2-oxoglutarate dehydrogenase complex | P11179 | 277^a^ | **Observed** | **Mr(expt)** | **Mr(calc)** | | | **Score** | | | **Exp Value** | | **Peptide** |
|  |  |  | 426.7886 | 851.5626 | 851.5593 | | | 24 | | | 0.0062 | | R.GLVVPVIR.N |
|  |  |  | 500.2721 | 998.5296 | 998.5259 | | | 60 | | | 3.9e-005 | | K.LGFMSAFVK.A |
|  |  |  | 595.3353 | 1188.6561 | 1188.6503 | | | 59 | | | 4.2e-005 | | K.VEGGTPLFTLR.K |
|  |  |  | 739.8621 | 1477.7096 | 1477.7049 | | | 107 | | | 5.3e-010 | | K.TPAFAESVTEGDVR.W |
|  |  |  | 641.3169 | 1920.9289 | 1920.9218 | | | 38 | | | 0.0029 | | K.TPAFAESVTEGDVRWEK.A |
|  |  |  | 660.6687 | 1978.9843 | 1978.9757 | | | 40 | | | 0.0018 | | K.VEVRPMMYVALTYDHR.L |
|  |  |  | 1094.0722 | 2186.1298 | 2186.1219 | | | 113 | | | 6.4e-011 | | K.ASAFALQEQPVVNAVIDDATK.E |
|  |  |  | 945.1660 | 2832.4762 | 2832.4658 | | | 36 | | | 0.0016 | | K.ASAFALQEQPVVNAVIDDATKEVVYR.D |
| Dihydrolipoyllysine-residue succinyltransferase component of 2-oxoglutarate dehydrogenase complex | P11179 | 208^b^ | **Observed** | **Mr(expt)** | **Mr(calc)** | | | **Score** | | | **Exp Value** | | **Peptide** |
|  |  |  | 426.7886 | 851.5627 | 851.5593 | | | 27 | | | 0.0032 | | R.GLVVPVIR.N |
|  |  |  | 500.2722 | 998.5299 | 998.5259 | | | 55 | | | 0.00011 | | K.LGFMSAFVK.A |
|  |  |  | 739.8617 | 1477.7088 | 1477.7049 | | | 87 | | | 5.5e-008 | | K.TPAFAESVTEGDVR.W |
|  |  |  | 641.3164 | 1920.9275 | 1920.9218 | | | 36 | | | 0.0041 | | K.TPAFAESVTEGDVRWEK.A |
|  |  |  | 671.3309 | 2010.9709 | 2010.9655 | | | 32 | | | 0.014 | | K.VEVRPMMYVALTYDHR.L |
|  |  |  | 1094.0722 | 2186.1298 | 2186.1219 | | | 88 | | | 2.1e-008 | | K.ASAFALQEQPVVNAVIDDATK.E |
| Stress-70 protein | Q3ZCH0 | 674^a^ | **Observed** | **Mr(expt)** | **Mr(calc)** | | | **Score** | | | **Exp Value** | | **Peptide** |
|  |  |  | 616.3374 | 1230.6602 | 1230.6568 | | | 79 | | | 5e-007 | | R.QAASSLQQASLK.L |
|  |  |  | 621.8463 | 1241.6781 | 1241.6728 | | | 57 | | | 7e-005 | | K.DAGQISGLNVLR.V |
|  |  |  | 645.8460 | 1289.6775 | 1289.6728 | | | 66 | | | 9.3e-006 | | K.VQQTVQDLFGR.A |
|  |  |  | 681.3781 | 1360.7417 | 1360.7351 | | | 58 | | | 3.4e-005 | | R.AQFEGIVTDLIR.R |
|  |  |  | 723.8876 | 1445.7607 | 1445.7548 | | | 106 | | | 7.7e-010 | | K.SDIGEVILVGGMTR.M |
|  |  |  | 777.4266 | 1552.8386 | 1552.8323 | | | 57 | | | 3.4e-005 | | K.LYSPSQIGAFVLMK.M |
|  |  |  | 784.8907 | 1567.7668 | 1567.7631 | | | 70 | | | 2.6e-006 | | R.QAVTNPNNTFYATK.R |
|  |  |  | 785.4242 | 1568.8339 | 1568.8272 | | | 55 | | | 7.2e-005 | | K.LYSPSQIGAFVLMK.M |
|  |  |  | 531.5973 | 1591.7700 | 1591.7664 | | | 36 | | | 0.0077 | | K.MKETAENYLGHTAK.N |
|  |  |  | 796.9828 | 1591.9511 | 1591.9450 | | | 44 | | | 6.8e-005 | | K.LLGQFTLIGIPPAPR.G |
|  |  |  | 823.4468 | 1644.8791 | 1644.8723 | | | 88 | | | 3.2e-008 | | R.VINEPTAAALAYGLDK.S |
|  |  |  | 853.9282 | 1705.8419 | 1705.8305 | | | 31 | | | 0.022 | | R.ETGVDLTKDNMALQR.V |
|  |  |  | 904.9572 | 1807.8999 | 1807.8952 | | | 68 | | | 3.6e-006 | | K.SQVFSTAADGQTQVEIK.V |
|  |  |  | 1028.4883 | 2054.9620 | 2054.9545 | | | 105 | | | 4.5e-010 | | K.STNGDTFLGGEDFDQALLR.H |
| Stress-70 protein | Q3ZCH0 | 561^b^ | **Observed** | **Mr(expt)** | **Mr(calc)** | | | **Score** | | | **Exp Value** | | **Peptide** |
|  |  |  | 621.8467 | 1241.6788 | 1241.6728 | | | 54 | | | 1.1e-005 | | K.DAGQISGLNVLR.V |
|  |  |  | 645.8467 | 1289.6789 | 1289.6728 | | | 60 | | | 3.8e-006 | | K.VQQTVQDLFGR.A |
|  |  |  | 681.3782 | 1360.7418 | 1360.7351 | | | 76 | | | 5.2e-008 | | R.AQFEGIVTDLIR.R |
|  |  |  | 723.8882 | 1445.7619 | 1445.7548 | | | 92 | | | 1.6e-009 | | K.SDIGEVILVGGMTR.M |
|  |  |  | 777.4274 | 1552.8401 | 1552.8323 | | | 45 | | | 5.5e-005 | | K.LYSPSQIGAFVLMK.M |
|  |  |  | 784.8919 | 1567.7692 | 1567.7631 | | | 67 | | | 4.6e-007 | | R.QAVTNPNNTFYATK.R |
|  |  |  | 823.4471 | 1644.8796 | 1644.8723 | | | 99 | | | 1.2e-010 | | R.VINEPTAAALAYGLDK.S |
|  |  |  | 847.9325 | 1693.8505 | 1693.8424 | | | 40 | | | 0.00022 | | K.NAVITVPAYFNDSQR.Q |
|  |  |  | 702.3661 | 2104.0766 | 2104.0688 | | | 15 | | | 0.031 | | R.VINEPTAAALAYGLDKSEDK.I |
|  |  |  | 812.0792 | 2433.2157 | 2433.2057 | | | 44 | | | 3.8e-005 | | R.EQQIVIQSSGGLSKDDIENMVK.N |
| Malate dehydrogenase^a^ | Q32LG3 | 155^a^ | **Observed** | **Mr(expt)** | **Mr(calc)** | | | **Score** | | | **Exp Value** | | **Peptide** |
|  |  |  | 537.2976 | 1072.5806 | 1072.5764 | | | 46 | | | 0.0015 | | R.IQEAGTEVVK.A |
|  |  |  | 557.8148 | 1113.6151 | 1113.6103 | | | 39 | | | 0.0053 | | K.MIAEAIPELK.A |
|  |  |  | 617.3660 | 1232.7175 | 1232.7129 | | | 54 | | | 4.7e-005 | | K.IFGVTTLDIVR.A |
|  |  |  | 664.3396 | 1326.6646 | 1326.6642 | | | 71 | | | 2.8e-006 | | R.FVFSLVDAMNGK.E |
|  |  |  | 727.8588 | 1453.7031 | 1453.6983 | | | 78 | | | 4e-007 | | K.AGAGSATLSMAYAGAR.F |
| Malate dehydrogenase | Q32LG3 | 134^b^ | **Observed** | **Mr(expt)** | **Mr(calc)** | | | **Score** | | | **Exp Value** | | **Peptide** |
|  |  |  | 557.8151 | 1113.6157 | 1113.6103 | | | 41 | | | 0.00025 | | K.MIAEAIPELK.A |
|  |  |  | 664.3406 | 1326.6666 | 1326.6642 | | | 63 | | | 1.2e-006 | | R.FVFSLVDAMNGK.E |
|  |  |  | 727.8599 | 1453.7052 | 1453.6983 | | | 75 | | | 3.7e-008 | | K.AGAGSATLSMAYAGAR.F |
| GrpE protein homolog 1 | Q3SZC1 | 73^a^ | **Observed** | **Mr(expt)** | **Mr(calc)** | | | **Score** | | | **Exp Value** | | **Peptide** |
|  |  |  | 501.7707 | 1001.5268 | 1001.5141 | | | 37 | | | 0.0034 | | R.ALADTENLR.Q |
|  |  |  | 629.3590 | 1256.7034 | 1256.6864 | | | 54 | | | 3.2e-005 | | K.DLLEVADILEK.A |
| GrpE protein homolog 1 | Q3SZC1 | 125^b^ | **Observed** | **Mr(expt)** | **Mr(calc)** | | | **Score** | | | **Exp Value** | | **Peptide** |
|  |  |  | 501.7700 | 1001.5259 | 1001.5141 | | | 68 | | | 2.1e-007 | | R.ALADTENLR.Q |
|  |  |  | 629.3585 | 1256.7025 | 1256.6864 | | | 78 | | | 2.4e-008 | | K.DLLEVADILEK.A |
| ADP/ATP translocase 3 | P32007 | 241^a^ | **Observed** | **Mr(expt)** | **Mr(calc)** | | | **Score** | | | **Exp Value** | | **Peptide** |
|  |  |  | 561.2936 | 1120.5727 | 1120.5665 | | | 46 | | | 0.0016 | | K.EQGVLSFWR.G |
|  |  |  | 610.3046 | 1218.5946 | 1218.5921 | | | 40 | | | 0.0037 | | R.AAYFGIYDTAK.G |
|  |  |  | 617.3480 | 1232.6814 | 1232.6765 | | | 79 | | | 3.7e-007 | | K.DFLAGGIAAAISK.T |
|  |  |  | 723.8776 | 1445.7406 | 1445.7343 | | | 57 | | | 6.5e-005 | | R.YFPTQALNFAFK.D |
|  |  |  | 814.4272 | 1626.8398 | 1626.8327 | | | 74 | | | 8.9e-007 | | R.GMGGAFVLVLYDELK.K |
|  |  |  | 1054.5571 | 3160.6495 | 3160.6380 | | | 32 | | | 0.0036 | | K.NTHIVVSWMIAQTVTAVAGVVSYPFDTVR.R |
| ADP/ATP translocase 3 | P32007 | 458^b^ | **Observed** | **Mr(expt)** | **Mr(calc)** | | | **Score** | | | **Exp Value** | | **Peptide** |
|  |  |  | 428.7605 | 855.5064 | 855.4926 | | | 41 | | | 0.0014 | | R.GNLANVIR.Y |
|  |  |  | 451.7514 | 901.4883 | 901.4770 | | | 28 | | | 0.035 | | K.GAWSNVLR.G |
|  |  |  | 561.2976 | 1120.5807 | 1120.5665 | | | 63 | | | 1.1e-005 | | K.EQGVLSFWR.G |
|  |  |  | 610.3099 | 1218.6053 | 1218.5921 | | | 37 | | | 0.0022 | | R.AAYFGIYDTAK.G |
|  |  |  | 617.3550 | 1232.6954 | 1232.6765 | | | 81 | | | 6.6e-008 | | K.DFLAGGIAAAISK.T |
|  |  |  | 723.8832 | 1445.7518 | 1445.7343 | | | 74 | | | 4.2e-007 | | R.YFPTQALNFAFK.D |
|  |  |  | 814.4327 | 1626.8508 | 1626.8327 | | | 80 | | | 9e-008 | | R.GMGGAFVLVLYDELK.K |
| ATP synthase subunit gamma | P05631 | 140^a^ | **Observed** | **Mr(expt)** | **Mr(calc)** | | | **Score** | | | **Exp Value** | | **Peptide** |
|  |  |  | 432.7522 | 863.4898 | 863.4865 | | | 25 | | | 0.13 | | K.LTLTFNR.T |
|  |  |  | 548.8105 | 1095.6064 | 1095.6037 | | | 41 | | | 0.002 | | K.HLIIGVSSDR.G |
|  |  |  | 649.8558 | 1297.6970 | 1297.6918 | | | 83 | | | 1.5e-007 | | R.VYGVGSLALYEK.A |
|  |  |  | 657.8641 | 1313.7136 | 1313.7078 | | | 65 | | | 1.3e-005 | | K.ELIEIISGAAALD.- |
|  |  |  | 584.9721 | 1751.8944 | 1751.8876 | | | 31 | | | 0.019 | | K.NASEMIDKLTLTFNR.T |
| ATP synthase subunit gamma | P05631 | 217^b^ | **Observed** | **Mr(expt)** | **Mr(calc)** | | | **Score** | | | **Exp Value** | | **Peptide** |
|  |  |  | 432.7523 | 863.4900 | 863.4865 | | | 22 | | | 0.015 | | K.LTLTFNR.T |
|  |  |  | 548.8115 | 1095.6083 | 1095.6037 | | | 44 | | | 4.8e-005 | | K.HLIIGVSSDR.G |
|  |  |  | 649.8563 | 1297.6980 | 1297.6918 | | | 64 | | | 1.3e-006 | | R.VYGVGSLALYEK.A |
|  |  |  | 657.8649 | 1313.7152 | 1313.7078 | | | 88 | | | 4.2e-009 | | K.ELIEIISGAAALD.- |
|  |  |  | 584.9731 | 1751.8975 | 1751.8876 | | | 31 | | | 0.0013 | | K.NASEMIDKLTLTFNR.T |
| ATP synthase subunit α | P19483 | 692^a^ | **Observed** | **Mr(expt)** | **Mr(calc)** | | | **Score** | | | **Exp Value** | | **Peptide** |
|  |  |  | 362.2334 | 722.4523 | 722.4439 | | | 34 | | | 0.0022 | | K.APGIIPR.I |
|  |  |  | 1052.5352 | 2103.0558 | 2103.0307 | | | 74 | | | 2e-007 | | K.GMSLNLEPDNVGVVVFGNDK.L |
|  |  |  | 1155.0999 | 2308.1852 | 2308.1521 | | | 73 | | | 2e-007 | | K.QGQYSPMAIEEQVAVIYAGVR.G |
|  |  |  | 1169.5992 | 2337.1838 | 2337.1601 | | | 72 | | | 2.2e-007 | | R.EVAAFAQFGSDLDAATQQLLSR.G |
|  |  |  | 423.2631 | 844.5116 | 844.5018 | | | 37 | | | 0.0028 | | R.STVAQLVK.R |
|  |  |  | 500.7995 | 999.5844 | 999.5713 | | | 32 | | | 0.0088 | | R.VLSIGDGIAR.V |
|  |  |  | 513.8068 | 1025.5990 | 1025.5869 | | | 66 | | | 1.6e-006 | | K.AVDSLVPIGR.G |
|  |  |  | 575.3181 | 1148.6216 | 1148.6077 | | | 35 | | | 0.0047 | | R.GYLDKLEPSK.I |
|  |  |  | 586.3265 | 1170.6384 | 1170.6245 | | | 63 | | | 7.5e-006 | | R.VVDALGNAIDGK.G |
|  |  |  | 644.3587 | 1286.7028 | 1286.6870 | | | 53 | | | 4.7e-005 | | K.HALIIYDDLSK.Q |
|  |  |  | 655.8449 | 1309.6752 | 1309.6554 | | | 62 | | | 7.2e-006 | | K.EIVTNFLAGFEA.- |
|  |  |  | 658.8822 | 1315.7499 | 1315.7347 | | | 64 | | | 2.4e-006 | | K.TSIAIDTIINQK.R |
|  |  |  | 696.3621 | 1390.7096 | 1390.6940 | | | 73 | | | 5.1e-007 | | K.TGTAEVSSILEER.I |
|  |  |  | 776.4347 | 1550.8548 | 1550.8344 | | | 58 | | | 7.7e-006 | | K.LKEIVTNFLAGFEA.- |
|  |  |  | 777.3823 | 1552.7501 | 1552.7310 | | | 65 | | | 3.1e-006 | | R.EAYPGDVFYLHSR.L |
|  |  |  | 788.4059 | 1574.7973 | 1574.7788 | | | 110 | | | 9e-011 | | R.ILGADTSVDLEETGR.V |
|  |  |  | 812.9577 | 1623.9008 | 1623.8832 | | | 33 | | | 0.002 | | R.TGAIVDVPVGEELLGR.V |
|  |  |  | 834.4111 | 1666.8075 | 1666.7872 | | | 76 | | | 2e-007 | | R.NVQAEEMVEFSSGLK.G |
| ATP synthase subunit α | P19483 | 257^b^ | **Observed** | **Mr(expt)** | **Mr(calc)** | | | **Score** | | | **Exp Value** | | **Peptide** |
|  |  |  | 423.2631 | 844.5116 | 844.5018 | | | 28 | | | 0.025 | | R.STVAQLVK.R |
|  |  |  | 500.7986 | 999.5826 | 999.5713 | | | 40 | | | 0.0014 | | R.VLSIGDGIAR.V |
|  |  |  | 513.8066 | 1025.5986 | 1025.5869 | | | 40 | | | 0.00069 | | K.AVDSLVPIGR.G |
|  |  |  | 586.3262 | 1170.6378 | 1170.6245 | | | 39 | | | 0.002 | | R.VVDALGNAIDGK.G |
|  |  |  | 644.3577 | 1286.7009 | 1286.6870 | | | 51 | | | 8.1e-005 | | K.HALIIYDDLSK.Q |
|  |  |  | 655.8438 | 1309.6730 | 1309.6554 | | | 74 | | | 4.7e-007 | | K.EIVTNFLAGFEA.- |
|  |  |  | 658.8825 | 1315.7504 | 1315.7347 | | | 61 | | | 4.7e-006 | | K.TSIAIDTIINQK.R |
|  |  |  | 788.4060 | 1574.7974 | 1574.7788 | | | 104 | | | 4.1e-010 | | R.ILGADTSVDLEETGR.V |
|  |  |  | 780.0700 | 2337.1881 | 2337.1601 | | | 47 | | | 7.4e-005 | | R.EVAAFAQFGSDLDAATQQLLSR.G |
| ATP synthase subunit alpha liver isoform | Q29596 | 663^a^ | **Observed** | **Mr(expt)** | **Mr(calc)** | | | **Score** | | | **Exp Value** | | **Peptide** |
|  |  |  | 500.7950 | 999.5755 | 999.5713 | | | 63 | | | 1.5e-006 | | R.VLSIGDGIAR.V 650 |
|  |  |  | 696.3568 | 1390.6991 | 1390.6940 | | | 68 | | | 3e-007 | | K.TGTAEVSSILEER.I 1903 |
|  |  |  | 788.3997 | 1574.7849 | 1574.7788 | | | 106 | | | 5e-011 | | R.ILGADTSVDLEETGR.V 2427 2428 2431 |
|  |  |  | 834.4048 | 1666.7951 | 1666.7872 | | | 85 | | | 6.3e-009 | | R.NVQAEEMVEFSSGLK.G 2680 2681 2682 2684 |
|  |  |  | 1052.5271 | 2103.0396 | 2103.0307 | | | 63 | | | 6.3e-007 | | K.GMSLNLEPDNVGVVVFGNDK.L |
| ATP synthase subunit alpha liver isoform | Q29596 | 317^b^ | **Observed** | **Mr(expt)** | **Mr(calc)** | | | **Score** | | | **Exp Value** | | **Peptide** |
|  |  |  | 500.7958 | 999.5769 | 999.5713 | | | 35 | | | 0.001 | | R.VLSIGDGIAR.V 665 |
|  |  |  | 696.3574 | 1390.7002 | 1390.6940 | | | 74 | | | 9.9e-008 | | K.TGTAEVSSILEER.I 1888 |
|  |  |  | 788.4000 | 1574.7855 | 1574.7788 | | | 107 | | | 4.4e-011 | | R.ILGADTSVDLEETGR.V |
|  |  |  | 834.4047 | 1666.7949 | 1666.7872 | | | 96 | | | 4.7e-010 | | R.NVQAEEMVEFSSGLK.G |
|  |  |  | 1060.5248 | 2119.0350 | 2119.0256 | | | 79 | | | 1.9e-008 | | K.GMSLNLEPDNVGVVVFGNDK.L |
| Mitochondrial import receptor subunit TOM40 homolog | Q1LZB5 | 109^a^ | **Observed** | **Mr(expt)** | **Mr(calc)** | | | **Score** | | | **Exp Value** | | **Peptide** |
|  |  |  | 353.2197 | 704.4249 | 704.4221 | | | 45 | | | 9.6e-005 | | K.ANLLFK.G |
|  |  |  | 706.8362 | 1411.6578 | 1411.6521 | | | 46 | | | 5.7e-005 | | K.FVNWQVDGEYR.G |
|  |  |  | 788.4090 | 1574.8034 | 1574.7940 | | | 66 | | | 7.4e-007 | | K.GSVDSNWIVGATLEK.K |
| Mitochondrial import receptor subunit TOM40 homolog | Q1LZB5 | 104^b^ | **Observed** | **Mr(expt)** | **Mr(calc)** | | | **Score** | | | **Exp Value** | | **Peptide** |
|  |  |  | 353.2196 | 704.4246 | 704.4221 | | | 37 | | | 0.007 | | K.ANLLFK.G |
|  |  |  | 706.8361 | 1411.6575 | 1411.6521 | | | 53 | | | 0.00012 | | K.FVNWQVDGEYR.G |
|  |  |  | 788.4083 | 1574.8020 | 1574.7940 | | | 73 | | | 1.6e-006 | | K.GSVDSNWIVGATLEK.K |
| Mitochondrial fission 1 protein | Q3T0I5 | 129^a^ | **Observed** | **Mr(expt)** | **Mr(calc)** | | | **Score** | | | **Exp Value** | | **Peptide** |
|  |  |  | 720.8835 | 1439.7523 | 1439.7368 | | | 65 | | | 3.4e-007 | | R.GLLQTEPQNNQAK.E |
|  |  |  | 598.3715 | 1194.7285 | 1194.7223 | | | 84 | | | 6.6e-009 | | K.GLALLEELLPK.G |
| Mitochondrial fission 1 protein | Q3T0I5 | 134^b^ | **Observed** | **Mr(expt)** | **Mr(calc)** | | | **Score** | | | **Exp Value** | | **Peptide** |
|  |  |  | 720.8836 | 1439.7525 | 1439.7368 | | | 74 | | | 5.4e-008 | | R.GLLQTEPQNNQAK.E |
|  |  |  | 598.3713 | 1194.7280 | 1194.7223 | | | 48 | | | 7.3e-005 | | K.GLALLEELLPK.G |
| Alpha-1-antiproteinase | P34955 | 142^a^ | **Observed** | **Mr(expt)** | **Mr(calc)** | | | **Score** | | | **Exp Value** | | **Peptide** |
|  |  |  | 589.8263 | 1177.6380 | 1177.6231 | | | 60 | | | 1.1e-005 | | K.LVDTFLEDVK.N |
|  |  |  | 659.8252 | 1317.6358 | 1317.6201 | | | 42 | | | 0.00067 | | R.DFHVDEQTTVK.V |
|  |  |  | 797.4200 | 1592.8255 | 1592.8046 | | | 79 | | | 1.2e-007 | | K.SVLGDVGITEVFSDR.A |
|  |  |  | 647.3634 | 1939.0685 | 1939.0455 | | | 42 | | | 0.00025 | | K.VLDPNTVFALVNYISFK.G |
| Alpha-1-antiproteinase | P34955 | 180^b^ | **Observed** | **Mr(expt)** | **Mr(calc)** | | | **Score** | | | **Exp Value** | | **Peptide** |
|  |  |  | 584.8108 | 1167.6070 | 1167.6023 | | | 59 | | | 4.4e-005 | | K.LSISETYDLK.S |
|  |  |  | 589.8212 | 1177.6278 | 1177.6231 | | | 63 | | | 2.3e-005 | | K.LVDTFLEDVK.N |
|  |  |  | 659.8193 | 1317.6240 | 1317.6201 | | | 47 | | | 0.00057 | | R.DFHVDEQTTVK.V |
|  |  |  | 797.4130 | 1592.8114 | 1592.8046 | | | 89 | | | 3.8e-008 | | K.SVLGDVGITEVFSDR.A |
|  |  |  | 647.3583 | 1939.0530 | 1939.0455 | | | 50 | | | 9.7e-005 | | K.VLDPNTVFALVNYISFK.G |
| AnnexinA2 | P04272 | 163^a^ | **Observed** | **Mr(expt)** | **Mr(calc)** | | | **Score** | | | **Exp Value** | | **Peptide** |
|  |  |  | 518.2745 | 1034.5343 | 1034.5219 | | | 29 | | | 0.019 | | K.WISIMTER.S |
|  |  |  | 543.7540 | 1085.4935 | 1085.4778 | | | 37 | | | 0.0014 | | K.AYTNFDAER.D |
|  |  |  | 556.2870 | 1110.5595 | 1110.5458 | | | 71 | | | 9.4e-007 | | R.QDIAFAYQR.R |
|  |  |  | 611.8080 | 1221.6015 | 1221.5877 | | | 46 | | | 0.00034 | | K.TPAQYDASELK.A |
|  |  |  | 622.8220 | 1243.6295 | 1243.6156 | | | 49 | | | 0.00016 | | R.TNQELQEINR.V |
|  |  |  | 711.3581 | 1420.7017 | 1420.6874 | | | 62 | | | 6.1e-006 | | K.SLYYYIQQDTK.G |
|  |  |  | 551.0050 | 1649.9931 | 1649.9716 | | | 34 | | | 0.00041 | | K.SALSGHLETVILGLLK.T |
| AnnexinA2 | P04272 | 224^b^ | **Observed** | **Mr(expt)** | **Mr(calc)** | | | **Score** | | | **Exp Value** | | **Peptide** |
|  |  |  | 556.2824 | 1110.5502 | 1110.5458 | | | 47 | | | 0.00087 | | R.QDIAFAYQR.R |
|  |  |  | 611.8030 | 1221.5915 | 1221.5877 | | | 56 | | | 0.0001 | | K.TPAQYDASELK.A |
|  |  |  | 622.8170 | 1243.6194 | 1243.6156 | | | 51 | | | 0.00033 | | R.TNQELQEINR.V |
|  |  |  | 711.3542 | 1420.6938 | 1420.6874 | | | 66 | | | 8.1e-006 | | K.SLYYYIQQDTK.G |
|  |  |  | 771.9316 | 1541.8485 | 1541.8413 | | | 98 | | | 3.8e-009 | | K.GVDEVTIVNILTNR.S |
|  |  |  | 551.0003 | 1649.9789 | 1649.9716 | | | 55 | | | 5.8e-006 | | K.SALSGHLETVILGLLK.T |
|  |  |  | 719.0305 | 2154.0696 | 2154.0593 | | | 36 | | | 0.0048 | | K.AYTNFDAERDALNIETAIK.T |
| Histone H4 | P62802 | 179^a^ | **Observed** | **Mr(expt)** | **Mr(calc)** | | | **Score** | | | **Exp Value** | | **Peptide** |
|  |  |  | 495.2946 | 988.5747 | 988.5706 | | | 44 | | | 6.9e-005 | | K.VFLENVIR.D |
|  |  |  | 590.8160 | 1179.6175 | 1179.6135 | | | 41 | | | 0.00018 | | R.ISGLIYEETR.G |
|  |  |  | 655.8580 | 1309.7015 | 1309.6952 | | | 67 | | | 5.2e-007 | | K.TVTAMDVVYALK.R |
|  |  |  | 663.8546 | 1325.6947 | 1325.6901 | | | 64 | | | 8.9e-007 | | K.TVTAMDVVYALK.R |
|  |  |  | 480.2727 | 1437.7962 | 1437.7901 | | | 44 | | | 9e-005 | | R.KTVTAMDVVYALK.R |
|  |  |  | 489.6082 | 1465.8026 | 1465.7963 | | | 30 | | | 0.0014 | | K.TVTAMDVVYALKR.Q |
| Histone H4 | P62802 | 133^b^ | **Observed** | **Mr(expt)** | **Mr(calc)** | | | **Score** | | | **Exp Value** | | **Peptide** |
|  |  |  | 495.2985 | 988.5825 | 988.5706 | | | 51 | | | 7e-005 | | K.VFLENVIR.D |
|  |  |  | 590.8210 | 1179.6273 | 1179.6135 | | | 44 | | | 0.00044 | | R.ISGLIYEETR.G |
|  |  |  | 663.8607 | 1325.7069 | 1325.6901 | | | 72 | | | 6.2e-007 | | K.TVTAMDVVYALK.R |

^a^ Protein score for proteins detected in ECVs.

^b^ Protein score for proteins detected in latex bead phagosomes.

^c^ 14-3-3 ζ/δ (Q5R651) was detected in two of four latex bead phagolysosomes.

^d^ 14-3-3θ (Q0VC36) was detected in two of four *E. chaffeensis* phagosomes.

^e^ 14-3-3γ(P68253) was detected in two of four latex bead phagolysosomes, and was also detected in two of four *E. chaffeensis* phagosomes.
