# Supplementary material for: Proteomic Analysis of the Ehrlichia chaffeensis Phagosome in Cultured DH82 Cells
Source: PLoS One. 2014 Feb 18;9(2):e88461. doi: 10.1371/journal.pone.0088461 (PMC3928192; doi:10.1371/journal.pone.0088461)
Supplement: Table S5 — Mascot results for proteins detected in all of latex bead phagosomes that were not detectable in any ECVs. (DOCX) [file pone.0088461.s005.docx]

**Supplemental Table S5: Mascot results for proteins detected in all of latex bead phagosomes that were not detectable in any ECVs**

| **Protein identified** | **Accession No.** | **Protein Score** | **Mascot results** | | | | | |
| --- | --- | --- | --- | --- | --- | --- | --- | --- |
| Lactadherin | P79385 | 88 | **Observed** | **Mr(expt)** | **Mr(calc)** | **Score** | **Exp Value** | **Peptide** |
|  |  |  | 728.3455 | 1455.6836 | 1455.6830 | 57 | 5.7e-06 | K.FQFIQGAEESGDK.I |
|  |  |  | 792.3933 | 1583.7793 | 1583.7789 | 34 | 0.0017 | R.KFQFIQGAEESGDK.I |
|  |  |  | 471.2410 | 1411.7085 | 1411.7081 | 26 | 0.0045 | R.DFGHIQYVAAYK.V |
| Flotillin-1 | Q5RBL4 | 397 | **Observed** | **Mr(expt)** | **Mr(calc)** | **Score** | **Exp Value** | **Peptide** |
|  |  |  | 478.7819 | 955.5492 | 955.5451 | 41 | 0.0022 | R.VQVQVVER.A |
|  |  |  | 485.7620 | 969.5095 | 969.5066 | 39 | 0.0049 | R.SPPVMVAGGR.V |
|  |  |  | 576.2902 | 1150.5658 | 1150.5618 | 58 | 7.2e-005 | K.AAYDIEVNTR.R |
|  |  |  | 608.3710 | 1214.7275 | 1150.5618 | 84 | 2.8e-008 | R.ISLNTLTLNVK.S |
|  |  |  | 469.2790 | 1404.8151 | 1404.8089 | 40 | 0.00074 | R.HGVPISVTGIAQVK.I |
|  |  |  | 734.8733 | 1467.7320 | 1467.7279 | 54 | 0.00012 | K.VSAQYLSEIEMAK.A |
|  |  |  | 735.3913 | 1468.7681 | 1468.7634 | 88 | 4e-008 | R.AQQVAVQEQEIAR.R |
|  |  |  | 552.5958 | 1654.7656 | 1654.7587 | 35 | 0.0049 | K.DIHDDQDYLHSLGK.A |
|  |  |  | 831.4149 | 1660.8153 | 1660.8090 | 91 | 2.5e-008 | K.SQLIMQAEAEAESVR.M |
|  |  |  | 877.4730 | 1752.9315 | 1752.9258 | 83 | 8.6e-008 | K.LPQVAEEISGPLTSANK.I |
|  |  |  | 667.0217 | 1998.0433 | 1998.0343 | 47 | 0.00028 | K.VASSDLVNMGISVVSYTLK.D |
|  |  |  | 673.3525 | 2017.0357 | 2017.0228 | 53 | 8.6e-005 | K.TEAEIAHIALETLEGHQR.A |
|  |  |  | 785.7387 | 2354.1942 | 2354.1827 | 47 | 0.00024 | K.KAEAFQLYQEAAQLDMLLEK.L |
| Myoferlin | Q9NZM1 | 236 | **Observed** | **Mr(expt)** | **Mr(calc)** | **Score** | **Exp Value** | **Peptide** |
|  |  |  | 462.2470 | 922.4795 | 922.4760 | 23 | 0.27 | K.TASLFEQK.S |
|  |  |  | 466.7529 | 931.4913 | 931.4876 | 46 | 0.0017 | K.WGTSGLVGR.H |
|  |  |  | 575.7914 | 1149.5683 | 1149.5626 | 37 | 0.0088 | K.DLTQTASSTAR.A |
|  |  |  | 578.8348 | 1155.6550 | 1155.6499 | 65 | 1e-005 | R.VIVESASNIPK.T |
|  |  |  | 601.8196 | 1201.6247 | 1201.6190 | 61 | 3.8e-005 | K.LIDEVIEDTR.Y |
|  |  |  | 629.8374 | 1257.6602 | 1257.6565 | 80 | 3.5e-007 | K.VGETIIDLENR.F |
|  |  |  | 631.8373 | 1261.6599 | 1261.6529 | 30 | 0.034 | K.LQMWVDVFPK.S |
|  |  |  | 690.3301 | 1378.6457 | 1378.6405 | 48 | 0.00038 | K.ISVYDYDTFTR.D |
|  |  |  | 761.4047 | 1520.7948 | 1520.7875 | 57 | 5.9e-005 | K.NLVDPFVEVSFAGK.K |
|  |  |  | 781.7402 | 2342.1987 | 2342.1906 | 53 | 5.3e-005 | K.KVDNELNPVWNEILEFDLR.G |
|  |  |  | 871.3997 | 2611.1772 | 2611.1674 | 51 | 4.4e-005 | R.SLLTEADAGHTEFTDEVYQNESR.Y |
| Erythrocyte band 7 integral membrane protein | P27105 | 304 | **Observed** | **Mr(expt)** | **Mr(calc)** | **Score** | **Exp Value** | **Peptide** |
|  |  |  | 427.2678 | 852.5211 | 852.5181 | 34 | 0.0027 | K.LPVQLQR.A |
|  |  |  | 523.2875 | 1044.5605 | 1044.5564 | 56 | 0.0002 | K.NLSQILSDR.E |
|  |  |  | 676.3802 | 1350.7458 | 1350.7395 | 65 | 6e-006 | R.YLQTLTTIAAEK.N |
|  |  |  | 801.4464 | 1600.8783 | 1600.8712 | 43 | 0.00075 | R.TISFDIPPQEILTK.D |
|  |  |  | 858.4565 | 1714.8984 | 1714.8923 | 93 | 1e-008 | K.EASMVITESPAALQLR.Y |
|  |  |  | 866.4536 | 1730.8927 | 1730.8873 | 81 | 1.7e-007 | K.EASMVITESPAALQLR.Y |
|  |  |  | 965.5071 | 1928.9997 | 1928.9915 | 122 | 1.2e-011 | R.VQNATLAVANITNADSATR.L |
| Ras-related protein Rab-35 | Q5U316 | 157 | **Observed** | **Mr(expt)** | **Mr(calc)** | **Score** | **Exp Value** | **Peptide** |
|  |  |  | 502.7577 | 1003.5009 | 1003.4975 | 23 | 0.33 | R.TITSTYYR.G |
|  |  |  | 527.2604 | 1052.5063 | 1052.5026 | 34 | 0.016 | K.VVETEDAYK.F |
|  |  |  | 536.3264 | 1070.6382 | 1070.6336 | 57 | 4.3e-005 | K.LLIIGDSGVGK.S |
|  |  |  | 658.8359 | 1315.6572 | 1315.6521 | 52 | 0.00023 | K.LQIWDTAGQER.F |
|  |  |  | 822.4116 | 1642.8087 | 1642.8025 | 91 | 2.1e-008 | K.FAGQMGIQLFETSAK.E |
| DLA class Ⅰhistocompatibility antigen, A9/A9 alpha chain | P18466 | 155 | **Observed** | **Mr(expt)** | **Mr(calc)** | **Score** | **Exp Value** | **Peptide** |
|  |  |  | 416.2338 | 830.4530 | 830.4498 | 29 | 0.097 | R.VDLDTLR.G |
|  |  |  | 713.8781 | 1425.7416 | 1425.7365 | 55 | 8.2e-005 | K.WAAVVVPSGQEQR.Y |
|  |  |  | 759.8582 | 1517.7018 | 1517.6958 | 96 | 4.8e-009 | R.DDSAQGSDVSLTAPR.V |
|  |  |  | 870.3945 | 2608.1617 | 2608.1525 | 65 | 1.3e-006 | R.DGEDQTQDTEVVDTRPAGDGTFQK.W |
| Ras-related protein Rab31 | Q6GQP4 | 146 | **Observed** | **Mr(expt)** | **Mr(calc)** | **Score** | **Exp Value** | **Peptide** |
|  |  |  | 654.3667 | 1306.7189 | 1306.7133 | 74 | 8.3e-007 | R.GSAAAVIVYDITK.Q |
|  |  |  | 668.3415 | 1334.6684 | 1334.6619 | 68 | 6.5e-006 | K.FLIWDTAGQER.F |
|  |  |  | 802.4285 | 1602.8425 | 1602.8365 | 71 | 2.3e-006 | K.NAINIEELFQGISR.Q |
| Ras-related C3 botulinum toxin substrate 1 | Q6RUV5 | 145 | **Observed** | **Mr(expt)** | **Mr(calc)** | **Score** | **Exp Value** | **Peptide** |
|  |  |  | 752.4181 | 1502.8216 | 1502.8167 | 48 | 0.00034 | K.LTPITYPQGLAMAK.E |
|  |  |  | 760.4156 | 1518.8166 | 1518.8116 | 31 | 0.016 | K.LTPITYPQGLAMAK.E |
|  |  |  | 549.9784 | 1646.9133 | 1646.9065 | 45 | 0.00029 | K.KLTPITYPQGLAMAK.E |
|  |  |  | 974.9604 | 1947.9062 | 1947.8963 | 97 | 2.8e-009 | K.PVNLGLWDTAGQEDYDR.L |
| ATP-binding cassette sub-family B member 6 | Q9DC29 | 135 | **Observed** | **Mr(expt)** | **Mr(calc)** | **Score** | **Exp Value** | **Peptide** |
|  |  |  | 346.8667 | 1037.5782 | 1037.5730 | 32 | 0.0015 | R.GRHEALLSR.G |
|  |  |  | 1072.5433 | 2143.0720 | 2143.0644 | 133 | 6.4e-013 | K.APDIILLDEATSALDTSNER.A |
| Plasma membrane calcium-transporting ATPase 1 | P11505 | 133 | **Observed** | **Mr(expt)** | **Mr(calc)** | **Score** | **Exp Value** | **Peptide** |
|  |  |  | 604.2931 | 1206.5717 | 1206.5663 | 59 | 4.2e-005 | R.MVTGDNINTAR.A |
|  |  |  | 659.8392 | 1317.6639 | 1317.6598 | 31 | 0.03 | K.TVIEPMASEGLR.T |
|  |  |  | 769.8823 | 1537.7501 | 1537.7447 | 63 | 1.4e-005 | K.ADVGFAMGIAGTDVAK.E |
|  |  |  | 940.9814 | 1879.9482 | 1879.9415 | 73 | 1.3e-006 | K.EASDIILTDDNFTSIVK.A |
| Ras-related protein R-Ras | D3Z8L7 | 131 | **Observed** | **Mr(expt)** | **Mr(calc)** | **Score** | **Exp Value** | **Peptide** |
|  |  |  | 445.7781 | 889.5416 | 889.5385 | 21 | 0.036 | K.LFTQILR.V |
|  |  |  | 716.8787 | 1431.7429 | 1431.7358 | 97 | 2.5e-008 | R.LNVDEAFEQLVR.T |
|  |  |  | 891.4257 | 1780.8369 | 1780.8302 | 73 | 1e-006 | R.LDILDTAGQEEFGAMR.E |
| Rho-related GTP-binding protein RhoG | P84095 | 125 | **Observed** | **Mr(expt)** | **Mr(calc)** | **Score** | **Exp Value** | **Peptide** |
|  |  |  | 1012.4758 | 2022.9370 | 2022.9283 | 78 | 2.2e-007 | R.TVNLNLWDTAGQEEYDR.L |
|  |  |  | 1066.5054 | 2130.9962 | 2130.9858 | 75 | 4.2e-007 | K.EYIPTVFDNYSAQSAVDGR.T |
| AP-2 complex subunit alpha-2 | Q0VCK5 | 117 | **Observed** | **Mr(expt)** | **Mr(calc)** | **Score** | **Exp Value** | **Peptide** |
|  |  |  | 399.7593 | 797.5041 | 797.5011 | 26 | 0.053 | K.LPITLNK.F |
|  |  |  | 511.3205 | 1020.6264 | 1020.6219 | 44 | 0.00023 | R.ALLLSTYIK.F |
|  |  |  | 545.8187 | 1089.6229 | 1089.6182 | 61 | 1.9e-005 | R.GLAVFISDIR.N |
|  |  |  | 632.8283 | 1263.6421 | 1263.6381 | 41 | 0.0031 | K.ILVAGDTMDSVK.Q |
|  |  |  | 924.4906 | 1846.9667 | 1846.9577 | 67 | 3.9e-006 | K.VGGYILGEFGNLIAGDPR.S |
| Receptor-type tyrosine-protein phosphatase C | P06800 | 116 | **Observed** | **Mr(expt)** | **Mr(calc)** | **Score** | **Exp Value** | **Peptide** |
|  |  |  | 591.7591 | 1181.5037 | 1181.4989 | 45 | 0.00036 | R.DETVDDFWR.M |
|  |  |  | 660.8720 | 1319.7295 | 1319.7238 | 70 | 2.2e-006 | R.LFLAEFQSIPR.V |
|  |  |  | 715.8539 | 1429.6932 | 1429.6878 | 28 | 0.048 | R.YVDILPYDYNR.V |
|  |  |  | 843.9035 | 1685.7923 | 1685.7872 | 63 | 1.2e-005 | K.ETIGDFWQMIFQR.K |
| Lipid phosphate phosphohydrolase 1 | O14494 | 153 | **Observed** | **Mr(expt)** | **Mr(calc)** | **Score** | **Exp Value** | **Peptide** |
|  |  |  | 991.5377 | 1981.0609 | 1981.0520 | 115 | 3.9e-011 | K.AIGTFLFGAAASQSLTDIAK.Y |
|  |  |  | 920.0749 | 1842.1678 | 1842.1646 | 61 | 2.7e-005 | K.GDWARLLRPTLQFGLV.A |
| Vacuolar fusion protein MON1 homolog B | Q4R4E4 | 104 | **Observed** | **Mr(expt）** | **Mr(calc)** | **Score** | **Exp Value** | **Peptide** |
|  |  |  | 414.7523 | 827.4901 | 827.4865 | 43 | 0.0022 | R.DALGALLR.R |
|  |  |  | 574.3234 | 1146.6322 | 1146.6285 | 64 | 1.5e-005 | K.ETLLAWVTSK.F |
|  |  |  | 783.8747 | 1565.7348 | 1565.7303 | 66 | 5.4e-006 | R.FNPDGFFYAYVAR.L |
| Vesicle-associated membrane protein 3 | Q2KJD2 | 96 | **Observed** | **Mr(expt）** | **Mr(calc)** | **Score** | **Exp Value** | **Peptide** |
|  |  |  | 833.4103 | 1664.8060 | 1664.8006 | 70 | 3.1e-006 | R.ADALQAGASQFETSAAK.L |
|  |  |  | 832.0766 | 2493.2080 | 2493.1983 | 58 | 2.1e-005 | K.LSELDDRADALQAGASQFETSAAK.L |
| Secretory carrier-associated membrane protein 3 | Q58DR5 | 125 | **Observed** | **Mr(expt)** | **Mr(calc)** | **Score** | **Exp Value** | **Peptide** |
|  |  |  | 738.8650 | 1475.7155 | 1475.7117 | 94 | 1.2e-008 | R.TAAANAAAGAAENAFR.A |
|  |  |  | 747.0234 | 1492.5086 | 1492.5046 | 48 | 0.00053 | K.NYGSYSTQASTAAAT.A |
| Solute carrier family 12 member 9 | Q66HR0 | 93 | **Observed** | **Mr(expt)** | **Mr(calc)** | **Score** | **Exp Value** | **Peptide** |
|  |  |  | 652.8664 | 1303.7182 | 1303.7136 | 48 | 0.0004 | K.AFVDLTLSPSVR.Q |
|  |  |  | 831.4443 | 1660.8740 | 1660.8672 | 76 | 5.2e-007 | R.ALSPQDYVATVADALK.M |
| Ras-related protein Rab-13 | Q5KTJ6 | 90 | **Observed** | **Mr(expt)** | **Mr(calc)** | **Score** | **Exp Value** | **Peptide** |
|  |  |  | 415.2099 | 828.4052 | 828.4018 | 28 | 0.066 | R.FFETSAK.S |
|  |  |  | 536.3264 | 1070.6382 | 1070.6336 | 57 | 4.3e-005 | K.LLLIGDSGVGK.T |
|  |  |  | 827.4276 | 1652.8406 | 1652.8331 | 55 | 7.8e-005 | R.GAMGIILVYDITDEK.S |
| Platelet glycoprotein 4 | Q07969 | 112 | **Observed** | **Mr(expt)** | **Mr(calc）** | **Score** | **Exp Value** | **Peptide** |
|  |  |  | 667.8665 | 1333.7185 | 1333.7129 | 84 | 1.1e-007 | R.EVVLEEGTIAFK.N |
|  |  |  | 692.9978 | 1385.6157 | 1385.6139 | 45 | 0.00055 | K.TIKREVVLEEGTI.A |
|  |  |  | 822.5087 | 1643.0059 | 1643.0023 | 44 | 0.00033 | R.NQVTGKIKLLGLVEM.V |
| Stabilin-1 | Q9NY15 | 170 | **Observed** | **Mr(expt)** | **Mr(calc)** | **Score** | **Exp Value** | **Peptide** |
|  |  |  | 902.4418 | 1802.8691 | 1802.8615 | 81 | 1.5e-007 | R.GLDFLDFLDDELTYK.T |
|  |  |  | 875.5735 | 1748.8749 | 1748.8706 | 51 | 0.00019 | K.GQAQCHCPENYHGDGM.V |
|  |  |  | 849.5649 | 1700.9101 | 1700.9067 | 52 | 0.00019 | R.VWVQNASVDVADLLAT.N |
|  |  |  | 937.7968 | 1877.2204 | 1877.2189 | 45 | 0.00071 | K.VAGLLPLLREASHRPFT.M |
|  |  |  | 578.0026 | 1154.3443 | 1154.3417 | 73 | 1.3e-006 | R.ARGKPMGFGFS.A |
| Transforming protein RhoA | Q5REY6 | 87 | **Observed** | **Mr(expt)** | **Mr(calc)** | **Score** | **Exp Value** | **Peptide** |
|  |  |  | 499.7358 | 997.4571 | 997.4539 | 25 | 0.086 | R.EVFEMATR.A |
|  |  |  | 1004.9703 | 2007.9260 | 2007.9174 | 79 | 1.8e-007 | K.QVELALWDTAGQEDYDR.L |
|  |  |  | 925.1163 | 2772.3270 | 2772.3171 | 35 | 0.003 | K.DQFPEVYVPTVFENYVADIEVDGK.Q |
| Intercellular adhesion molecule 1 | P33729 | 72 | **Observed** | **Mr(expt)** | **Mr(calc)** | **Score** | **Exp Value** | **Peptide** |
|  |  |  | 447.7757 | 893.5369 | 893.5334 | 36 | 0.0045 | R.VELAPLPR.W |
|  |  |  | 516.2921 | 1030.5696 | 1030.5659 | 41 | 0.0033 | K.DSLLATANVK.A |
|  |  |  | 763.4243 | 1524.8339 | 1524.8300 | 59 | 2.4e-005 | R.QLQTFVLPETPPR.L |
| Synaptotagmin-like protein 4 | Q8VHQ7 | 87 | **Observed** | **Mr(expt)** | **Mr(calc）** | **Score** | **Exp Value** | **Peptide** |
|  |  |  | 528.8263 | 1055.6381 | 1055.6339 | 46 | 0.00043 | R.DLILSVLQR.D |
|  |  |  | 690.5291 | 1380.5079 | 1380.5038 | 75 | 1.3e-007 | K.ISTESSPGLPAHKG.E |
| DnaJ homolog subfamily C member 5 | Q9H3Z4 | 76 | **Observed** | **Mr(expt)** | **Mr(calc)** | **Score** | **Exp Value** | **Peptide** |
|  |  |  | 556.3024 | 1665.8853 | 1665.8798 | 54 | 0.00012 | K.EINNAHAILTDATKR.N |
|  |  |  | 971.2259 | 1940.9841 | 1940.9802 | 48 | 0.0004 | K.APEGEETEFYVSPEDLE.A |
| Lysosome-associated membrane glycoprotein 2 | P13473 | 101 | **Observed** | **Mr(expt)** | **Mr(calc)** | **Score** | **Exp Value** | **Peptide** |
|  |  |  | 494.2864 | 986.5582 | 986.5549 | 41 | 0.0052 | R.IPLNDLFR.C |
|  |  |  | 551.3050 | 1100.5954 | 1100.5906 | 47 | 0.00098 | K.YLDFVFAVK.N |
|  |  |  | 1006.5134 | 2011.0122 | 2011.0011 | 69 | 2.4e-006 | K.EQTVSVSGAFQINTFDLR.V |
| Lysosome-associated membrane glycoprotein 1 | P11279 | 110 | **Observed** | **Mr(expt)** | **Mr(calc)** | **Score** | **Exp Value** | **Peptide** |
|  |  |  | 439.02 | 875.01 | 874.81 | 62 | 5.5e-006 | R.SHAGYQTI |
|  |  |  | 464.21 | 925.23 | 924.78 | 43 | 0.00093 | K.AFSVNIFK.V |
|  |  |  | 576.02 | 1152.05 | 1152.5 | 46 | 0.00037 | R.ALQATVGNSYK.C |
|  |  |  | 516.89 | 1031.89 | 1031.51 | 46 | 0.00029 | K.TVESITDIR.A |
| Acid ceramidase | A5A6P2 | 157 | **Observed** | **Mr(expt)** | **Mr(calc)** | **Score** | **Exp Value** | **Peptide** |
|  |  |  | 605.3108 | 1208.6070 | 1208.6012 | 66 | 8.9e-006 | K.DAMWIGFLTR.T |
|  |  |  | 613.3080 | 1224.6014 | 1224.5961 | 46 | 0.00099 | K.DAMWIGFLTR.T |
|  |  |  | 937.9745 | 1873.9344 | 1873.9284 | 74 | 8.2e-007 | K.LPGLLGNFPGPFEEEMK.G |
|  |  |  | 945.9735 | 1889.9325 | 1889.9233 | 59 | 2.6e-005 | K.LPGLLGNFPGPFEEEMK.G |
| Tripeptidyl-peptidase 1 | Q9XSB8 | 153 | **Observed** | **Mr(expt)** | **Mr(calc)** | **Score** | **Exp Value** | **Peptide** |
|  |  |  | 434.7169 | 867.4193 | 867.4160 | 33 | 0.02 | R.VNTEFMK.A |
|  |  |  | 468.2531 | 934.4916 | 934.4872 | 49 | 0.00078 | R.GAGLFDVTR.G |
|  |  |  | 747.4492 | 1492.8838 | 1492.8766 | 60 | 3.3e-006 | R.LLSGLPPLGFLNPR.L |
|  |  |  | 1222.6020 | 2443.1894 | 2443.1809 | 79 | 1.4e-007 | R.PSFPASSPYVTTVGGTSFQNPFR.V |
| Cathepsin D | Q4LAL9 | 288 | **Observed** | **Mr(expt)** | **Mr(calc)** | **Score** | **Exp Value** | **Peptide** |
|  |  |  | 523.3073 | 1044.6001 | 1044.5968 | 29 | 0.05 | K.QPGITFIAAK.F |
|  |  |  | 528.2681 | 1054.5216 | 1054.5182 | 23 | 0.22 | K.LSSEDYTLK.V |
|  |  |  | 536.8180 | 1071.6213 | 1071.6176 | 30 | 0.028 | K.VSTLPDVTLK.L |
|  |  |  | 587.3087 | 1172.6028 | 1172.5978 | 44 | 0.002 | K.NIFSFYLNR.D |
|  |  |  | 620.3162 | 1238.6179 | 1238.6118 | 65 | 1.1e-005 | K.FDGILGMAYPR.I |
|  |  |  | 628.3130 | 1254.6115 | 1254.6067 | 43 | 0.0016 | K.FDGILGMAYPR.I |
|  |  |  | 893.9209 | 1785.8273 | 1785.8203 | 89 | 1.7e-008 | R.DPNAQPGGELMLGGTDSK.Y |
|  |  |  | 901.9183 | 1801.8220 | 1801.8152 | 95 | 3.8e-009 | R.DPNAQPGGELMLGGTDSK.Y |
|  |  |  | 653.6871 | 1958.0396 | 1958.0295 | 42 | 0.00081 | R.ISVNNVLPVFDNLMQQK.L |
|  |  |  | 659.0180 | 1974.0323 | 1974.0244 | 35 | 0.005 | R.ISVNNVLPVFDNLMQQK.L |
|  |  |  | 981.1462 | 2940.4167 | 2940.4076 | 47 | 0.00014 | K.NIFSFYLNRDPNAQPGGELMLGGTDSK.Y |
|  |  |  | 986.4791 | 2956.4154 | 2956.4025 | 42 | 0.00042 | K.NIFSFYLNRDPNAQPGGELMLGGTDSK.Y |
| Cathepsin S | Q8HY81 | 95 | **Observed** | **Mr(expt)** | **Mr(calc)** | **Score** | **Exp Value** | **Peptide** |
|  |  |  | 412.2227 | 822.4309 | 822.4276 | 25 | 0.16 | K.DYWLVK.N |
|  |  |  | 494.2506 | 986.4866 | 986.4821 | 49 | 0.00048 | K.LPDSVDWR.E |
|  |  |  | 735.3645 | 1468.7144 | 1468.7086 | 53 | 0.00014 | K.YTELPFGSEDALK.E |
|  |  |  | 813.8901 | 1625.7657 | 1625.7587 | 49 | 0.00026 | K.NSWGLNFGDQGYIR.M |
|  |  |  | 677.0139 | 2028.0200 | 2028.0105 | 29 | 0.024 | K.GPVSVAIDASHYSFFLYR.S |
| Cathepsin Z | Q9UBR2 | 169 | **Observed** | **Mr(expt)** | **Mr(calc)** | **Score** | **Exp Value** | **Peptide** |
|  |  |  | 654.8425 | 1307.6758 | 1307.6719 | 98 | 2.8e-009 | R.NVDGVNYASITR.N |
|  |  |  | 505.7429 | 1009.4913 | 1009.4896 | 39 | 0.0027 | R.VGDYGSLSGR.E |
|  |  |  | 371.2168 | 1110.6202 | 1110.6181 | 87 | 4.5e-008 | R.IVTSTYKDGK.G |
| Alpha-N-acetylglucosaminidase | P54802 | 85 | **Observed** | **Mr(expt)** | **Mr(calc)** | **Score** | **Exp Value** | **Peptide** |
|  |  |  | 505.2422 | 1008.4699 | 1008.4665 | 49 | 0.00049 | R.SDVFEAWR.L |
|  |  |  | 1048.0328 | 2094.0510 | 2094.0422 | 68 | 3.1e-006 | R.YQLTLWGPEGNILDYANK.Q |
| Lysosomal acid phosphatase | Q0P5F0 | 88 | **Observed** | **Mr(expt)** | **Mr(calc)** | **Score** | **Exp Value** | **Peptide** |
|  |  |  | 584.3660 | 1166.7174 | 1166.7135 | 72 | 3.1e-007 | R.LQGGVLLAQIR.K |
|  |  |  | 822.9671 | 1645.8931 | 1645.8900 | 45 | 0.0014 | K.FPLGPCPRFEQLQN.E |
| Cathepsin-K | Q3ZKN1 | 66 | **Observed** | **Mr(expt)** | **Mr(calc)** | **Score** | **Exp Value** | **Peptide** |
|  |  |  | 412.2300 | 822.4455 | 822.4422 | 39 | 0.0049 | K.GYILMAR.N |
|  |  |  | 461.7184 | 921.4223 | 921.4192 | 52 | 0.00025 | R.APDSVDYR.K |
|  |  |  | 596.2650 | 1190.5155 | 1190.5105 | 40 | 0.0011 | K.NSWGENWGNK.G |
| N-acetylglucosamine-6-salfatase | Q1LZH9 | 74 | **Observed** | **Mr(expt)** | **Mr(calc)** | **Score** | **Exp Value** | **Peptide** |
|  |  |  | 528.2761 | 1054.5375 | 1054.5335 | 37 | 0.00069 | R.QLYEFDIK.V |
|  |  |  | 676.3303 | 1350.6460 | 1350.6416 | 57 | 6.3e-005 | R.SDVLVEYQGEGR.N |
| Vacuolar fusion protein CCZ1 homolog | Q0VD30 | 109 | **Observed** | **Mr(expt)** | **Mr(calc)** | **Score** | **Exp Value** | **Peptide** |
|  |  |  | 764.9059 | 1527.7973 | 1527.7933 | 49 | 0.00035 | R.FLTGPLNLNDPEAK.C |
|  |  |  | 625.8679 | 1250.3672 | 1250.3639 | 90 | 7e-008 | K.ILGDINSDFTR.V |
| Proactivator polypeptide | P07602 | 118 | **Observed** | **Mr(expt)** | **Mr(calc)** | **Score** | **Exp Value** | **Peptide** |
|  |  |  | 507.7973 | 1013.5801 | 1013.5757 | 40 | 0.0046 | K.QEILAALEK.G |
|  |  |  | 705.2021 | 1408.5188 | 1408.5167 | 56 | 7.4e-005 | K.DNATEEEILVYL.E |
|  |  |  | 740.9512 | 1481.7474 | 1481.7459 | 79 | 3.7e-007 | R.LGPGMADICKNYIS.Q |
| Cathepsin B | A1E295 | 119 | **Observed** | **Mr(expt)** | **Mr(calc)** | **Score** | **Exp Value** | **Peptide** |
|  |  |  | 498.7591 | 995.5035 | 995.4997 | 100 | 5.4e-010 | K.EIMAEIYK.N |
|  |  |  | 960.8625 | 1921.1148 | 1921.1129 | 53 | 0.00023 | K.SFDAREQWPNCPTIKE.I |
| Ceroid-lipofuscinosis neuronal protein 5 | Q5JZQ9 | 127 | **Observed** | **Mr(expt)** | **Mr(calc)** | **Score** | **Exp Value** | **Peptide** |
|  |  |  | 525.2683 | 1048.5220 | 1048.5189 | 87 | 2e-008 | K.GDDVIEVFR.L |
|  |  |  | 990.0025 | 1978.2262 | 1978.2245 | 50 | 0.00021 | R.EFYLFYNFEYWFLP.M |
| Vacuolar protein sorting-associated protein 4B | Q0VD48 | 108 | **Observed** | **Mr(expt)** | **Mr(calc)** | **Score** | **Exp Value** | **Peptide** |
|  |  |  | 515.3208 | 1028.6271 | 1028.6230 | 43 | 0.00065 | K.AIDLASKAAQ.E |
|  |  |  | 712.3409 | 1422.6671 | 1422.6627 | 82 | 1.4e-007 | K.NLFQLARENKPSIIFID.E |
|  |  |  | 795.4054 | 1588.7963 | 1588.7919 | 42 | 0.0021 | R.AAMFKLHLGTTQNSLTE.A |
| Tissue alpha-L-fucosidase | Q2KIM0 | 189 | **Observed** | **Mr(expt)** | **Mr(calc)** | **Score** | **Exp Value** | **Peptide** |
|  |  |  | 643.8618 | 1285.7090 | 1285.7030 | 76 | 2.5e-008 | K.DGLIVPIFQER.L |
|  |  |  | 732.3651 | 1464.5953 | 1464.5925 | 71 | 8.6e-008 | K.DVGPHRDLVGELGT.A |
|  |  |  | 1044.4351 | 2088.3057 | 2088.3021 | 82 | 6.4e-009 | R.RDMEMADITNESTIISEL.V |
| Palmitoyl-protein thioesterase 1 | Q8HXW6 | 89 | **Observed** | **Mr(expt)** | **Mr(calc)** | **Score** | **Exp Value** | **Peptide** |
|  |  |  | 701.4242 | 1400.8339 | 1400.8279 | 84 | 2.1e-008 | K.IPGIYVLSLEIGK.T |
|  |  |  | 933.4167 | 1866.0469 | 1866.0452 | 37 | 0.0047 | R.SGQAKETIPLQETSLYT.Q |
| Legumain | Q4R4T8 | 146 | **Observed** | **Mr(expt)** | **Mr(calc)** | **Score** | **Exp Value** | **Peptide** |
|  |  |  | 969.4883 | 1936.9620 | 1936.9531 | 70 | 1.5e-007 | K.DYTGEDVTPQNFLAVLR.G |
|  |  |  | 1017.6834 | 2035.1702 | 2035.1679 | 35 | 0.001 | R.ESSYACYYDEKRSTYLG.D |
|  |  |  | 832.0069 | 1663.8247 | 1663.8219 | 87 | 4.8e-009 | k.Lmntndleesrqlt.e |
| Beta-glucuronidase | O18835 | 92 | **Observed** | **Mr(expt)** | **Mr(calc)** | **Score** | **Exp Value** | **Peptide** |
|  |  |  | 389.2179 | 776.4212 | 776.4181 | 21 | 0.0052 | K.DFNLLR.W |
|  |  |  | 467.7502 | 933.4859 | 933.4821 | 29 | 0.0091 | R.WLGANAFR.T |
|  |  |  | 537.8048 | 1073.5951 | 1073.5910 | 29 | 0.00058 | K.GFDWPLLVK.D |
|  |  |  | 554.7772 | 1107.5398 | 1107.5349 | 48 | 0.00069 | K.DLDGLWSFR.A |
|  |  |  | 664.8278 | 1327.6410 | 1327.6350 | 46 | 0.00073 | R.SFVGWVWYER.E |
| N-acetylgalactosamine-6-sulfatase | Q32KH5 | 134 | **Observed** | **Mr(expt)** | **Mr(calc)** | **Score** | **Exp Value** | **Peptide** |
|  |  |  | 535.8338 | 1069.6531 | 1069.6495 | 52 | 0.00011 | K.ILSLLQDLR.I |
|  |  |  | 649.8419 | 1299.4057 | 1299.4032 | 45 | 3e-005 | R.HARNAYTPQEI.V |
|  |  |  | 942.2639 | 1884.1755 | 1884.1726 | 79 | 1.6e-008 | K.LPLIFHLGRDPGERFP.L |
| Dipeptidyl peptidase 2 | Q9EPB1 | 110 | **Observed** | **Mr(expt)** | **Mr(calc)** | **Score** | **Exp Value** | **Peptide** |
|  |  |  | 584.3145 | 1166.6144 | 1166.6084 | 51 | 0.00032 | K.DLTQLFGFAR.N |
|  |  |  | 817.0157 | 1633.7448 | 1633.7417 | 81 | 1.4e-008 | K.MGEGPIFFYTGNEGD.I |
| Ras-related protein Rab-21 | P55745 | 206 | **Observed** | **Mr(expt)** | **Mr(calc)** | **Score** | **Exp Value** | **Peptide** |
|  |  |  | 596.2893 | 1190.5639 | 1190.5601 | 71 | 2.3e-006 | R.MIETAQVDER.A |
|  |  |  | 604.2865 | 1206.5584 | 1206.5550 | 65 | 8.2e-006 | R.MIETAQVDER.A |
|  |  |  | 602.2979 | 1803.8718 | 1803.8639 | 55 | 7e-005 | R.HVSIQEAESYAESVGAK.H |
|  |  |  | 1065.5008 | 2128.9870 | 2128.9801 | 106 | 2.6e-010 | R.DSNGAILVYDITDEDSFQK.V |
| ADP-ribosylation factor 6 | Q007T5 | 118 | **Observed** | **Mr(expt)** | **Mr(calc)** | **Score** | **Exp Value** | **Peptide** |
|  |  |  | 551.3222 | 1100.6299 | 1100.6263 | 37 | 0.0059 | R.ILMLGLDAAGK.T |
|  |  |  | 559.3365 | 1116.6584 | 1116.6543 | 55 | 5.9e-005 | R.DAIILIFANK.Q |
|  |  |  | 632.8042 | 1263.5939 | 1263.5884 | 55 | 0.00011 | K.FNVWDVGGQDK.I |
|  |  |  | 1127.6055 | 2253.1964 | 2253.1893 | 52 | 5.3e-005 | K.LGQSVTTIPTVGFNVETVTYK.N |
| Ras-related protein Rap-2a | Q06AU2 | 100 | **Observed** | **Mr(expt)** | **Mr(calc)** | **Score** | **Exp Value** | **Peptide** |
|  |  |  | 674.8614 | 1347.7082 | 1347.7034 | 85 | 1.1e-007 | K.ASVDELFAEIVR.Q |
|  |  |  | 684.8839 | 1369.5745 | 1369.5714 | 45 | 0.0013 | K.SALTVQFVTGSFI.E |
| Ras-related protein Rab-11A | P62490 | 112 | **Observed** | **Mr(expt)** | **Mr(calc)** | **Score** | **Exp Value** | **Peptide** |
|  |  |  | 472.7473 | 943.4801 | 943.4763 | 38 | 0.0079 | R.AITSAYYR.G |
|  |  |  | 522.3106 | 1042.6066 | 1042.6023 | 57 | 6.9e-005 | K.VVLIGDSGVGK.S |
|  |  |  | 580.7898 | 1159.5650 | 1159.5622 | 29 | 0.053 | K.HLTYENVER.W |
|  |  |  | 637.8124 | 1273.6102 | 1273.6051 | 55 | 8.4e-005 | K.AQIWDTAGQER.Y |
|  |  |  | 645.3802 | 1288.7458 | 1288.7391 | 59 | 1.7e-005 | R.GAVGALLVYDIAK.H |
| Ras-related protein Rab-8A | P61007 | 150 | **Observed** | **Mr(expt)** | **Mr(calc)** | **Score** | **Exp Value** | **Peptide** |
|  |  |  | 536.3264 | 1070.6382 | 1070.6336 | 57 | 4.3e-005 | K.LLLIGDSGVGK.T |
|  |  |  | 658.8359 | 1315.6572 | 1315.6521 | 52 | 0.00023 | K.LQIWDTAGQER.F |
|  |  |  | 671.3201 | 1340.6255 | 1340.6208 | 63 | 1.2e-005 | R.NIEEHASADVEK.M |
|  |  |  | 790.4181 | 1578.8217 | 1578.8154 | 58 | 4e-005 | K.ANINVENAFFTLAR.D |
| Ras-related protein Rab-8B | Q5REC9 | 144 | **Observed** | **Mr(expt)** | **Mr(calc)** | **Score** | **Exp Value** | **Peptide** |
|  |  |  | 536.3264 | 1070.6382 | 1070.6336 | 57 | 4.3e-005 | K.LLLIGDSGVGK.T |
|  |  |  | 658.8359 | 1315.6572 | 1315.6521 | 52 | 0.00023 | K.LQIWDTAGQER.F |
|  |  |  | 786.3917 | 1570.7688 | 1570.7627 | 83 | 1.1e-007 | K.SSTNVEEAFFTLAR.D |
| Ras-related protein Rab-14 | Q5R8Z8 | 65 | **Observed** | **Mr(expt)** | **Mr(calc)** | **Score** | **Exp Value** | **Peptide** |
|  |  |  | 401.7158 | 801.4170 | 801.3981 | 21 | 0.00075 | K.ADLEAQR.D |
|  |  |  | 658.8359 | 1315.6572 | 1315.6521 | 52 | 0.00023 | K.LQIWDTAGQER.F |
| Vesicle-associated membrane protein 7 | Q5RF94 | 92 | **Observed** | **Mr(expt)** | **Mr(calc)** | **Score** | **Exp Value** | **Peptide** |
|  |  |  | 480.2343 | 958.4540 | 958.4509 | 31 | 0.003 | R.FQTTYGSR.A |
|  |  |  | 548.2979 | 1094.5812 | 1094.5760 | 36 | 0.00091 | R.AFNFLNEIK.K |
|  |  |  | 722.0656 | 2163.1750 | 2163.1674 | 53 | 3e-005 | R.LELLIDKTENLVDSSVTFK.T |
| Ragulator complex protein LAMTOR3 | Q5R3Z6 | 96 | **Observed** | **Mr(expt)** | **Mr(calc)** | **Score** | **Exp Value** | **Peptide** |
|  |  |  | 608.8348 | 1215.6551 | 1215.6499 | 57 | 9e-005 | K.ELAPLFEELR.R |
|  |  |  | 425.8443 | 850.9671 | 850.9639 | 60 | 3.3e-006 | K.LPSVEGLH.A |
| ADP-ribosylation factor-like protein 8A | Q8VEH3 | 251 | **Observed** | **Mr(expt)** | **Mr(calc)** | **Score** | **Exp Value** | **Peptide** |
|  |  |  | 411.2330 | 820.4515 | 820.4483 | 23 | 0.22 | K.LLDWFK.A |
|  |  |  | 521.2790 | 1040.5434 | 1040.5403 | 61 | 3.5e-005 | K.LWDIGGQPR.F |
|  |  |  | 524.2678 | 1046.5210 | 1046.5179 | 56 | 0.00014 | K.MNLSAIQDR.E |
|  |  |  | 532.2657 | 1062.5168 | 1062.5128 | 65 | 1.5e-005 | K.MNLSAIQDR.E |
|  |  |  | 565.9494 | 1694.8264 | 1694.8185 | 39 | 0.003 | R.GVSAIVYMVDAADQEK.I |
|  |  |  | 856.4170 | 1710.8194 | 1710.8134 | 94 | 1e-008 | R.GVSAIVYMVDAADQEK.I |
|  |  |  | 856.9271 | 1711.8395 | 1711.8338 | 61 | 1.8e-005 | K.EEMELTLVGLQYSGK.T |
|  |  |  | 694.3755 | 2080.1046 | 2080.0953 | 39 | 0.0016 | K.EKDNIDITLQWLIQHSK.S |
|  |  |  | 851.4921 | 2551.4544 | 2551.4486 | 58 | 3.6e-006 | K.NELHNLLDKPQLQGIPVLVLGNK.R |
| ADP-ribosylation factor-like protein 8B | Q2KI07 | 286 | **Observed** | **Mr(expt)** | **Mr(calc)** | **Score** | **Exp Value** | **Peptide** |
|  |  |  | 425.2363 | 848.4581 | 848.4545 | 27 | 0.14 | R.LLDWFR.S |
|  |  |  | 521.2790 | 1040.5434 | 1040.5403 | 61 | 3.5e-005 | K.IWDIGGQPR.F |
|  |  |  | 524.2678 | 1046.5210 | 1046.5179 | 56 | 0.00014 | K.MNLSAIQDR.E |
|  |  |  | 532.2657 | 1062.5168 | 1062.5128 | 65 | 1.5e-005 | K.MNLSAIQDR.E |
|  |  |  | 754.3854 | 1506.7563 | 1506.7500 | 78 | 4.3e-007 | R.GVNAIVYMIDAADR.E |
|  |  |  | 762.3822 | 1522.7497 | 1522.7450 | 82 | 2.1e-007 | R.GVNAIVYMIDAADR.E |
|  |  |  | 856.9271 | 1711.8395 | 1711.8338 | 61 | 1.8e-005 | K.EEMELTLVGLQYSGK.T |
|  |  |  | 588.9723 | 1763.8949 | 1763.8876 | 27 | 0.056 | R.GVNAIVYMIDAADREK.I |
|  |  |  | 694.3755 | 2080.1046 | 2080.0953 | 39 | 0.0016 | K.EKDNIDITLQWLIQHSK.S |
|  |  |  | 851.4921 | 2551.4544 | 2551.4486 | 58 | 3.6e-006 | R.NELHNLLDKPQLQGIPVLVLGNK.R |
| Niemann-pick C1 protein | O15118 | 108 | **Observed** | **Mr(expt)** | **Mr(calc)** | **Score** | **Exp Value** | **Peptide** |
|  |  |  | 448.7491 | 895.4835 | 895.4803 | 27 | 0.0086 | K.EFINFVK.N |
|  |  |  | 488.2424 | 974.4702 | 974.4669 | 36 | 0.0011 | R.SIEDELNR.E |
|  |  |  | 768.3851 | 1534.7557 | 1534.7490 | 67 | 6.8e-006 | R.FLPMFLSDNPNPK.C |
|  |  |  | 537.2865 | 1608.8376 | 1608.8300 | 28 | 0.0034 | R.AQAWEKEFINFVK.N |
| Ras-related protein Rab-27A | Q1HE58 | 136 | **Observed** | **Mr(expt)** | **Mr(calc)** | **Score** | **Exp Value** | **Peptide** |
|  |  |  | 521.7897 | 1041.5648 | 1041.5607 | 21 | 0.35 | K.SWIPEGVVR.S |
|  |  |  | 532.2951 | 1062.5757 | 1062.5710 | 49 | 0.00046 | K.FLALGDSGVGK.T |
|  |  |  | 584.8241 | 1167.6336 | 1167.6288 | 50 | 0.00026 | K.FITTVGIDFR.E |
|  |  |  | 637.8191 | 1273.6236 | 1273.6190 | 65 | 1.2e-005 | K.TSVLYQYTDGK.F |
|  |  |  | 522.9412 | 1565.8017 | 1565.7950 | 22 | 0.17 | R.IHLQLWDTAGQER.F |
|  |  |  | 820.4181 | 2458.2325 | 2458.2202 | 69 | 1.5e-006 | R.DAMGFLLLFDLTNEQSFLNVR.N |
| Ragulator complex protein LAMTOR1 | Q3T0D8 | 127 | **Observed** | **Mr(expt)** | **Mr(calc)** | **Score** | **Exp Value** | **Peptide** |
|  |  |  | 584.3370 | 1166.6594 | 1166.6547 | 48 | 0.0004 | K.LLLDPSSPPTK.A |
|  |  |  | 694.8882 | 1387.7619 | 1387.7558 | 104 | 9.9e-010 | R.TDEQALLSSILAK.T |
|  |  |  | 895.3933 | 2683.1582 | 2683.1490 | 35 | 0.00071 | K.TASNIIDVSAADSQGMEQHEYMDR.A |
| CD63 antigen | Q76B49 | 85 | **Observed** | **Mr(expt)** | **Mr(calc)** | **Score** | **Exp Value** | **Peptide** |
|  |  |  | 351.2103 | 701.4134 | 701.4131 | 49 | 0.0002 | K.IGGWLR.S |
|  |  |  | 971.4561 | 1942.4948 | 1942.4925 | 57 | 7.3e-005 | K.FLLYVLLLAFCACAVGLI.A |
| Syntaxin-7 | O70439 | 106 | **Observed** | **Mr(expt)** | **Mr(calc)** | **Score** | **Exp Value** | **Peptide** |
|  |  |  | 547.2871 | 1092.5597 | 1092.5564 | 38 | 0.0087 | K.QQYTNQLAK.E |
|  |  |  | 555.2608 | 1108.5070 | 1108.5037 | 47 | 0.00062 | R.VSGGFPEDSSK.E |
|  |  |  | 724.8565 | 1447.6984 | 1447.6943 | 22 | 0.22 | K.EFGSLPTTPSEQR.Q |
|  |  |  | 847.9217 | 1693.8288 | 1693.8233 | 81 | 2.2e-007 | R.QLEADIMDINEIFK.D |
| Charged multivesicular body protein 4b | Q9D8B3 | 81 | **Observed** | **Mr(expt)** | **Mr(calc)** | **Score** | **Exp Value** | **Peptide** |
|  |  |  | 501.7784 | 1001.5422 | 1001.5393 | 32 | 0.0036 | K.IEQELTAAK.K |
|  |  |  | 773.3945 | 1544.7745 | 1544.7682 | 74 | 1.3e-006 | R.EALENANTNTEVLK.N |
| MLN64 N-terminal domain homolog | Q9DCI3 | 67 | **Observed** | **Mr(expt)** | **Mr(calc)** | **Score** | **Exp Value** | **Peptide** |
|  |  |  | 572.3239 | 1142.6333 | 1142.6295 | 44 | 0.0017 | R.LLLVQDASER.A |
|  |  |  | 657.3221 | 1312.6296 | 1312.6259 | 44 | 0.0012 | K.VLPQEAEEENR.L |
| Calreticulin | P28491 | 54 | **Observed** | **Mr(expt)** | **Mr(calc)** | **Score** | **Exp Value** | **Peptide** |
|  |  |  | 719.8151 | 1438.6229 | 1438.6226 | 41 | 2.4e-05 | K.EQFLDGDGWTDR.W |
|  |  |  | 414.2241 | 827.4408 | 827.4401 | 30 | 0.0027 | R.FYALSAR.F |
| Cytoskeleton-associated protein 4 | Q8BMK4 | 80 | **Observed** | **Mr(expt)** | **Mr(calc)** | **Score** | **Exp Value** | **Peptide** |
|  |  |  | 429.2598 | 856.5050 | 856.5018 | 23 | 0.3 | K.LQNEILK.D |
|  |  |  | 445.2548 | 888.4949 | 888.4916 | 27 | 0.12 | R.LSSLDNLK.S |
|  |  |  | 626.8451 | 1251.6756 | 1251.6711 | 56 | 6.8e-005 | R.TAVDSLVAYSVK.I |
|  |  |  | 681.3328 | 1360.6511 | 1360.6470 | 54 | 0.00011 | K.IETNENNLESAK.G |
| Rab-6A | Q5RAV6 | 72 | **Observed** | **Mr(expt)** | **Mr(calc)** | **Score** | **Exp Value** | **Peptide** |
|  |  |  | 402.2077 | 802.4008 | 802.3974 | 35 | 0.025 | K.WIDDVR.T |
|  |  |  | 658.8359 | 1315.6572 | 1315.6521 | 52 | 0.00023 | R.LQLWDTAGQER.F |
|  |  |  | 699.3569 | 1396.6992 | 1396.6908 | 47 | 0.0006 | K.ELNVMFIETSAK.A |
| Rab-6B | A6QR46 | 74 | **Observed** | **Mr(expt)** | **Mr(calc)** | **Score** | **Exp Value** | **Peptide** |
|  |  |  | 588.8414 | 1175.6683 | 1175.6550 | 38 | 0.0014 | K.LVFLGEQSVGK.T |
|  |  |  | 658.8411 | 1315.6677 | 1315.6521 | 67 | 2.9e-006 | R.LQLWDTAGQER.F |
| Nicastrin | Q92542 | 184 | **Observed** | **Mr(expt)** | **Mr(calc)** | **Score** | **Exp Value** | **Peptide** |
|  |  |  | 501.2943 | 1000.5741 | 1000.5706 | 62 | 9.9e-006 | K.ADVLFIAPR.E |
|  |  |  | 862.5619 | 1724.9630 | 1724.9600 | 81 | 1e-007 | K.PINTTGTLKPDDRVVV.A |
|  |  |  | 1050.9937 | 2102.2939 | 2102.2921 | 53 | 2.1e-005 | R.TSLELWMHTDPVSQKNES.V |
|  |  |  | 1019.9986 | 2040.1632 | 2040.1614 | 31 | 0.0046 | K.YYQSIYDTAENINVSYP.E |
|  |  |  | 601.7419 | 1203.4238 | 1203.4219 | 73 | 4.9e-007 | R.CVRSTARLARA.L |
| ADP-ribosylation factor 4 | Q3SZF2 | 81 | **Observed** | **Mr(expt)** | **Mr(calc)** | **Score** | **Exp Value** | **Peptide** |
|  |  |  | 544.3168 | 1086.6190 | 1086.6107 | 42 | 0.0011 | R.ILMVGLDAAGK.T |
|  |  |  | 552.3168 | 1102.6190 | 1102.6056 | 30 | 0.001 | R.ILMVGLDAAGK.T |
|  |  |  | 572.8021 | 1143.5896 | 1143.5771 | 39 | 0.0018 | R.IQEGAEELQK.M |
| Golgi-associated plant pathogenesis-related protein 1 | Q9CYL5 | 107 | **Observed** | **Mr(expt)** | **Mr(calc)** | **Score** | **Exp Value** | **Peptide** |
|  |  |  | 496.2658 | 990.5170 | 990.5134 | 34 | 0.0024 | K.QFNNEVLK.A |
|  |  |  | 727.3525 | 1452.6904 | 1452.6844 | 99 | 3.6e-009 | R.EAQQYSEALASTR.I |
| Ras-related protein Rab-2A | Q4R4X6 | 108 | **Observed** | **Mr(expt)** | **Mr(calc)** | **Score** | **Exp Value** | **Peptide** |
|  |  |  | 660.3721 | 1318.7297 | 1318.7245 | 54 | 9.7e-005 | R.GAAGALLVYDITR.R |
|  |  |  | 747.8782 | 1493.7419 | 1493.7362 | 87 | 7e-008 | K.TASNVEEAFINTAK.E |
| Complement C3 | Q2UVX4 | 212 | **Observed** | **Mr(expt)** | **Mr(calc)** | **Score** | **Exp Value** | **Peptide** |
|  |  |  | 417.2493 | 832.4840 | 832.4807 | 33 | 0.016 | R.LPYSVVR.N |
|  |  |  | 341.8675 | 1022.5806 | 1022.5760 | 24 | 0.091 | K.IGLHEVEVK.A |
|  |  |  | 582.8300 | 1163.6454 | 1163.6411 | 46 | 0.00064 | R.HQQTITIPAR.S |
|  |  |  | 670.3667 | 1338.7189 | 1338.7143 | 22 | 0.17 | R.VPINDGNGEAILK.R |
|  |  |  | 685.8720 | 1369.7294 | 1369.7242 | 34 | 0.012 | K.TIYTPGSTVLYR.V |
|  |  |  | 686.4184 | 1370.8222 | 1370.8173 | 38 | 0.00076 | R.SSVAVPYVIVPLK.I |
|  |  |  | 968.4873 | 1934.9601 | 1934.9513 | 54 | 7.5e-005 | K.EYVLPSFEVQLEPEEK.F |
|  |  |  | 1068.9955 | 2135.9764 | 2135.9688 | 94 | 4.6e-009 | K.AYYEDSPQQVFSAEFEVK.E |
|  |  |  | 1119.1475 | 2236.2804 | 2236.2719 | 79 | 2e-008 | K.LLPVGQTVFITIETPDGIPVK.R |
| Apolipoprotein E | Q03247 | 132 | **Observed** | **Mr(expt)** | **Mr(calc)** | **Score** | **Exp Value** | **Peptide** |
|  |  |  | 401.2283 | 800.4421 | 800.4392 | 31 | 0.068 | R.LEEVGVR.A |
|  |  |  | 517.2763 | 1032.5381 | 1032.5352 | 39 | 0.007 | R.LQAEAFQAR.L |
|  |  |  | 711.3569 | 1420.6993 | 1420.6946 | 78 | 5.1e-007 | R.LAVYQAGASEGAER.S |
|  |  |  | 847.4095 | 1692.8045 | 1692.7988 | 76 | 5.8e-007 | R.SEVQAMLGQSTEELR.A |
| CD166 antigen | O46634 | 132 | **Observed** | **Mr(expt)** | **Mr(calc)** | **Score** | **Exp Value** | **Peptide** |
|  |  |  | 516.2565 | 1030.4985 | 1030.4931 | 22 | 0.31 | K.DLGNLEENK.K |
|  |  |  | 588.3213 | 1174.6280 | 1174.6234 | 47 | 0.00093 | K.APFLETEQLK.K |
|  |  |  | 628.8117 | 1255.6089 | 1255.6044 | 56 | 6.5e-005 | R.SSNTYTLTDVR.R |
|  |  |  | 652.3688 | 1302.7230 | 1302.7183 | 33 | 0.014 | K.APFLETEQLKK.L |
|  |  |  | 471.5777 | 1411.7112 | 1411.7056 | 26 | 0.081 | R.SSNTYTLTDVRR.N |
|  |  |  | 777.4904 | 1552.9661 | 1552.9593 | 75 | 2.9e-008 | K.VLQPLEGVVVLIFK.K |
|  |  |  | 518.9769 | 1553.9090 | 1553.9028 | 27 | 0.007 | R.ESLTLIVEGKPQIK.M |
|  |  |  | 883.1302 | 2646.3687 | 2646.3575 | 34 | 0.0034 | K.SMIASTAITVHYLDLSLNPSGEVTK.Q |
| Alpha-2-macroglobulin | Q7SIH1 | 524 | **Observed** | **Mr(expt)** | **Mr(calc)** | **Score** | **Exp Value** | **Peptide** |
|  |  |  | 383.2151 | 764.4157 | 764.4068 | 29 | 0.027 | R.GEAFTLK.A |
|  |  |  | 431.7529 | 861.4911 | 861.4807 | 37 | 0.0036 | K.VSESLSLK.L |
|  |  |  | 522.7658 | 1043.5171 | 1043.5036 | 83 | 7.7e-008 | R.YGAATFTSAR.K |
|  |  |  | 554.8046 | 1107.5947 | 1107.5812 | 34 | 0.0051 | R.SLFTDVVAEK.D |
|  |  |  | 558.8144 | 1115.6143 | 1115.5975 | 29 | 0.013 | R.QTVSWAVTPK.S |
|  |  |  | 566.3290 | 1130.6435 | 1130.6295 | 54 | 3.6e-005 | R.STGTLLNNAIK.G |
|  |  |  | 568.3206 | 1134.6266 | 1134.6132 | 75 | 1.3e-006 | K.GATEITTTITK.L |
|  |  |  | 572.8300 | 1143.6455 | 1143.5958 | 26 | 0.029 | R.PASNMAIVDVK.M |
|  |  |  | 605.8314 | 1209.6481 | 1209.6353 | 37 | 0.002 | K.LPPNVVEESAR.A |
|  |  |  | 625.8482 | 1249.6819 | 1249.6667 | 50 | 0.00011 | K.LSFVTVDSNLR.R |
|  |  |  | 644.3559 | 1286.6972 | 1286.6830 | 61 | 7.7e-006 | R.DTGLGLSPTASLR.V |
|  |  |  | 695.3873 | 1388.7601 | 1388.7414 | 65 | 2.5e-006 | K.ELVFYYLIMAK.G |
|  |  |  | 703.3847 | 1404.7548 | 1404.7363 | 81 | 6.9e-008 | K.ELVFYYLIMAK.G |
|  |  |  | 711.3835 | 1420.7524 | 1420.7350 | 67 | 1.9e-006 | R.SNSFVYLEPLPR.E |
|  |  |  | 716.8702 | 1431.7258 | 1431.7093 | 73 | 6.2e-007 | K.IQEEGTEVELTGK.G |
|  |  |  | 762.4092 | 1522.8039 | 1522.7814 | 53 | 4.8e-005 | R.ASFSVLGDILGSAMR.N |
|  |  |  | 951.0338 | 1900.0531 | 1900.0306 | 52 | 2e-005 | R.LLIYAILPDGEVVGDSAR.Y |
|  |  |  | 668.0097 | 2001.0072 | 2000.9844 | 39 | 0.00077 | K.GSGGTAEHPFTVEEFVLPK.F |
|  |  |  | 773.7478 | 2318.2217 | 2318.1947 | 50 | 3.3e-005 | R.GYIFIDEAHITEALTWLAQK.Q |
| Factor XⅡa inhibitor | P50448 | 220 | **Observed** | **Mr(expt)** | **Mr(calc)** | **Score** | **Exp Value** | **Peptide** |
|  |  |  | 512.7451 | 1023.4756 | 1023.4621 | 50 | 0.0001 | K.DTFAEASQR.L |
|  |  |  | 556.8095 | 1111.6045 | 1111.5914 | 38 | 0.0013 | R.LYQDFSVLK.K |
|  |  |  | 579.8104 | 1157.6062 | 1157.5928 | 62 | 1e-005 | R.LLDSLPEDTR.L |
|  |  |  | 613.4051 | 1224.7956 | 1224.7805 | 57 | 2.1e-006 | R.LILLNAVALSAK.W |
|  |  |  | 839.9473 | 1677.8801 | 1677.8573 | 124 | 3.1e-012 | R.SAEAVLGEALTDFSLR.L |
| Sulfated glycoprotein 1 | P10960 | 95 | **Observed** | **Mr(expt)** | **Mr(calc)** | **Score** | **Exp Value** | **Peptide** |
|  |  |  | 867.4770 | 1732.9394 | 1732.9321 | 82 | 8.6e-008 | K.EVVDSYLPVILDMIK.G |
|  |  |  | 583.9855 | 1748.9348 | 1748.9270 | 41 | 0.0013 | K.EVVDSYLPVILDMIK.G |
| Histone H2B type 3-B | Q8CGP0 | 285 | **Observed** | **Mr(expt)** | **Mr(calc)** | **Score** | **Exp Value** | **Peptide** |
|  |  |  | 477.3071 | 952.5996 | 952.5957 | 22 | 0.048 | R.LLLPGELAK.H |
|  |  |  | 576.2870 | 1150.5594 | 1150.5546 | 22 | 0.24 | K.ESYSIYVYK.V |
|  |  |  | 640.3344 | 1278.6542 | 1278.6496 | 59 | 4e-005 | R.KESYSIYVYK.V |
|  |  |  | 872.4192 | 1742.8239 | 1742.8120 | 87 | 3.5e-008 | K.AMGIMNSFVNDIFER.I |
|  |  |  | 880.4139 | 1758.8133 | 1758.8069 | 84 | 6.9e-008 | K.AMGIMNSFVNDIFER.I |
|  |  |  | 880.4148 | 1758.8150 | 1758.8069 | 107 | 3.6e-010 | K.AMGIMNSFVNDIFER.I |
|  |  |  | 888.4122 | 1774.8098 | 1774.8018 | 74 | 7.6e-007 | K.AMGIMNSFVNDIFER.I |
| Core histone macro-H2A.1 | O75367 | 119 | **Observed** | **Mr(expt)** | **Mr(calc)** | **Score** | **Exp Value** | **Peptide** |
|  |  |  | 493.3078 | 984.6010 | 984.5968 | 28 | 0.032 | K.QTAAQLILK.A |
|  |  |  | 602.8348 | 1203.6550 | 1203.6499 | 65 | 1.4e-005 | K.EFVEAVLELR.K |
|  |  |  | 662.7202 | 1985.1388 | 1985.1310 | 33 | 0.0015 | K.GVTIASGGVLPNIHPELLAK.K |
|  |  |  | 1064.0116 | 2126.0086 | 2126.0015 | 66 | 4.2e-006 | K.AASADSTTEGTPADGFTVLSTK.S |
|  |  |  | 997.8712 | 2990.5917 | 2990.5786 | 44 | 0.00018 | R.IGVGAPVYMAAVLEYLTAEILELAGNAAR.D |
| Serpin A3-2 | A2I7M9 | 255 | **Observed** | **Mr(expt)** | **Mr(calc)** | **Score** | **Exp Value** | **Peptide** |
|  |  |  | 561.3162 | 1120.6178 | 1120.6128 | 66 | 7e-006 | K.DTQSIIFLGK.V |
|  |  |  | 390.2348 | 1167.6825 | 1167.6764 | 30 | 0.012 | R.RIHELYLPK.F |
|  |  |  | 630.8582 | 1259.7019 | 1259.6973 | 63 | 1.3e-005 | R.GSTLTEILEGLK.F |
|  |  |  | 751.4219 | 1500.8292 | 1500.8228 | 77 | 3.8e-007 | R.TELVLVNYIYFK.A |
|  |  |  | 867.9584 | 1733.9022 | 1733.8948 | 93 | 1.1e-008 | K.SNYELNDILSQLGIR.K |
|  |  |  | 1029.5032 | 2056.9918 | 2056.9850 | 40 | 0.0016 | K.TVEVPMMTLDLETPYFR.D |
|  |  |  | 1037.5010 | 2072.9874 | 2072.9799 | 56 | 3.9e-005 | K.TVEVPMMTLDLETPYFR.D |
|  |  |  | 1117.5480 | 2233.0814 | 2233.0691 | 34 | 0.0063 | K.FIEDAQVLYSSEAFPTNFR.D |
|  |  |  | 901.7978 | 2702.3717 | 2702.3592 | 31 | 0.0065 | K.LLDKFIEDAQVLYSSEAFPTNFR.D |
| Plectin | Q15149 | 181 | **Observed** | **Mr(expt)** | **Mr(calc)** | **Score** | **Exp Value** | **Peptide** |
|  |  |  | 501.2685 | 1000.5224 | 1000.5189 | 31 | 0.04 | K.AQAEVEGLGK.G |
|  |  |  | 508.2820 | 1014.5495 | 1014.5458 | 71 | 4.7e-006 | R.LSVAAQEAAR.L |
|  |  |  | 555.8134 | 1109.6123 | 1109.6080 | 29 | 0.037 | K.AQLEPVASPAK.K |
|  |  |  | 565.3035 | 1128.5924 | 1128.5887 | 31 | 0.033 | R.NLVDNITGQR.L |
|  |  |  | 572.3049 | 1142.5953 | 1142.5931 | 49 | 0.00054 | R.LQAEEVAQQK.S |
|  |  |  | 588.7371 | 1175.4596 | 1175.4553 | 43 | 0.00011 | R.GYFDEEMNR.V |
|  |  |  | 595.8445 | 1189.6744 | 1189.6707 | 41 | 0.0017 | R.LLFNDVQTLK.D |
|  |  |  | 783.9497 | 1565.8848 | 1565.8777 | 92 | 5e-009 | R.APVPASELLASGVLSR.A |
|  |  |  | 769.7209 | 2306.1408 | 2306.1331 | 39 | 0.0023 | K.GFFDPNTHENLTYLQLLER.C |
| Synaptic vesicle membrane protein VAT-1 homolog | Q99536 | 117 | **Observed** | **Mr(expt)** | **Mr(calc)** | **Score** | **Exp Value** | **Peptide** |
|  |  |  | 597.8424 | 1193.6702 | 1193.6656 | 37 | 0.0039 | K.VLLVPGPEKEN.- |
|  |  |  | 740.4004 | 1478.7862 | 1478.7803 | 56 | 7.4e-005 | K.VVTYGMANLLTGPK.R |
|  |  |  | 795.8917 | 1589.7688 | 1589.7607 | 79 | 3.7e-007 | K.GVDIVMDPLGGSDTAK.G |
| Prohibitin-2 | Q5RB19 | 110 | **Observed** | **Mr(expt)** | **Mr(calc)** | **Score** | **Exp Value** | **Peptide** |
|  |  |  | 630.3794 | 1258.7443 | 1258.7397 | 62 | 5.5e-006 | K.LLLGAGAVAYGVR.E |
|  |  |  | 862.4672 | 1722.9199 | 1722.9133 | 43 | 0.00093 | R.IPWFQYPIIYDIR.A |
|  |  |  | 618.6701 | 1852.9884 | 1852.9796 | 46 | 0.00037 | R.IGGVQQDTILAEGLHFR.I |
|  |  |  | 747.4012 | 2239.1818 | 2239.1736 | 46 | 0.00029 | R.AKDFSLILDDVAITELSFSR.E |
