# Supplementary material for: Proteomic Analysis of the Ehrlichia chaffeensis Phagosome in Cultured DH82 Cells
Source: PLoS One. 2014 Feb 18;9(2):e88461. doi: 10.1371/journal.pone.0088461 (PMC3928192; doi:10.1371/journal.pone.0088461)
Supplement: Table S6 — Mascot results for proteins detected in ECVs that were not detectable in any latex bead phagolysosomes. (DOCX) [file pone.0088461.s006.docx]

**Supplemental Table S6: Mascot results for proteins detected in ECVs that were not detectable in any latex bead phagolysosomes**

| **Protein identified** | **Accession No.** | **Protein score** | **Mascot results** | | | | | |
| --- | --- | --- | --- | --- | --- | --- | --- | --- |
| Moesin | Q2HJ49 | 87 | **Observed** | **Mr(expt)** | **Mr(calc)** | **Score** | **Exp Value** | **Peptide** |
|  |  |  | 447.7780 | 893.5414 | 893.5375 | 33 | 0.0011 | R.LFFLQVK.E |
|  |  |  | 552.7979 | 1103.5813 | 1103.5764 | 51 | 3.1e-005 | K.IGFPWSEIR.N |
|  |  |  | 591.8039 | 1181.5933 | 1181.5869 | 47 | 5.8e-005 | K.APDFVFYAPR.L |
|  |  |  | 694.6752 | 2081.0036 | 2080.9953 | 26 | 0.0035 | K.FYPEDVSEELIQDITQR.L |
| Ras-related protein Ral-B | P11234 | 87 | **Observed** | **Mr(expt)** | **Mr(calc)** | **Score** | **Exp Value** | **Peptide** |
|  |  |  | 509.8600 | 1017.5136 | 1017.5112 | 75 | 5.60e-03 | K.VIMVGSGGVGK.S |
|  |  |  | 927.0016 | 1853.9935 | 1853.9911 | 37 | 0.0031 | K.AEEWGVQYVETSAKTR.A |
| Regulator of G-protein signaling 20 | P79348 | 90 | **Observed** | **Mr(expt)** | **Mr(calc)** | **Score** | **Exp Value** | **Peptide** |
|  |  |  | 357.7487 | 713.4829 | 713.4799 | 79 | 4.8e-008 | R.LSLLLR.A |
|  |  |  | 530.7940 | 1059.5735 | 1059.5673 | 35 | 0.00057 | K.EVSLDSRVR.E |
| Solute carrier family 15 member 2 | P46029 | 140 | **Observed** | **Mr(expt)** | **Mr(calc)** | **Score** | **Exp Value** | **Peptide** |
|  |  |  | 545.3266 | 1089.6459 | 1089.6456 | 72 | 2.4e-07 | K.SLSAFPILGGK.V |
|  |  |  | 527.7251 | 1055.2349 | 1055.2345 | 26 | 0.0032 | K.QIPHMQGNM.I |
|  |  |  | 560.2110 | 1120.2641 | 1120.2637 | 44 | 5.3e-005 | K.PPPEGNIVAQV.V |
|  |  |  | 815.8993 | 1631.7916 | 1631.7909 | 36 | 0.00075 | K.DFSLNLGLLDFGASY.L |
|  |  |  | 1210.0049 | 2419.8532 | 2419.8528 | 40 | 0.00036 | R.LSSPAKKTPPKICGSNYPLSIAF.I |
| Platelet endothelial cell adhesion molecule | Q95242 | 156 | **Observed** | **Mr(expt)** | **Mr(calc)** | **Score** | **Exp Value** | **Peptide** |
|  |  |  | 389.2185 | 777.4297 | 777.4291 | 54 | 1.0e-05 | K.FELNVR.D |
|  |  |  | 788.4123 | 1576.6714 | 1576.6710 | 30 | 0.0042 | K.DDVLFHNVSSTKNT.E |
|  |  |  | 663.8126 | 1327.4712 | 1327.4709 | 51 | 4.3e-005 | R.NGSEAVYSVMATV.E |
|  |  |  | 1102.9270 | 2205.7407 | 2205.7404 | 36 | 3.9e-005 | K.VLRVKVIAPVEEVKLSILLS.E |
|  |  |  | 922.7065 | 1845.0119 | 1845.0113 | 47 | 3.9e-005 | R.HYGYNEDVGNHAMKPL.N |
| Adenylate cyclase type 10 | Q866F4 | 228 | **Observed** | **Mr(expt)** | **Mr(calc)** | **Score** | **Exp Value** | **Peptide** |
|  |  |  | 459.7885 | 917.5623 | 917.5698 | 36 | 0.00063 | R.AVKVNFLK.P |
|  |  |  | 860.5625 | 1721.0729 | 1721.0725 | 29 | 0.0028 | R.AIVRIAAHLPDLIVYG.D |
|  |  |  | 1018.2159 | 2036.3717 | 2036.3711 | 61 | 8.1e-007 | K.EFLMSNCSRVLMYEGLSG.F |
|  |  |  | 531.2159 | 1062.2243 | 1062.2239 | 31 | 0.0012 | R.LALKQNAASF.E |
|  |  |  | 1051.9405 | 2103.5016 | 2103.5013 | 94 | 4.1e-010 | R.IFPYNLISLFLHTHMEK.N |
|  |  |  | 1051.9410 | 2103.5019 | 2103.5013 | 83 | 6.3e-009 | R.IFPYNLISLFLHTHMEK.N |
| EH domain-containing protein 1 | Q5E9R3 | 63 | **Observed** | **Mr(expt)** | **Mr(calc)** | **Score** | **Exp Value** | **Peptide** |
|  |  |  | 414.7342 | 827.4538 | 827.4501 | 31 | 0.00071 | K.DIQSLPR.N |
|  |  |  | 837.4045 | 1672.7945 | 1672.7885 | 50 | 1.4e-005 | R.GYDFAAVLEWFAER.V |
| Signal peptidase complex subunit 3 | Q3SZU5 | 120 | **Observed** | **Mr(expt)** | **Mr(calc)** | **Score** | **Exp Value** | **Peptide** |
|  |  |  | 707.3792 | 1412.7439 | 1412.7412 | 47 | 2.2e-005 | K.NNALNQVVLWDK.I |
|  |  |  | 736.9210 | 1473.6428 | 1473.6424 | 94 | 5.8e-010 | k.gnrnvtltlswnv.v |
| Major facilitator superfamily domain-containing protein 10 | Q0P5M9 | 86 | **Observed** | **Mr(expt)** | **Mr(calc)** | **Score** | **Exp Value** | **Peptide** |
|  |  |  | 662.4071 | 1322.7996 | 1322.7921 | 65 | 3.2e-007 | R.AAADLLSPLALLR.F |
|  |  |  | 485.2576 | 968.5006 | 968.5080 | 44 | 6.9e-005 | K.SFAAFLASR.V |
| Stomatin-like protein 2 | Q32LL2 | 124 | **Observed** | **Mr(expt)** | **Mr(calc)** | **Score** | **Exp Value** | **Peptide** |
|  |  |  | 835.9492 | 1669.8838 | 1669.8635 | 93 | 3.7e-009 | K.AEQINQAAGEASAVLAK.A |
|  |  |  | 1007.5539 | 2013.0932 | 2013.0684 | 58 | 7.3e-006 | R.NTVVLFVPQQEAWVVER.M |
| Cleft lip and palate transmembrane protein 1 | Q2NL17 | 159 | **Observed** | **Mr(expt)** | **Mr(calc)** | **Score** | **Exp Value** | **Peptide** |
|  |  |  | 863.9792 | 1725.9439 | 1725.9342 | 95 | 7.6e-010 | K.ALNTFIDDLFAFVIK.M |
|  |  |  | 806.5304 | 1612.7015 | 1612.7009 | 41 | 0.0003 | R.DPPAETQPQNPPPQP.A |
|  |  |  | 622.7539 | 1245.4711 | 1245.4707 | 70 | 1.6e-007 | K.VALLETNPYLL.A |
| Erlin-2 | Q1RMU4 | 101 | **Observed** | **Mr(expt)** | **Mr(calc)** | **Score** | **Exp Value** | **Peptide** |
|  |  |  | 667.8533 | 1333.6921 | 1333.6877 | 75 | 1e-006 | R.ISEIEDAAFLAR.E |
|  |  |  | 674.8533 | 1347.6920 | 1347.6882 | 59 | 4.2e-005 | K.SVQTTLQTDEVK.N |
| Kinectin | O97961 | 71 | **Observed** | **Mr(expt)** | **Mr(calc)** | **Score** | **Exp Value** | **Peptide** |
|  |  |  | 679.8770 | 1357.7395 | 1357.7340 | 58 | 4.5e-005 | K.SVEELLEAEVLK.V |
|  |  |  | 644.3640 | 1286.7135 | 1286.7082 | 47 | 5.2e-005 | K.SVLAETEGILQK.L |
| ERO1-like protein alpha | A5PJN2 | 68 | **Observed** | **Mr(expt)** | **Mr(calc)** | **Score** | **Exp Value** | **Peptide** |
|  |  |  | 521.7707 | 1041.5268 | 1041.5131 | 38 | 0.00021 | K.LLESDYFR.Y |
|  |  |  | 753.3787 | 1504.7428 | 1504.7257 | 56 | 2.7e-005 | R.LGAVDESLSEETQK.A |
| Dolichyl-diphosphooligosaccharide-protein glycosyltransferase subunit 2 | Q3SZI6 | 113 | **Observed** | **Mr(expt)** | **Mr(calc)** | **Score** | **Exp Value** | **Peptide** |
|  |  |  | 686.8721 | 1371.7296 | 1371.7245 | 82 | 2e-007 | R.SIVEEIEDLVAR.L |
|  |  |  | 753.3793 | 1504.7441 | 1504.7410 | 63 | 1.6e-005 | K.TGQEVVFVAEPDSK.N |
| Dolichyl-diphosphooligosaccharide-protein glycosyltransferase subunit 1 | Q9GMB0 | 144 | **Observed** | **Mr(expt)** | **Mr(calc)** | **Score** | **Exp Value** | **Peptide** |
|  |  |  | 455.2576 | 908.5007 | 908.4967 | 27 | 0.0058 | K.IDHILDAL.- |
|  |  |  | 476.2600 | 950.5055 | 950.5014 | 32 | 0.0025 | R.FPLFGGWK.T |
|  |  |  | 656.3174 | 1310.6202 | 1310.6143 | 35 | 0.00071 | R.SEDLLDYGPFR.D |
|  |  |  | 710.8600 | 1419.7055 | 1419.6994 | 46 | 8.4e-005 | K.NIQVDSPYEISR.A |
|  |  |  | 740.8965 | 1479.7785 | 1479.7722 | 59 | 2.2e-006 | K.TILPAAAQDVYYR.D |
|  |  |  | 815.4503 | 1628.8860 | 1628.8773 | 61 | 1.2e-006 | R.AASFLLALEPELEAR.L |
| Dolichyl-diphosphooligosaccharide-protein glycosyltransferase subunit STT3A | Q2KJI2 | 54 | **Observed** | **Mr(expt)** | **Mr(calc)** | **Score** | **Exp Value** | **Peptide** |
|  |  |  | 552.2769 | 1102.5392 | 1102.5335 | 36 | 0.00055 | R.FLAEEGFYK.F |
|  |  |  | 652.3349 | 1302.6552 | 1302.6496 | 39 | 0.00034 | R.FYSLLDPSYAK.N |
| Neutral alpha-glucosidase AB | P79403 | 91 | **Observed** | **Mr(expt)** | **Mr(calc)** | **Score** | **Exp Value** | **Peptide** |
|  |  |  | 585.6552 | 1753.9437 | 1753.9363 | 45 | 7.4e-005 | R.LSFQHDPETSVLILR.K |
|  |  |  | 746.0012 | 1491.8307 | 1491.8303 | 55 | 1.4e-005 | R.SWTGLVLACLGVCLG.L |
|  |  |  | 770.5084 | 1540.9032 | 1540.9016 | 33 | 0.00055 | K.IILTARPFRLDLL.E |
|  |  |  | 917.5622 | 1834.8719 | 1834.8711 | 36 | 0.012 | K.DAQHYGGWEHRDLHN.I |
| Dolichol kinase | Q58CR4 | 104 | **Observed** | **Mr(expt)** | **Mr(calc)** | **Score** | **Exp Value** | **Peptide** |
|  |  |  | 596.8368 | 1191.6590 | 1191.6499 | 43 | 9.9e-005 | R.SLLSLFLDER.D |
|  |  |  | 1114.5059 | 2228.8045 | 2228.8039 | 87 | 3e-008 | R.MSANSGLLPASVVMPLLGLVMK.E |
| DnaJ homolog subfamily B member 11 | P81999 | 91 | **Observed** | **Mr(expt)** | **Mr(calc)** | **Score** | **Exp Value** | **Peptide** |
|  |  |  | 886.4332 | 1770.8518 | 1770.8312 | 73 | 3.2e-007 | K.FQDLGAAYEVLSDSEK.R |
|  |  |  | 947.7625 | 1895.0816 | 1895.0809 | 47 | 0.00066 | R.GDDLYTNVTISLVESLVG.F |
| Sarcoplasmic/endoplasmic reticulum calcium ATPase | O46674 | 176 | **Observed** | **Mr(expt)** | **Mr(calc)** | **Score** | **Exp Value** | **Peptide** |
|  |  |  | 554.2591 | 1106.5036 | 1106.4993 | 28 | 0.0021 | R.EWGSGSDTLR.C |
|  |  |  | 704.8545 | 1407.6945 | 1407.6882 | 67 | 5.1e-007 | R.IGIFGQDEDVTSK.A |
|  |  |  | 736.4290 | 1470.8435 | 1470.8367 | 72 | 6.1e-008 | K.ISLPVILMDETLK.F |
|  |  |  | 787.9385 | 1573.8625 | 1573.8563 | 72 | 7.3e-008 | R.VDQSILTGESVSVIK.H |
| Signal recognition particle receptor subunit alpha | Q3MHE8 | 72 | **Observed** | **Mr(expt)** | **Mr(calc)** | **Score** | **Exp Value** | **Peptide** |
|  |  |  | 624.8233 | 1247.6321 | 1247.6260 | 55 | 1.1e-005 | -.MLDFFTIFSK.G |
|  |  |  | 632.8173 | 1263.6201 | 1263.6210 | 41 | 0.00021 | -.MLDFFTIFSK.G |
|  |  |  | 687.3565 | 1372.6984 | 1372.6909 | 16 | 0.04 | K.VMGTFSTVTSTVK.Q |
| Tubulin beta-5 chain | Q2KJD0 | 281 | **Observed** | **Mr(expt)** | **Mr(calc)** | **Score** | **Exp Value** | **Peptide** |
|  |  |  | 520.3028 | 1038.5911 | 1038.5862 | 65 | 6.9e-006 | R.YLTVAAVFR.G |
|  |  |  | 565.8031 | 1129.5917 | 1129.5880 | 61 | 3.9e-005 | R.FPGQLNADLR.K |
|  |  |  | 580.3205 | 1158.6264 | 1158.6219 | 48 | 0.00071 | K.LAVNMVPFPR.L |
|  |  |  | 615.3061 | 1228.5976 | 1228.5910 | 59 | 3.7e-005 | R.ISEQFTAMFR.R |
|  |  |  | 651.3248 | 1300.6351 | 1300.6299 | 46 | 0.0007 | R.ISVYYNEATGGK.Y |
|  |  |  | 723.8509 | 1445.6872 | 1445.6820 | 68 | 4.2e-006 | K.EVDEQMLNVQNK.N |
|  |  |  | 731.8480 | 1461.6814 | 1461.6769 | 64 | 8.7e-006 | K.EVDEQMLNVQNK.N |
|  |  |  | 808.4251 | 1614.8357 | 1614.8287 | 38 | 0.0047 | R.AILVDLEPGTMDSVR.S |
|  |  |  | 848.9235 | 1695.8324 | 1695.8257 | 58 | 4.2e-005 | K.NSSYFVEWIPNNVK.T |
|  |  |  | 653.6679 | 1957.9820 | 1957.9745 | 57 | 3.8e-005 | K.GHYTEGAELVDSVLDVVR.K |
| 40s ribosomal protein S9 | A6QLG5 | 106 | **Observed** | **Mr(expt)** | **Mr(calc)** | **Score** | **Exp Value** | **Peptide** |
|  |  |  | 460.7659 | 919.5173 | 919.5127 | 32 | 0.002 | K.LIGEYGLR.N |
|  |  |  | 461.2400 | 920.4654 | 920.4603 | 43 | 0.00025 | K.IEDFLER.R |
|  |  |  | 467.7867 | 933.5588 | 933.5535 | 34 | 0.00062 | K.LDYILGLK.I |
|  |  |  | 516.7982 | 1031.5818 | 1031.5764 | 46 | 9.2e-005 | R.LFEGNALLR.R |
|  |  |  | 605.3518 | 1208.6891 | 1208.6839 | 25 | 0.0032 | K.MKLDYILGLK.I |
|  |  |  | 636.3804 | 1270.7462 | 1270.7398 | 37 | 0.00018 | K.QVVNIPSFIVR.L |
| Myosin light polypeptide 6 | P60661 | 89 | **Observed** | **Mr(expt)** | **Mr(calc)** | **Score** | **Exp Value** | **Peptide** |
|  |  |  | 677.8723 | 1353.7299 | 1353.7252 | 66 | 6.5e-006 | R.ALGQNPTNAEVLK.V |
|  |  |  | 1017.1369 | 2034.1521 | 2034.1515 | 34 | 0.00078 | K.DQGTYEDYVEGLRVFDK.E |
| Cytoplasmic dynein 1 intermediate chain 2 | Q0III3 | 90 | **Observed** | **Mr(expt)** | **Mr(calc)** | **Score** | **Exp Value** | **Peptide** |
|  |  |  | 772.3816 | 1542.7486 | 1542.7413 | 32 | 0.0017 | K.EAVAPVQEESDLEK.K |
|  |  |  | 1065.6992 | 2131.4320 | 2131.4316 | 84 | 6.7e-008 | K.HRVVSCLDWSSQYPELLV.A |
| Calcium-binding protein p22 | Q3SYS6 | 107 | **Observed** | **Mr(expt)** | **Mr(calc)** | **Score** | **Exp Value** | **Peptide** |
|  |  |  | 654.3737 | 1306.7329 | 1306.7245 | 84 | 9e-008 | R.IPELAINPLGDR.I |
|  |  |  | 796.5456 | 1592.8417 | 1592.8411 | 37 | 0.00024 | R.MMVGVNISDEQLGSI.A |
| Transitional endoplasmic reticulum ATPase | Q3ZBT1 | 184 | **Observed** | **Mr(expt)** | **Mr(calc)** | **Score** | **Exp Value** | **Peptide** |
|  |  |  | 525.2821 | 1048.5496 | 1048.5441 | 29 | 0.005 | K.DVDLEFLAK.M |
|  |  |  | 365.5589 | 1093.6548 | 1093.6495 | 14 | 0.039 | R.LEILQIHTK.N |
|  |  |  | 665.3521 | 1328.6897 | 1328.6837 | 67 | 5.9e-007 | R.WALSQSNPSALR.E |
|  |  |  | 778.9359 | 1555.8572 | 1555.8497 | 59 | 1.6e-006 | R.LDQLIYIPLPDEK.S |
|  |  |  | 906.0038 | 1809.9931 | 1809.9876 | 57 | 1.8e-006 | K.NAPAIIFIDELDAIAPK.R |
|  |  |  | 976.4680 | 1950.9215 | 1950.9146 | 60 | 9.9e-007 | K.GPELLTMWFGESEANVR.E |
| Tudor domain-containing protein 3 | Q2HJG4 | 110 | **Observed** | **Mr(expt)** | **Mr(calc)** | **Score** | **Exp Value** | **Peptide** |
|  |  |  | 597.7930 | 1193.5715 | 1193.5789 | 81 | 1.4e-008 | K.ETKTFGGGGGGAR.S |
|  |  |  | 746.8643 | 1491.7140 | 1491.7140 | 50 | 1e-005 | R.HITEMGFSKEASR.Q |
| Testin | Q2QLB2 | 94 | **Observed** | **Mr(expt)** | **Mr(calc)** | **Score** | **Exp Value** | **Peptide** |
|  |  |  | 684.3869 | 1366.7592 | 1366.7569 | 55 | 2.9e-006 | K.NEALGVGDVKLPR.E |
|  |  |  | 1228.9987 | 2457.8911 | 2457.8900 | 47 | 0.00046 | K.CEGFELHFWRKICRNCKCGQ.E |
|  |  |  | 713.2548 | 1426.4911 | 1426.4909 | 44 | 0.00015 | R.VTYNNFSWHAST.E |
| Septin-2 | Q2NKY7 | 91 | **Observed** | **Mr(expt)** | **Mr(calc)** | **Score** | **Exp Value** | **Peptide** |
|  |  |  | 880.4867 | 1758.9589 | 1758.9516 | 60 | 1e-006 | K.ASIPFSVVGSNQLIEAK.G |
|  |  |  | 444.2651 | 886.5157 | 886.5236 | 52 | 7.8e-006 | K.EQTRLLK.A |
| tRNA-splicing ligase RtcB homolog | Q5E9T9 | 65 | **Observed** | **Mr(expt)** | **Mr(calc)** | **Score** | **Exp Value** | **Peptide** |
|  |  |  | 603.8068 | 1205.5991 | 1205.5928 | 45 | 0.0013 | R.NLDFQDVLDK.L |
|  |  |  | 693.3359 | 1384.6573 | 1384.6510 | 48 | 0.00049 | R.SYNDELQFLEK.I |
| Pyrroline-5-carboxylate reductase 2 | Q17QJ7 | 163 | **Observed** | **Mr(expt)** | **Mr(calc)** | **Score** | **Exp Value** | **Peptide** |
|  |  |  | 501.7776 | 1001.5407 | 1001.5368 | 43 | 0.0033 | K.LMAFQPAPK.V |
|  |  |  | 506.8129 | 1011.6111 | 1011.6077 | 76 | 4.1e-007 | R.LGAQALLGAAK.M |
|  |  |  | 586.8272 | 1171.6398 | 1171.6349 | 51 | 0.00027 | R.GFTAAGILSAHK.I |
|  |  |  | 589.7736 | 1177.5326 | 1177.5285 | 39 | 0.0028 | R.ELQSMADQEK.I |
|  |  |  | 718.3487 | 1434.6827 | 1434.6773 | 84 | 9.9e-008 | R.TRELQSMADQEK.I |
|  |  |  | 908.4828 | 1814.9510 | 1814.9448 | 22 | 0.11 | K.IIASSPEMDLPTVSALR.K |
| Very long-chain specific acyl-CoA dehydrogenase | P48818 | 94 | **Observed** | **Mr(expt)** | **Mr(calc)** | **Score** | **Exp Value** | **Peptide** |
|  |  |  | 424.7674 | 847.5203 | 847.5167 | 33 | 0.0008 | K.GILLFGTK.A |
|  |  |  | 501.2869 | 1000.5592 | 1000.5553 | 44 | 0.00021 | K.ELSGLGNALK.N |
|  |  |  | 589.3164 | 1176.6183 | 1176.6139 | 31 | 0.0045 | R.IFEGTNDILR.L |
|  |  |  | 729.3939 | 1456.7732 | 1456.7674 | 59 | 3.1e-006 | K.NPFGNAGLLLGEAGK.Q |
| Isocitrate dehydrogenase | P33198 | 246 | **Observed** | **Mr(expt)** | **Mr(calc)** | **Score** | **Exp Value** | **Peptide** |
|  |  |  | 461.7403 | 921.4660 | 921.4556 | 51 | 0.00015 | K.ATDFVVDR.A |
|  |  |  | 488.7885 | 975.5625 | 975.5502 | 28 | 0.016 | R.NILGGTVFR.E |
|  |  |  | 522.2832 | 1042.5519 | 1042.5407 | 39 | 0.0021 | K.LDGNQDLIR.F |
|  |  |  | 547.7918 | 1093.5690 | 1093.5556 | 31 | 0.01 | K.YFDLGLPNR.D |
|  |  |  | 584.8051 | 1167.5957 | 1167.5812 | 37 | 0.002 | K.DIFQEIFEK.H |
|  |  |  | 614.8426 | 1227.6706 | 1227.6571 | 34 | 0.0048 | R.GKLDGNQDLIR.F |
|  |  |  | 622.8578 | 1243.7010 | 1243.6846 | 58 | 1.6e-005 | R.LIDDMVAQVLK.S |
|  |  |  | 630.8537 | 1259.6928 | 1259.6795 | 57 | 2.3e-005 | R.LIDDMVAQVLK.S |
|  |  |  | 481.9278 | 1442.7615 | 1442.7446 | 37 | 0.0023 | R.FKDIFQEIFEK.H |
|  |  |  | 831.4476 | 1660.8806 | 1660.8573 | 63 | 5.4e-006 | R.PTSTNPIASIFAWTR.G |
|  |  |  | 559.6102 | 1675.8088 | 1675.7909 | 32 | 0.0048 | K.VAKPVVEMDGDEMTR.I |
|  |  |  | 924.4646 | 1846.9146 | 1846.8909 | 82 | 5.4e-008 | R.DQTNDQVTIDSALATQK.Y |
|  |  |  | 636.3311 | 1905.9716 | 1905.9472 | 37 | 0.0011 | K.LNEHFLNTSDFLDTIK.S |
| NADH-ubiquinone oxidoreductase 75kDa subunit | P15690 | 184 | **Observed** | **Mr(expt)** | **Mr(calc)** | **Score** | **Exp Value** | **Peptide** |
|  |  |  | 490.7999 | 979.5852 | 979.5815 | 22 | 0.0058 | K.VAVTPPGLAR.E |
|  |  |  | 532.7824 | 1063.5502 | 1063.5451 | 48 | 6.2e-005 | R.FEAPLFNAR.I |
|  |  |  | 578.3244 | 1154.6342 | 1154.6295 | 44 | 0.00012 | R.LEEVSPNLVR.Y |
|  |  |  | 702.4022 | 1402.7898 | 1402.7820 | 51 | 1.1e-005 | K.VALIGSPVDLTYR.Y |
|  |  |  | 804.9003 | 1607.7860 | 1607.7791 | 107 | 3.6e-011 | R.FASEIAGVDDLGTTGR.G |
| Succinyl-CoA ligase subunit beta | Q3MHX5 | 199 | **Observed** | **Mr(expt)** | **Mr(calc)** | **Score** | **Exp Value** | **Peptide** |
|  |  |  | 397.7258 | 793.4370 | 793.4334 | 17 | 0.052 | K.GVFSSGLK.G |
|  |  |  | 455.7754 | 909.5362 | 909.5324 | 35 | 0.00041 | K.LYNLFLK.I |
|  |  |  | 620.7858 | 1239.5571 | 1239.5520 | 54 | 5.8e-006 | K.INFDDNAEFR.Q |
|  |  |  | 645.3524 | 1288.6903 | 1288.6849 | 68 | 3.8e-007 | R.MAENLGFLGPLK.N |
|  |  |  | 653.3499 | 1304.6853 | 1304.6798 | 37 | 0.00044 | R.MAENLGFLGPLK.N |
|  |  |  | 798.9019 | 1595.7892 | 1595.7831 | 91 | 1.6e-009 | R.FFVADTANEALEAAK.R |
| Single-stranded DNA-binding protein | Q95KK4 | 190 | **Observed** | **Mr(expt)** | **Mr(calc)** | **Score** | **Exp Value** | **Peptide** |
|  |  |  | 493.2546 | 984.4947 | 984.4916 | 23 | 0.016 | R.DVAYQYVK.K |
|  |  |  | 348.8831 | 1043.6274 | 1043.6240 | 23 | 0.0005 | R.ISVFRPGLR.D |
|  |  |  | 889.9525 | 1777.8905 | 1777.8821 | 88 | 2.1e-009 | K.NPVTIFSLATNEMWR.S |
|  |  |  | 897.9488 | 1793.8830 | 1793.8770 | 90 | 1.6e-009 | K.NPVTIFSLATNEMWR.S |
| Thioredoxin-dependent peroxide reductase | P35705 | 176 | **Observed** | **Mr(expt)** | **Mr(calc)** | **Score** | **Exp Value** | **Peptide** |
|  |  |  | 483.7447 | 965.4748 | 965.4706 | 14 | 0.12 | K.EISLDDFK.G |
|  |  |  | 603.8358 | 1205.6570 | 1205.6517 | 44 | 7.7e-005 | K.HLSVNDLPVGR.S |
|  |  |  | 643.3827 | 1284.7509 | 1284.7442 | 56 | 2.6e-006 | R.GLFIIDPNGVIK.H |
|  |  |  | 736.9131 | 1471.8116 | 1471.8035 | 109 | 2.3e-011 | R.DYGVLLEGPGLALR.G |
| Pyruvate carboxylase | Q29RK2 | 366 | **Observed** | **Mr(expt)** | **Mr(calc)** | **Score** | **Exp Value** | **Peptide** |
|  |  |  | 682.3443 | 1362.6740 | 1362.6667 | 73 | 2.1e-006 | K.IAEEFEVELER.G |
|  |  |  | 774.3737 | 1546.7328 | 1546.7263 | 83 | 1.1e-007 | R.AEAEAQAEELSFPR.S |
|  |  |  | 782.9090 | 1563.8035 | 1563.7966 | 73 | 1.4e-006 | K.AYVEANQMLGDLIK.V |
|  |  |  | 827.9099 | 1653.8052 | 1653.7999 | 54 | 0.0001 | K.DFTATFGPLDSLNTR.L |
|  |  |  | 898.9469 | 1795.8793 | 1795.8741 | 73 | 1.7e-006 | K.QVGYENAGTVEFLVDR.H |
|  |  |  | 914.9501 | 1827.8857 | 1827.8792 | 83 | 1e-007 | R.GANAVGYTNYPDNVVFK.F |
|  |  |  | 917.9590 | 1833.9034 | 1833.8971 | 89 | 2.6e-008 | K.YSLQYYMGLAEELVR.A |
|  |  |  | 952.4544 | 1902.8943 | 1902.8860 | 53 | 7.9e-005 | K.ENNVDAVHPGYGFLSER.A |
|  |  |  | 782.4127 | 2344.2162 | 2344.2063 | 70 | 9.3e-007 | R.LDNASAFQGAVISPHYDSLLVK.V |
|  |  |  | 804.7647 | 2411.2724 | 2411.2638 | 61 | 6.4e-006 | R.SVVEFLQGYIGIPHGGFPEPLR.S |
|  |  |  | 760.3968 | 3037.5583 | 3037.5469 | 41 | 0.00044 | R.LQVEHTVTEEITDVDLVHAQIHVAEGR.S |
| Pyruvate dehydrogenase E1 component subunit beta | P11966 | 150 | **Observed** | **Mr(expt)** | **Mr(calc)** | **Score** | **Exp Value** | **Peptide** |
|  |  |  | 410.2538 | 818.4930 | 818.4902 | 28 | 0.0019 | K.DIIFAIK.K |
|  |  |  | 451.7729 | 901.5313 | 901.5273 | 28 | 0.0051 | K.DFLIPIGK.A |
|  |  |  | 621.3432 | 1240.6719 | 1240.6663 | 53 | 9.9e-006 | K.ILEDNSVPQVK.D |
|  |  |  | 624.8212 | 1247.6278 | 1247.6220 | 24 | 0.015 | R.VTGADVPMPYAK.I |
|  |  |  | 901.4578 | 1800.9010 | 1800.8934 | 93 | 8.9e-010 | K.VFLLGEEVAQYDGAYK.V |
| AFG3-like protein 2 | Q2KJI7 | 269 | **Observed** | **Mr(expt)** | **Mr(calc)** | **Score** | **Exp Value** | **Peptide** |
|  |  |  | 542.2793 | 1082.5440 | 1082.5397 | 55 | 0.00011 | R.VSEEIFFGR.I |
|  |  |  | 564.3289 | 1126.6433 | 1126.6386 | 33 | 0.013 | R.QIFIGPPDIK.G |
|  |  |  | 566.3137 | 1130.6128 | 1130.6084 | 44 | 0.002 | K.VGQISFDLPR.Q |
|  |  |  | 677.8405 | 1353.6664 | 1353.6599 | 58 | 5.2e-005 | R.GMGGLFSVGETTAK.V |
|  |  |  | 691.8760 | 1381.7375 | 1381.7315 | 53 | 0.00012 | K.LEIMEFVNFLK.N |
|  |  |  | 598.9634 | 1793.8684 | 1793.8618 | 29 | 0.028 | R.QGDMVLEKPYSEATAR.M |
|  |  |  | 921.9662 | 1841.9179 | 1841.9119 | 109 | 2.7e-010 | R.NLETLQQELGIEGENR.V |
|  |  |  | 957.4784 | 1912.9423 | 1912.9353 | 64 | 8e-006 | K.VTQSAYAQIVQFGMNEK.V |
|  |  |  | 1165.5625 | 2329.1104 | 2329.1015 | 73 | 1.3e-006 | K.TPVDGQYVWFNIGSVDTFER.N |
| Serine beta-lactamase-like protein LACTB | P83095 | 245 | **Observed** | **Mr(expt)** | **Mr(calc)** | **Score** | **Exp Value** | **Peptide** |
|  |  |  | 467.2775 | 932.5404 | 932.5365 | 36 | 0.00066 | K.SLTMVAIAK.L |
|  |  |  | 547.7798 | 1093.5449 | 1093.5404 | 47 | 7.4e-005 | K.FENSIESLR.L |
|  |  |  | 706.8589 | 1411.7032 | 1411.6983 | 38 | 0.00042 | R.LVNTPYVDNSYK.W |
|  |  |  | 760.9126 | 1519.8107 | 1519.8035 | 102 | 7.7e-011 | K.WAGGGFLSTVGDLLK.F |
|  |  |  | 854.4356 | 1706.8567 | 1706.8490 | 56 | 6e-006 | K.FGNAMLYGYQVGLFK.N |
|  |  |  | 862.4031 | 1722.7916 | 1722.7849 | 81 | 1e-008 | K.EVWSEGLGYADVENR.V |
| Aconitate hydratase | P16276 | 197 | **Observed** | **Mr(expt)** | **Mr(calc)** | **Score** | **Exp Value** | **Peptide** |
|  |  |  | 732.3804 | 1462.7462 | 1462.7416 | 52 | 1.4e-005 | K.SQFTITPGSEQIR.A |
|  |  |  | 500.9296 | 1499.7670 | 1499.7620 | 38 | 0.00029 | K.FKLEAPDADELPR.A |
|  |  |  | 834.3899 | 1666.7652 | 1666.7587 | 66 | 4.1e-007 | R.WVVIGDENYGEGSSR.E |
|  |  |  | 877.4460 | 1752.8774 | 1752.8682 | 108 | 3.1e-011 | K.DINQEVYNFLATAGAK.Y |
| Aldehyde dehydrogenase | P20000 | 142 | **Observed** | **Mr(expt)** | **Mr(calc)** | **Score** | **Exp Value** | **Peptide** |
|  |  |  | 408.7494 | 815.4842 | 815.4753 | 28 | 0.03 | R.VTLELGGK.S |
|  |  |  | 520.7987 | 1039.5829 | 1039.5702 | 47 | 0.00016 | K.YGLAAAVFTK.D |
|  |  |  | 702.3926 | 1402.7706 | 1402.7530 | 38 | 0.0013 | K.EEIFGPVMQILK.F |
|  |  |  | 710.3883 | 1418.7621 | 1418.7479 | 36 | 0.0025 | K.EEIFGPVMQILK.F |
|  |  |  | 800.4079 | 1598.8012 | 1598.7828 | 65 | 2.7e-006 | R.ELGEYGLQAYTEVK.T |
|  |  |  | 922.5417 | 1843.0689 | 1843.0455 | 64 | 8.9e-007 | K.VAEQTPLTALYVANLIK.E |
| Medium-chain specific acyl-CoA dehydrogenase | Q3SZB4 | 182 | **Observed** | **Mr(expt)** | **Mr(calc)** | **Score** | **Exp Value** | **Peptide** |
|  |  |  | 617.3296 | 1232.6447 | 1232.6401 | 79 | 3.3e-008 | K.ENVLIGEGAGFK.I |
|  |  |  | 791.9182 | 1581.8219 | 1581.8151 | 74 | 6e-008 | K.IYQIYEGTAQIQR.L |
|  |  |  | 939.4949 | 1876.9753 | 1876.9683 | 73 | 9.2e-008 | K.AFTGFIVEADTPGVQIGR.K |
| NADH dehydrogenase flavoprotein 2 | P04394 | 167 | **Observed** | **Mr(expt)** | **Mr(calc)** | **Score** | **Exp Value** | **Peptide** |
|  |  |  | 608.8223 | 1215.6301 | 1215.6234 | 56 | 9.8e-006 | K.DIEEIIDELK.A |
|  |  |  | 609.3246 | 1216.6346 | 1216.6299 | 76 | 5.3e-008 | R.NSDSILEAIQK.K |
|  |  |  | 668.9040 | 1335.7934 | 1335.7874 | 49 | 2.9e-005 | K.AAAVLPVLDLAQR.Q |
|  |  |  | 828.8935 | 1655.7724 | 1655.7654 | 56 | 3.5e-006 | R.VYEVATFYTMYNR.K |
| Propionyl-CoA carboxylase beta chain | Q2TBR0 | 169 | **Observed** | **Mr(expt)** | **Mr(calc)** | **Score** | **Exp Value** | **Peptide** |
|  |  |  | 777.9091 | 1553.8036 | 1553.7977 | 39 | 0.0027 | K.DTSYLFITGPDVVK.S |
|  |  |  | 852.9083 | 1703.8020 | 1703.7962 | 87 | 4.5e-008 | K.SVTNEDVTQEELGGAR.T |
|  |  |  | 940.9935 | 1879.9724 | 1879.9680 | 98 | 2.8e-009 | R.IQEGVESLAGYADIFLR.N |
| Acyl-coenzyme A thioesterase 9 | Q3SWX2 | 136 | **Observed** | **Mr(expt)** | **Mr(calc)** | **Score** | **Exp Value** | **Peptide** |
|  |  |  | 470.7592 | 939.5038 | 939.5000 | 43 | 0.00016 | R.IFGGFLMR.K |
|  |  |  | 478.7567 | 955.4988 | 955.4950 | 41 | 0.00027 | R.IFGGFLMR.K |
|  |  |  | 687.3920 | 1372.7695 | 1372.7636 | 92 | 1.1e-009 | K.MSPLSIVTALVDK.I |
|  |  |  | 553.9541 | 1658.8405 | 1658.8338 | 30 | 0.0017 | R.TTIHEMFLNTLDPK.T |
| Cytochrome c oxidase subunit 2 | Q2Y0B9 | 100 | **Observed** | **Mr(expt)** | **Mr(calc)** | **Score** | **Exp Value** | **Peptide** |
|  |  |  | 940.9821 | 1879.9496 | 1879.9423 | 90 | 2.1e-008 | R.ILYMMDEINNPSLTVK.T |
|  |  |  | 728.0597 | 2181.1574 | 2181.1504 | 41 | 0.001 | R.MLISSEDVLHSWAVPSLGLK.T |
| Citrate synthase | Q29RK1 | 96 | **Observed** | **Mr(expt)** | **Mr(calc)** | **Score** | **Exp Value** | **Peptide** |
|  |  |  | 499.3023 | 996.5900 | 996.5855 | 34 | 0.0004 | K.DILADLIPK.E |
|  |  |  | 881.9500 | 1761.8854 | 1761.8785 | 83 | 8.9e-009 | K.GLVYETSVLDPDEGIR.F |
| Fumarate hydratase | P10173 | 142 | **Observed** | **Mr(expt)** | **Mr(calc)** | **Score** | **Exp Value** | **Peptide** |
|  |  |  | 610.8205 | 1219.6264 | 1219.6118 | 56 | 4.5e-005 | R.AIEMLGGELGSK.K |
|  |  |  | 882.4786 | 1762.9426 | 1762.9214 | 106 | 1e-010 | R.IYELAAGGTAVGTGLNTR.I |
|  |  |  | 849.4452 | 2545.3137 | 2545.2813 | 31 | 0.0027 | R.THTQDAVPLTLGQEFSGYVQQVK.Y |
| Mitochondrial 2-oxoglutarate/malate carrier protein | P22292 | 129 | **Observed** | **Mr(expt)** | **Mr(calc)** | **Score** | **Exp Value** | **Peptide** |
|  |  |  | 588.7929 | 1175.5712 | 1175.5651 | 24 | 0.0096 | R.YEGFFSLWK.G |
|  |  |  | 605.8482 | 1209.6819 | 1209.6758 | 65 | 3.4e-007 | R.LGIYTVLFER.L |
|  |  |  | 818.4417 | 1634.8688 | 1634.8628 | 84 | 6.6e-009 | R.AVVVNAAQLASYSQSK.Q |
| 10 kDa heat shock protein | P61603 | 84 | **Observed** | **Mr(expt)** | **Mr(calc)** | **Score** | **Exp Value** | **Peptide** |
|  |  |  | 507.2864 | 1012.5582 | 1012.5553 | 43 | 0.0014 | K.GGEIQPVSVK.V |
|  |  |  | 658.3851 | 1314.7556 | 1314.7507 | 72 | 9.1e-007 | K.VLQATVVAVGSGSK.G |
| 60 kDa heat shock protein | P31081 | 500 | **Observed** | **Mr(expt)** | **Mr(calc)** | **Score** | **Exp Value** | **Peptide** |
|  |  |  | 403.2116 | 804.4086 | 804.4051 | 35 | 0.0021 | K.DDAMLLK.G |
|  |  |  | 422.7531 | 843.4917 | 843.4888 | 14 | 0.19 | K.IPAMTIAK.N |
|  |  |  | 451.2731 | 900.5317 | 900.5280 | 56 | 6.9e-006 | K.LSDGVAVLK.V |
|  |  |  | 456.7994 | 911.5843 | 911.5804 | 70 | 1.1e-007 | K.VGLQVVAVK.A |
|  |  |  | 595.8107 | 1189.6068 | 1189.6012 | 40 | 0.00037 | K.EIGNIISDAMK.K |
|  |  |  | 608.3349 | 1214.6552 | 1214.6507 | 39 | 0.00021 | K.NAGVEGSLIVEK.I |
|  |  |  | 695.3590 | 1388.7034 | 1388.6976 | 52 | 2.2e-005 | R.GYISPYFINTSK.G |
|  |  |  | 752.8855 | 1503.7565 | 1503.7490 | 94 | 7.6e-010 | K.TLNDELEIIEGMK.F |
|  |  |  | 549.6418 | 1645.9034 | 1645.8961 | 23 | 0.0051 | K.VGEVIVTKDDAMLLK.G |
|  |  |  | 641.6586 | 1921.9539 | 1921.9455 | 39 | 0.00018 | K.TLNDELEIIEGMKFDR.G |
|  |  |  | 646.9905 | 1937.9497 | 1937.9404 | 27 | 0.0031 | K.TLNDELEIIEGMKFDR.G |
|  |  |  | 678.3916 | 2032.1528 | 2032.1429 | 58 | 1.5e-006 | K.KISSVQSIVPALEIANAHR.K |
|  |  |  | 1026.5265 | 2051.0384 | 2051.0310 | 95 | 4.3e-010 | R.IQEIIEQLDITTSEYEK.E |
|  |  |  | 705.0542 | 2112.1406 | 2112.1323 | 81 | 7.9e-009 | R.ALMLQGVDLLADAVAVTMGPK.G |
|  |  |  | 789.1190 | 2364.3351 | 2364.3264 | 40 | 9.7e-005 | R.KPLVIIAEDVDGEALSTLVLNR.L |
| 28S ribosomal protein S22 | P82649 | 117 | **Observed** | **Mr(expt)** | **Mr(calc)** | **Score** | **Exp Value** | **Peptide** |
|  |  |  | 642.3725 | 1282.7304 | 1282.7245 | 59 | 1.8e-006 | K.IDGLLIDQIQR.D |
|  |  |  | 695.8561 | 1389.6976 | 1389.6922 | 80 | 4.4e-008 | K.LMTQAQLEEATR.Q |
|  |  |  | 533.2741 | 1596.8006 | 1596.7936 | 24 | 0.0063 | K.YVFTDISYSIPHR.E |
| ATP synthase subunit O | Q2EN81 | 115 | **Observed** | **Mr(expt)** | **Mr(calc)** | **Score** | **Exp Value** | **Peptide** |
|  |  |  | 513.3230 | 1024.6315 | 1024.6281 | 42 | 0.00047 | R.VAQILKEPK.V |
|  |  |  | 573.2975 | 1144.5805 | 1144.5764 | 74 | 1.9e-006 | R.YATALYSAASK.Q |
|  |  |  | 637.8359 | 1273.6572 | 1273.6523 | 49 | 0.00048 | K.VDPSIMGGMIVR.I |
|  |  |  | 532.9811 | 1595.9216 | 1595.9147 | 33 | 0.003 | K.LVRPPVQIYGIEGR.Y |
|  |  |  | 620.3417 | 1858.0032 | 1857.9948 | 40 | 0.0012 | K.FSPLTSNLINLLAENGR.L |
| Mitochondrial carrier homolog 2 | Q9N285 | 115 | **Observed** | **Mr(expt)** | **Mr(calc)** | **Score** | **Exp Value** | **Peptide** |
|  |  |  | 420.7233 | 839.4321 | 839.4290 | 42 | 0.00018 | R.GNSLFFR.K |
|  |  |  | 481.2789 | 960.5432 | 960.5393 | 44 | 8.4e-005 | R.GLFTGLTPR.L |
|  |  |  | 759.9237 | 1517.8329 | 1517.8242 | 73 | 5.8e-008 | R.EEGILGFFAGLIPR.L |
| Hexokinase-1 | P27595 | 184 | **Observed** | **Mr(expt)** | **Mr(calc)** | **Score** | **Exp Value** | **Peptide** |
|  |  |  | 406.7761 | 811.5376 | 811.5280 | 35 | 0.00095 | R.LALLQVR.A |
|  |  |  | 514.3226 | 1026.6306 | 1026.6186 | 48 | 8.6e-005 | K.GAALITAVGVR.L |
|  |  |  | 563.2963 | 1124.5780 | 1124.5648 | 70 | 1.4e-006 | K.GAAMVTAVAYR.L |
|  |  |  | 727.8763 | 1453.7379 | 1453.7202 | 72 | 6e-007 | K.GDFIALDLGGSSFR.I |
|  |  |  | 742.4424 | 1482.8703 | 1482.8518 | 67 | 5e-007 | R.SANLVAATLGAILNR.L |
| Glutamate dehydrogenase 1 | P00366 | 108 | **Observed** | **Mr(expt)** | **Mr(calc)** | **Score** | **Exp Value** | **Peptide** |
|  |  |  | 482.2684 | 962.5223 | 962.5185 | 37 | 0.00065 | K.YNLGLDLR.T |
|  |  |  | 500.7331 | 999.4516 | 999.4484 | 45 | 3.3e-005 | K.MVEGFFDR.G |
|  |  |  | 640.9786 | 1919.9139 | 1919.9047 | 25 | 0.0035 | R.DSNYHLLMSVQESLER.K |
|  |  |  | 722.0305 | 2163.0697 | 2163.0630 | 35 | 0.00047 | R.ISGASEKDIVHSGLAYTMER.S |
|  |  |  | 748.0646 | 2241.1719 | 2241.1641 | 52 | 9.2e-006 | K.IIAEGANGPTTPEADKIFLER.N |
| Succinate dehydrogenase flavoprotein subunit | P31039 | 107 | **Observed** | **Mr(expt)** | **Mr(calc)** | **Score** | **Exp Value** | **Peptide** |
|  |  |  | 665.3445 | 1328.6744 | 1328.6684 | 42 | 0.00016 | R.GEGGILINSQGER.F |
|  |  |  | 737.4281 | 1472.8417 | 1472.8351 | 87 | 2.4e-009 | R.LGANSLLDLVVFGR.A |
| Serine hydroxymethyltransferase | Q3SZ20 | 93 | **Observed** | **Mr(expt)** | **Mr(calc)** | **Score** | **Exp Value** | **Peptide** |
|  |  |  | 428.2416 | 854.4687 | 854.4650 | 32 | 0.0014 | R.SGLIFYR.K |
|  |  |  | 593.8583 | 1185.7020 | 1185.6969 | 82 | 5.8e-009 | R.VLELVSITANK.N |
| Acetyl-CoA acetyltransferase | Q29RZ0 | 111 | **Observed** | **Mr(expt)** | **Mr(calc)** | **Score** | **Exp Value** | **Peptide** |
|  |  |  | 838.4773 | 1674.9400 | 1674.9192 | 93 | 1.7e-009 | R.TPIGSFLGSLSSLPATK.L |
|  |  |  | 422.2693 | 842.5240 | 842.5225 | 42 | 0.00016 | K.VLKDAGLK.K |
| NADP transhydrogenase | P11024 | 110 | **Observed** | **Mr(expt)** | **Mr(calc)** | **Score** | **Exp Value** | **Peptide** |
|  |  |  | 821.9112 | 1641.8079 | 1641.8032 | 86 | 3e-009 | R.MATQASTLYSNNITK.L |
|  |  |  | 728.3346 | 1456.6629 | 1456.6612 | 43 | 0.00065 | K.KNFLRTFHTHRI |
|  |  |  | 1008.5403 | 2017.3020 | 2017.3014 | 42 | 0.0021 | R.MLDMFKRPTDPPEYNY.L |
| Sideroflexin-3 | A6QP55 | 132 | **Observed** | **Mr(expt)** | **Mr(calc)** | **Score** | **Exp Value** | **Peptide** |
|  |  |  | 686.3881 | 1370.7616 | 1370.7517 | 80 | 2.6e-008 | R.NLLLSGAQLEASR.N |
|  |  |  | 543.5815 | 1627.7225 | 1627.7155 | 82 | 7.7e-009 | K.YVYDSAFHPDTGEK.V |
| MOSC domain-containing protein 2 | Q1LZH1 | 88 | **Observed** | **Mr(expt)** | **Mr(calc)** | **Score** | **Exp Value** | **Peptide** |
|  |  |  | 745.3860 | 1488.7575 | 1488.7500 | 77 | 3e-008 | K.SSPLFGIYYSVEK.I |
|  |  |  | 908.6504 | 1817.0817 | 1817.0811 | 34 | 0.00068 | K.LLSSNKLHDCRVFGLD.I |
| Dihydrolipoyl dehydrogenase | P09623 | 57 | **Observed** | **Mr(expt)** | **Mr(calc)** | **Score** | **Exp Value** | **Peptide** |
|  |  |  | 791.3942 | 1580.7738 | 1580.7569 | 49 | 9.7e-005 | K.SEEQLKEEGIEYK.V |
|  |  |  | 665.7043 | 1994.0910 | 1994.0659 | 33 | 0.0019 | K.IPNIYAIGDVVAGPMLAHK.A |
| Mitochondrial Rho GTPase 1 | Q2HJF8 | 118 | **Observed** | **Mr(expt)** | **Mr(calc)** | **Score** | **Exp Value** | **Peptide** |
|  |  |  | 448.7837 | 895.5529 | 895.5491 | 38 | 0.0018 | R.ILLVGEPR.V |
|  |  |  | 507.3104 | 1012.6062 | 1012.6029 | 40 | 0.002 | K.SGVLQALLGR.N |
|  |  |  | 688.3509 | 1374.6872 | 1374.6819 | 50 | 0.00031 | K.NISELFYYAQK.A |
|  |  |  | 1137.5668 | 2273.1190 | 2273.1144 | 75 | 5e-007 | R.FGYDDDLDLTPEYLFPLLK.I |
| Tricarboxylate transport protein | P79110 | 66 | **Observed** | **Mr(expt)** | **Mr(calc)** | **Score** | **Exp Value** | **Peptide** |
|  |  |  | 617.8591 | 1233.7036 | 1233.6969 | 43 | 7.9e-005 | R.GLSSLLYGSIPK.A |
|  |  |  | 626.3534 | 1250.6922 | 1250.6871 | 48 | 2.6e-005 | K.GTYQGLTATVLK.Q |
| LETM1 and EF-hand domain-containing protein 1 | Q0VCA3 | 186 | **Observed** | **Mr(expt)** | **Mr(calc)** | **Score** | **Exp Value** | **Peptide** |
|  |  |  | 509.2622 | 1016.5099 | 1016.4968 | 34 | 0.0072 | K.DFSVFFQK.I |
|  |  |  | 650.3875 | 1298.7604 | 1298.7446 | 41 | 0.00032 | K.STLQTLPEIVAK.E |
|  |  |  | 658.8276 | 1315.6407 | 1315.6256 | 73 | 5.2e-007 | K.VAEVEGEQVDNK.A |
|  |  |  | 693.8849 | 1385.7552 | 1385.7402 | 57 | 1.3e-005 | K.LIAEEGVDSLNVK.E |
|  |  |  | 719.3770 | 1436.7395 | 1436.7221 | 56 | 2.3e-005 | K.FLQDTIEEMALK.N |
|  |  |  | 809.4625 | 1616.9105 | 1616.8886 | 66 | 9.2e-007 | K.LLELQSIGTNNFLR.F |
| Cytochrome c1 | P00125 | 62 | **Observed** | **Mr(expt)** | **Mr(calc)** | **Score** | **Exp Value** | **Peptide** |
|  |  |  | 622.3146 | 1863.9219 | 1863.9155 | 33 | 0.00045 | K.LSDYFPKPYPNPEAAR.A |
|  |  |  | 883.4070 | 2647.1993 | 2647.1894 | 48 | 1.6e-005 | K.ALAEEVEVQDGPNEDGEMFMRPGK.L |
| Pyruvate dehydrogenase protein X component | P22439 | 59 | **Observed** | **Mr(expt)** | **Mr(calc)** | **Score** | **Exp Value** | **Peptide** |
|  |  |  | 517.7998 | 1033.5851 | 1033.5808 | 28 | 0.0034 | K.VSVNDFIIK.A |
|  |  |  | 929.9887 | 1857.9628 | 1857.9580 | 53 | 5.4e-006 | K.ILMPSLSPTMEEGNIVK.W |
| Methylmalonate-semialdehyde dehydrogenase | Q07536 | 78 | **Observed** | **Mr(expt)** | **Mr(calc)** | **Score** | **Exp Value** | **Peptide** |
|  |  |  | 597.3002 | 1192.5859 | 1192.5724 | 44 | 0.00061 | K.TLADAEGDVFR.G |
|  |  |  | 944.4773 | 1886.9400 | 1886.9163 | 63 | 3.7e-006 | K.AISFVGSNQAGEYIFER.G |
| Electron transfer flavoprotein subunit alpha | Q2KJE4 | 158 | **Observed** | **Mr(expt)** | **Mr(calc)** | **Score** | **Exp Value** | **Peptide** |
|  |  |  | 684.4199 | 1366.8252 | 1366.8071 | 45 | 6.4e-005 | K.LDVAPISDIIAIK.S |
|  |  |  | 868.5253 | 1735.0360 | 1735.0131 | 52 | 8.2e-006 | K.GLLPEELTPLILATQK.Q |
|  |  |  | 604.9988 | 1811.9745 | 1811.9530 | 31 | 0.0042 | K.LLYDLADQLHAAVGASR.A |
|  |  |  | 1104.5780 | 2207.1414 | 2207.1150 | 93 | 1.7e-009 | K.DPEAPIFQVADYGIVADLFK.V |
|  |  |  | 983.2118 | 2946.6135 | 2946.5742 | 29 | 0.0014 | K.TIVAINKDPEAPIFQVADYGIVADLFK.V |
| SRA stem-loop-interacting RNA-binding protein | Q32P59 | 74 | **Observed** | **Mr(expt)** | **Mr(calc)** | **Score** | **Exp Value** | **Peptide** |
|  |  |  | 615.8302 | 1229.6459 | 1229.6404 | 43 | 0.00015 | K.IPWTAASSELR.E |
|  |  |  | 1009.2017 | 2018.2019 | 2018.2010 | 58 | 2.7e-005 | K.LHVQPQRPKALQGDQTSD.E |
| Peptidyl-tRNA hydrolase 2 | Q3ZBL5 | 60 | **Observed** | **Mr(expt)** | **Mr(calc)** | **Score** | **Exp Value** | **Peptide** |
|  |  |  | 733.4247 | 1464.8348 | 1464.8188 | 47 | 9.5e-005 | R.TVLGIGPGPADLIDK.V |
|  |  |  | 555.9688 | 1664.8847 | 1664.8621 | 40 | 0.00095 | K.APDEETLVELLTHAK.V |
| Sideroflexin-1 | B2LU20 | 78 | **Observed** | **Mr(expt)** | **Mr(calc)** | **Score** | **Exp Value** | **Peptide** |
|  |  |  | 555.2745 | 1108.5345 | 1108.5302 | 36 | 0.00077 | R.WDQSTFIGR.A |
|  |  |  | 560.2846 | 1118.5546 | 1118.5509 | 72 | 1.6e-006 | K.HFFTVTDPR.N |
| Pentatricopeptide repeat-containing protein 3 | Q2KI62 | 100 | **Observed** | **Mr(expt)** | **Mr(calc)** | **Score** | **Exp Value** | **Peptide** |
|  |  |  | 516.9444 | 1547.8113 | 1547.8051 | 65 | 4.4e-007 | R.SDLKEEILMLMAR.D |
|  |  |  | 1064.6549 | 2129.4310 | 2129.4304 | 32 | 0.002 | K.DIAEPHIPCLMPEYFEPQ.I |
|  |  |  | 955.2580 | 1910.2122 | 1910.2116 | 48 | 3.3e-005 | R.EMKAIGIEPSLATYHHI.I |
| Cytochrome c oxidase subunit 4 isoform 1 | Q9TTT8 | 81 | **Observed** | **Mr(expt)** | **Mr(calc)** | **Score** | **Exp Value** | **Peptide** |
|  |  |  | 517.2893 | 1032.5640 | 1032.5604 | 70 | 3.9e-006 | K.VSPIQGFSAK.W |
|  |  |  | 636.3793 | 1270.7441 | 1270.7398 | 42 | 0.0018 | R.AHGSVVKSEDYALPSY.V |
| Sarcoplasmic/endoplasmic reticulum calcium ATPase 2 | Q00779 | 176 | **Observed** | **Mr(expt)** | **Mr(calc)** | **Score** | **Exp Value** | **Peptide** |
|  |  |  | 554.2591 | 1106.5036 | 1106.4993 | 28 | 0.0021 | R.EWGSGSDTLR.C |
|  |  |  | 704.8545 | 1407.6945 | 1407.6882 | 67 | 5.1e-007 | R.IGIFGQDEDVTSK.A |
|  |  |  | 736.4290 | 1470.8435 | 1470.8367 | 72 | 6.1e-008 | K.ISLPVILMDETLK.F |
|  |  |  | 787.9385 | 1573.8625 | 1573.8563 | 72 | 7.3e-008 | R.VDQSILTGESVSVIK.H |
| Lon protease homolog | Q59HJ6 | 118 | **Observed** | **Mr(expt)** | **Mr(calc)** | **Score** | **Exp Value** | **Peptide** |
|  |  |  | 594.3499 | 1186.6853 | 1186.6710 | 37 | 0.0019 | R.DIIALNPLYR.E |
|  |  |  | 601.3815 | 1200.7485 | 1200.7329 | 68 | 1.7e-007 | K.LSSDVLTLLIK.Q |
|  |  |  | 685.3440 | 1368.6734 | 1368.6595 | 61 | 6.3e-006 | R.FSVGGMTDVAEIK.G |
|  |  |  | 799.8888 | 1597.7630 | 1597.7480 | 28 | 0.011 | R.MEMINVSGYVAQEK.L |
| Lipoamide acyltransferase component of branched-chain alpha-keto acid dehydrogenase complex | P11181 | 118 | **Observed** | **Mr(expt)** | **Mr(calc)** | **Score** | **Exp Value** | **Peptide** |
|  |  |  | 480.2629 | 958.5113 | 958.5083 | 55 | 0.0002 | K.LSDIGEGIR.E |
|  |  |  | 565.3115 | 1128.6084 | 1128.6042 | 25 | 0.17 | K.LSFMPFFLK.A |
|  |  |  | 497.6223 | 1489.8451 | 1489.8391 | 33 | 0.0049 | R.ILKEDILNYLEK.Q |
|  |  |  | 827.4342 | 1652.8539 | 1652.8483 | 95 | 8.1e-009 | K.SYLENPAFMLLDLK.- |
| 2-oxoglutarate dehydrogenase | Q148N0 | 281 | **Observed** | **Mr(expt)** | **Mr(calc)** | **Score** | **Exp Value** | **Peptide** |
|  |  |  | 485.7449 | 969.4752 | 969.4709 | 40 | 0.0037 | K.SWDIFFR.N |
|  |  |  | 499.2768 | 996.5390 | 996.5352 | 22 | 0.23 | K.THLTELQR.L |
|  |  |  | 506.3211 | 1010.6276 | 1010.6236 | 55 | 2.8e-005 | R.LNVLANVIR.K |
|  |  |  | 375.8858 | 1124.6355 | 1124.6302 | 30 | 0.026 | K.KTHLTELQR.L |
|  |  |  | 595.7828 | 1189.5510 | 1189.5462 | 69 | 3.6e-006 | K.LEAADEGSGDVK.Y |
|  |  |  | 684.8588 | 1367.7030 | 1367.6973 | 79 | 3.7e-007 | R.LLDTAFDLDAFK.N |
|  |  |  | 736.3536 | 1470.6926 | 1470.6878 | 65 | 6e-006 | K.LGFYGLDESDLDK.V |
|  |  |  | 498.2894 | 1491.8464 | 1491.8409 | 32 | 0.0064 | K.LVEDHLAVQSLIR.A |
|  |  |  | 965.9885 | 1929.9624 | 1929.9546 | 69 | 2.5e-006 | R.TVDWALAEYMAFGSLLK.E |
|  |  |  | 792.7612 | 2375.2617 | 2375.2519 | 50 | 7.2e-005 | R.NITLSLVANPSHLEAADPVVMGK.T |
|  |  |  | 798.0924 | 2391.2555 | 2391.2468 | 49 | 0.00011 | R.NITLSLVANPSHLEAADPVVMGK.T |
|  |  |  | 800.6621 | 3198.6192 | 3198.6045 | 39 | 0.00081 | R.GHHVAQLDPLGILDADLDSSVPADIISSTDK.L |
| Sorting and assembly machinery component 50 homolog | Q2HJ55 | 190 | **Observed** | **Mr(expt)** | **Mr(calc)** | **Score** | **Exp Value** | **Peptide** |
|  |  |  | 492.7629 | 983.5112 | 983.5076 | 28 | 0.0045 | R.NFSVNLYK.V |
|  |  |  | 548.7949 | 1095.5752 | 1095.5713 | 39 | 0.00033 | R.FYLGGPTSVR.G |
|  |  |  | 561.3111 | 1120.6076 | 1120.6029 | 41 | 0.00026 | R.WSYGAGIVLR.L |
|  |  |  | 589.3008 | 1176.5870 | 1176.5815 | 39 | 0.00052 | K.VTFQFSYGTK.E |
|  |  |  | 639.3386 | 1276.6625 | 1276.6564 | 55 | 7.9e-006 | K.VTGQFPWSSLR.E |
|  |  |  | 1093.0399 | 2184.0652 | 2184.0555 | 106 | 3.7e-011 | R.LTGSYNTMVGNNEGSMVLGLK.L |
| ATP synthase subunit d | P13620 | 91 | **Observed** | **Mr(expt)** | **Mr(calc)** | **Score** | **Exp Value** | **Peptide** |
|  |  |  | 758.9215 | 1515.8283 | 1515.8086 | 65 | 2.3e-006 | K.TIDWVAFGEIIPR.N |
|  |  |  | 1247.1323 | 2492.2500 | 2492.2145 | 52 | 2.3e-005 | R.NIIPFDQMTIEDLNEVFPETK.L |
| ATP synthase subunit e | Q9MYT8 | 99 | **Observed** | **Mr(expt)** | **Mr(calc)** | **Score** | **Exp Value** | **Peptide** |
|  |  |  | 588.8784 | 1175.7423 | 1175.7278 | 48 | 1.6e-005 | M.VPPVQVSPLIK.L |
|  |  |  | 680.3600 | 1358.7055 | 1358.7234 | 83 | 6.6e-008 | R.YSALFLGVAYGAK.R |
| 28S ribosomal protein S23 | Q2NL27 | 87 | **Observed** | **Mr(expt)** | **Mr(calc)** | **Score** | **Exp Value** | **Peptide** |
|  |  |  | 527.8371 | 1053.6596 | 1053.6546 | 69 | 1.3e-007 | K.ALLAEGVILR.R |
|  |  |  | 606.8091 | 1211.6036 | 1211.5975 | 39 | 0.00024 | K.AFDLFNPNFK.S |
| NADH dehydrogenase 1 alpha subcomplex subunit 4 | Q01321 | 59 | **Observed** | **Mr(expt)** | **Mr(calc)** | **Score** | **Exp Value** | **Peptide** |
|  |  |  | 611.2958 | 1220.5771 | 1220.5713 | 43 | 0.00013 | K.FYSVNVDYSK.L |
|  |  |  | 716.8679 | 1431.7212 | 1431.7147 | 40 | 0.0002 | R.LALFNPDVSWDR.K |
| Succinate dehydrogenase iron-sulfur subunit | Q3T189 | 72 | **Observed** | **Mr(expt)** | **Mr(calc)** | **Score** | **Exp Value** | **Peptide** |
|  |  |  | 569.8014 | 1137.5883 | 1137.5818 | 30 | 0.0017 | K.LQDPFSLYR.C |
|  |  |  | 836.9183 | 1671.8220 | 1671.8144 | 63 | 1.7e-006 | K.DLVPDLSNFYAQYK.S |
| 39S ribosomal protein L43 | Q95KE5 | 84 | **Observed** | **Mr(expt)** | **Mr(calc)** | **Score** | **Exp Value** | **Peptide** |
|  |  |  | 608.8452 | 1215.6758 | 1215.6711 | 72 | 9.1e-007 | K.SVEEIATLVQK.L |
|  |  |  | 593.8275 | 1185.6404 | 1185.6353 | 43 | 0.0014 | K.LADQSGLDVIR.I |
| 28S ribosomal protein S28 | P82928 | 60 | **Observed** | **Mr(expt)** | **Mr(calc)** | **Score** | **Exp Value** | **Peptide** |
|  |  |  | 530.3078 | 1058.6010 | 1058.5972 | 50 | 0.00032 | R.LLDLELTSR.F |
|  |  |  | 840.7895 | 2519.3466 | 2519.3370 | 40 | 0.00055 | R.FLGATTDTTILEAEAVLLGLQESK.D |
| Up-regulated during skeletal muscle growth protein5 | Q3ZBI7 | 86 | **Observed** | **Mr(expt)** | **Mr(calc)** | **Score** | **Exp Value** | **Peptide** |
|  |  |  | 611.3009 | 1220.5873 | 1220.5826 | 51 | 0.00022 | K.YFNSYTLTGR.M |
|  |  |  | 450.1492 | 900.0738 | 900.0725 | 60 | 1.8e-005 | K.LRSKKTPA.V |
| 4-aminobutyrate aminotransferase | Q9BGI0 | 113 | **Observed** | **Mr(expt)** | **Mr(calc)** | **Score** | **Exp Value** | **Peptide** |
|  |  |  | 470.8152 | 939.6158 | 939.6229 | 89 | 1.2e-009 | K.LILIARNK.G |
|  |  |  | 1036.1839 | 2071.2709 | 2072.2701 | 45 | 8.3e-005 | R.GNYLVDVDGNRMLDLYSQ.I |
| NADH-ubiquinone oxidoreductase chain 2 | Q330A7 | 95 | **Observed** | **Mr(expt)** | **Mr(calc)** | **Score** | **Exp Value** | **Peptide** |
|  |  |  | 581.3235 | 1160.6325 | 1160.6263 | 76 | 4.1e-008 | K.WMIIQELTK.N |
|  |  |  | 572.8243 | 1143.6341 | 1143.6400 | 53 | 0.0022 | K.IKWQLNNTK.P |
| 3-hydroxyacyl-CoA dehydrogenase type-2 | O02691 | 261 | **Observed** | **Mr(expt)** | **Mr(calc)** | **Score** | **Exp Value** | **Peptide** |
|  |  |  | 592.8474 | 1183.6802 | 1183.6747 | 65 | 3.3e-007 | K.GGIVGMTLPIAR.D |
|  |  |  | 600.8443 | 1199.6740 | 1199.6696 | 69 | 2.2e-007 | K.GGIVGMTLPIAR.D |
|  |  |  | 786.4714 | 1570.9282 | 1570.9195 | 75 | 3.5e-008 | R.VINVNLIGTFNVIR.L |
|  |  |  | 893.4158 | 1784.8170 | 1784.8112 | 75 | 3.5e-008 | R.LVAGEMGQNEPDQGGQR.G |
|  |  |  | 1051.0878 | 2100.1610 | 2100.1541 | 59 | 1.2e-006 | R.VMTIAPGLFGTPLLTTLPDK.V |
| AP-1 complex subunit beta-1 | Q08DS7 | 87 | **Observed** | **Mr(expt)** | **Mr(calc)** | **Score** | **Exp Value** | **Peptide** |
|  |  |  | 784.9570 | 1567.8994 | 1567.8933 | 71 | 8.2e-008 | R.LASQANIAQVLAELK.E |
|  |  |  | 482.2806 | 962.5466 | 962.5437 | 39 | 0.00024 | K.KGEIFELK.A |
| Vacuole membrane protein 1 | Q0VCK9 | 114 | **Observed** | **Mr(expt)** | **Mr(calc)** | **Score** | **Exp Value** | **Peptide** |
|  |  |  | 506.8130 | 1011.6115 | 1011.6077 | 31 | 0.00074 | K.LAVQNLVQK.V |
|  |  |  | 464.7683 | 927.5220 | 927.5290 | 99 | 3.6e-009 | R.QNIVLWR.Q |
| Bactericidal permeability-increasing protein | P17453 | 57 | **Observed** | **Mr(expt)** | **Mr(calc)** | **Score** | **Exp Value** | **Peptide** |
|  |  |  | 364.7510 | 727.4875 | 727.4843 | 37 | 0.00025 | K.LLLELK.H |
|  |  |  | 387.2298 | 772.4450 | 772.4443 | 34 | 0.0025 | K.GLDLSIR.D |
| 60S ribosomal protein L24 | Q66WF5 | 160 | **Observed** | **Mr(expt)** | **Mr(calc)** | **Score** | **Exp Value** | **Peptide** |
|  |  |  | 483.7760 | 965.5375 | 965.5334 | 34 | 0.0012 | K.VFQFLNAK.C |
|  |  |  | 596.8305 | 1191.6464 | 1191.6400 | 40 | 0.00022 | R.QINWTVLYR.R |
|  |  |  | 639.3444 | 1276.6743 | 1276.6697 | 79 | 3.6e-008 | R.AITGASLADIMAK.R |
| 60S ribosomal protein L7a | Q2TBQ5 | 123 | **Observed** | **Mr(expt)** | **Mr(calc)** | **Score** | **Exp Value** | **Peptide** |
|  |  |  | 537.3234 | 1072.6323 | 1072.6281 | 28 | 0.0026 | K.KVVNPLFEK.R |
|  |  |  | 608.8221 | 1215.6297 | 1215.6248 | 24 | 0.014 | K.NFGIGQDIQPK.R |
|  |  |  | 785.4262 | 1568.8378 | 1568.8311 | 98 | 2.1e-010 | K.VPPAINQFTQALDR.Q |
|  |  |  | 604.3465 | 1810.0177 | 1810.0101 | 39 | 0.00014 | R.LKVPPAINQFTQALDR.Q |
